# Map and sequence-based chromosome walking towards cloning of the male fertility restoration gene *Rf5* linked to *R*_*11*_ in sunflower

**DOI:** 10.1038/s41598-020-80659-6

**Published:** 2021-01-12

**Authors:** Guojia Ma, Yunming Long, Qijian Song, Zahirul I. Talukder, Md Shamimuzzaman, Lili Qi

**Affiliations:** 1 Department of Plant Sciences, North Dakota State University, Fargo, ND 58108 USA; 2 USDA-Agricultural Research Service, Soybean Genomics and Improvement Laboratory, 10300 Baltimore Ave., Beltsville, MD 20705 USA; 3 USDA-Agricultural Research Service, Edward T. Schafer Agricultural Research Center, 1616 Albrecht Blvd. N, Fargo, ND 58102-2765 USA

**Keywords:** Genetics, Plant sciences

## Abstract

The nuclear fertility restorer gene *Rf5* in HA-R9, originating from the wild sunflower species *Helianthus annuus*, is able to restore the widely used PET1 cytoplasmic male sterility in sunflowers. Previous mapping placed *Rf5* at an interval of 5.8 cM on sunflower chromosome 13, distal to a rust resistance gene *R*_*11*_ at a 1.6 cM genetic distance in an SSR map. In the present study, publicly available SNP markers were further mapped around *Rf5* and *R*_*11*_ using 192 F_2_ individuals, reducing the *Rf5* interval from 5.8 to 0.8 cM. Additional SNP markers were developed in the target region of the two genes from the whole-genome resequencing of HA-R9, a donor line carrying *Rf5* and *R*_*11*_. Fine mapping using 3517 F_3_ individuals placed *Rf5* at a 0.00071 cM interval and the gene co-segregated with SNP marker S13_216392091. Similarly, fine mapping performed using 8795 F_3_ individuals mapped *R*_*11*_ at an interval of 0.00210 cM, co-segregating with two SNP markers, S13_225290789 and C13_181790141. Sequence analysis identified *Rf5* as a pentatricopeptide repeat-encoding gene. The high-density map and diagnostic SNP markers developed in this study will accelerate the use of *Rf5* and *R*_*11*_ in sunflower breeding.

## Introduction

Cytoplasmic male sterility (CMS) is a phenomenon that destroys a plant’s ability to develop viable pollen, and this phenomenon is commonly seen in higher plants^1^. CMS is maternally transmitted, and its determinants are generated through rearrangements of the mitochondrial genome, which generally lack sequence homology across taxa, suggesting multiple origins^2^. Nuclear fertility restorer genes (*Rf*) can suppress the expression of mitochondrial CMS genes to restore the production of viable pollen. The CMS and nuclear *Rf* gene system is of considerable value for commercial hybrid seed production in crops, particularly in maize, rice, cotton, sunflower, and numerous vegetables^3^.

In sunflowers, more than 70 CMS cytoplasms have been described^4^. However, the global commercial hybrid sunflower seed production industry has been largely relying on a single CMS, PET1, identified from wild *Helianthus petiolaris* subsp. *petiolaris* Nutt. and its corresponding fertility restoration gene *Rf1* from sunflower line T660006-2-1 for over 50 years since their first reports^5–7^. Talukder et al. (2019) investigated 159 male fertility restorer lines widely used in sunflower breeding programs and found that 130 lines (83%) retain the *Rf1* gene^8^. The sole use of the CMS-PET1/*Rf1* system in the global sunflower industry is potentially risky due to genetic vulnerability of sunflower hybrids. Further characterization of currently identified CMS/*Rf* gene systems would shed light on molecular mechanisms of interaction between cytoplasmic and nuclear genes, which could enable better use of currently identified, yet widely used, CMS/*Rf* gene systems and minimize the risk associated with genetic vulnerability^9^.

Seven *Rf* genes (*Rf1*, *Rf3*–*Rf7*, and *Msc1*) have been identified and mapped to different chromosomes equating to linkage groups (LGs) in the sunflower genome so far. RHA 266 derived *Rf1* was first mapped to LG6 on a restriction fragment length polymorphism (RFLP) map^10^. RHA 271 derived *Rf1* was separately mapped to LG13 and LG2 on RFLP-based maps by different research groups^11,12^. Later, *Rf1* from RHA 325 was mapped on combined randomly amplified polymorphic DNA (RAPD) and amplified fragment length polymorphism (AFLP) maps without determined LGs^13,14^. There was discrepancy in naming of sunflower linkage groups before the sunflower public simple sequence repeat (SSR) map was published^15^. The most recent study placed *Rf1* derived from RHA 439 to LG13 on an SSR and target region amplification polymorphism (TRAP)-based map^16^. Talukder et al. (2019) reported that 24 significant single nucleotide polymorphism (SNP) markers from LG13 were associated with *Rf1* in a genome-wide association study^8^. *Msc1* was mapped to LG12 on a RFLP map^17^. *Rf3* from RHA 340 and RHA 280, respectively, were both mapped to LG7 on the SSR maps from two different investigations^18,19^. Both *Rf4* and *Rf6* from wild sunflower *H. maximiliani* and an amphiploid of *H. angustifolius*/P 21, respectively, were mapped to LG3 in two sunflower SSR maps^20,21^. Recently, *Rf5* and *Rf7*, from wild *H. annuus* PI 613748 and RHA 428, were mapped to a location on LG13 close to *Rf1* on SSR- and SSR/SNP-based maps, respectively^8,22^.

Because of its high value in commercial hybrid seed production, the genetic and molecular mechanisms underlying cytonuclear incompatibility have been extensively studied and well characterized in many crop species^2^. The first nuclear fertility restorer gene, *Rf2*, was cloned in maize and encodes a mitochondrial aldehyde dehydrogenase to restore fertility of CMS-T cytoplasm^23,24^. Since then, several *Rf* genes have been cloned in different plant species, including *Rf*-*PPR592* in petunia^25^, *Rfo* (*Rfk1*) in radish^26–28^, *Rf1a*, *Rf1b*, *Rf2*, *Rf4*, *Rf5*, *Rf17*, and *Rf98* in rice^29–37^, *Rf1* in sorghum^38^, and *Rf1* (*bvORF20*) in sugar beet^39^. Except for a few, all cloned *Rf* genes encode proteins containing pentatricopeptide repeat (PPR) motifs^40–42^. The PPR motifs are characterized by tandem arrays of 2–27 repeats each with degenerate 35-amino-acid sequences^43,44^. Most PPR proteins in plants target mitochondria or plastids for RNA processing^41^.

Rust incited by biotrophic fungus *Puccinia helianthi* Schw. is one of the most serious diseases of sunflower and is causing substantial yield and quality losses in many sunflower production countries of the world^45^. Resistance to rust in sunflower is often governed by single dominant genes. Currently, twelve rust resistance genes (*R* genes) have been reported and mapped on the sunflower genome, located on chromosomes 2 (*R*_*5*_), 8 (*R*_*1*_ and *R*_*15*_), 11 (*R*_*12*_ and *R*_*14*_), 13 (*R*_*adv*_, *R*_*4*_, *R*_*11*_, *R*_*13a*_, *R*_*13b*_, and *R*_*16*_), and 14 (*R*_*2*_)^22,46–55^. However, the rapid evolution of novel *P. helianthi* races has rendered many *R* genes ineffective, and only seven of 12 *R* genes (*R*_*11*_, *R*_*12*_, *R*_*13a*_, *R*_*13b*_, and *R*_*14*_–*R*_*16*_) remain effectively resistant to all *P. helianthi* races identified in North America so far^51^. Among six rust *R* genes mapped to chromosome 13, *R*_*11*_ is linked to a male fertility restorer gene, *Rf5*, both of which were transferred from wild *H. annuus* into cultivated sunflower^22,56^.

Previous research mapped *Rf5* and *R*_*11*_ to a 7.1 cM interval, resulting in a large gap between the genes and markers on the SSR map^22^. The objective of this research was to fine-map *Rf5* and *R*_*11*_ through the identification of additional genetic recombinants close to the genes from a large population and to identify genomic fragments carrying *Rf5* using a sequencing-based chromosome walking guided by the two sunflower reference genome assemblies HA412-HO and XRQ^57^. The diagnostic markers developed in this study that are closely linked to or within *R*_*11*_ will facilitate sunflower rust-resistance breeding. In addition, fine mapping of *Rf5* and *R*_*11*_ to a small genomic interval containing few candidate genes would lay the foundation for cloning the genes in the future.

## Results

### Saturation mapping of *Rf5* and* R*_*11*_ region

**Figure 1:**
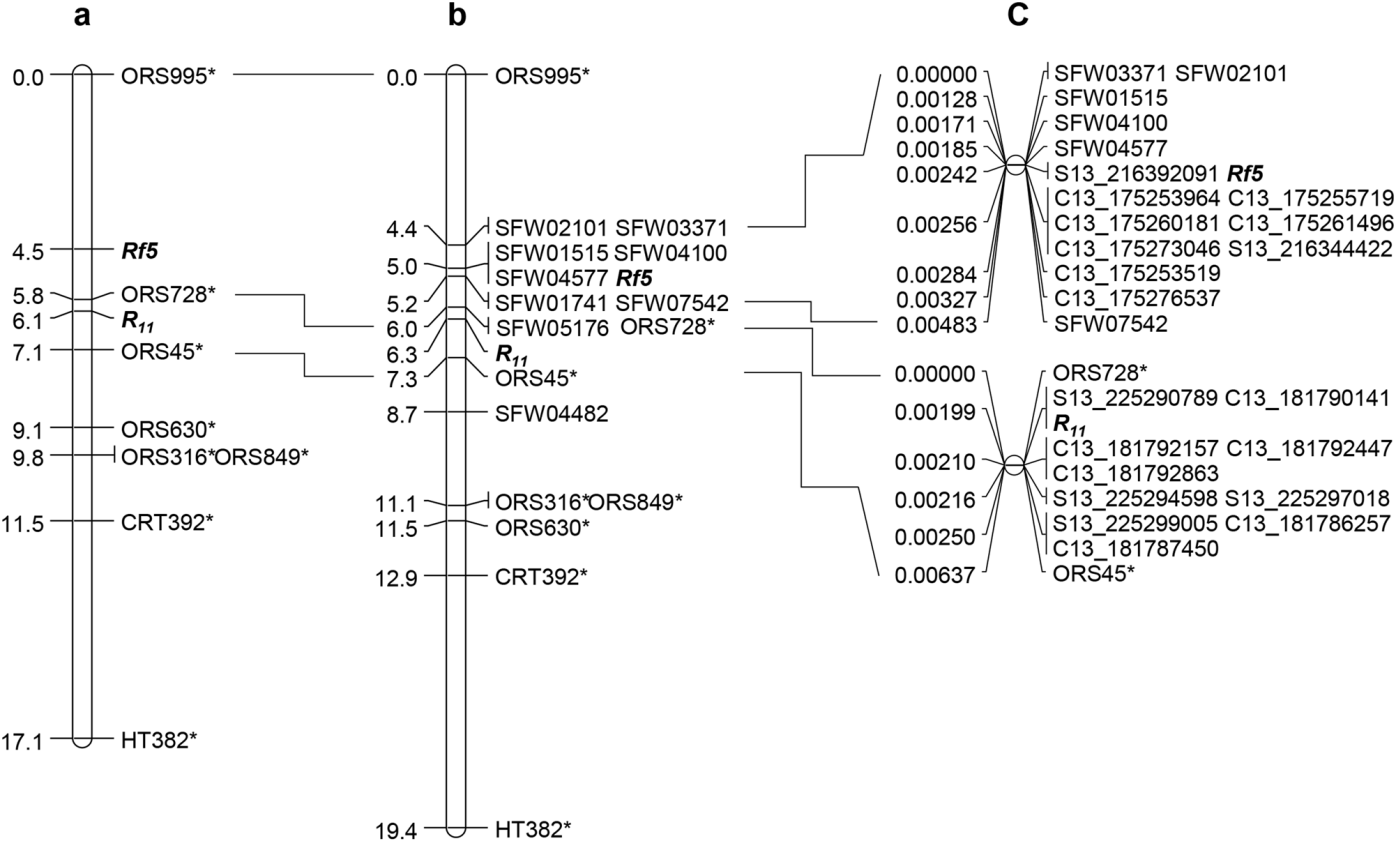
Genetic maps of *Rf5* and *R*_*11*_ on sunflower chromosome 13. (**a**) *Rf5* and *R*_*11*_ basic map^22^; (**b**) *Rf5* and *R*_*11*_ saturation map; and (**c**) *Rf5* and *R*_*11*_ fine maps. *SSR markers.

The previous SSR map placed *Rf5* and *R*_*11*_ to a region of 7.1 cM, with *R*_*11*_ being 1.6 cM proximal to *Rf5*^22^ (Fig. 1a). To saturate *Rf5* and *R*_*11*_ regions, a total of 45 SFW-SNPs most likely to be around both gene loci on sunflower chromosome 13 were selected and converted into the PCR-based length polymorphism markers. The selected 45 SNP markers were screened between two parents, HA 89 and HA-R9, for polymorphism. Nine SFW-SNP markers, SFW01515, SFW01741, SFW02101, SFW03371, SFW04100, SFW04482, SFW04577, SFW05176, and SFW07542, were polymorphic with codominant nature. They were further genotyped in the F_2_ population of 192 individuals. Eight SFW-SNP markers were mapped to the *Rf5* interval between ORS995 and ORS728, reducing the gene interval from 5.8 to 0.8 cM, while no SFW-SNP was mapped to the *R*_*11*_ interval between ORS728 and ORS45 (Fig. 1b). The genetic distance between *Rf5* and *R*_*11*_ was comparable to that of the two genes in the previous SSR map, and three SFW-SNP markers, SFW01515, SFW04100 and SFW04577, were co-segregating with *Rf5* and were 1.3 cM distal to *R*_*11*_^22^ (Fig. 1a,b).

### Fine mapping of *Rf5* and* R*_*11*_ using SNP markers from whole-genome resequencing

#### Recombinant screens from a large population

To increase map resolution, a large population was screened to detect recombinants for both *Rf5* and *R*_*11*_. *Rf5* flanking markers, SNP SFW03371 and SSR ORS728, were used to screen 3517 F_3_ individuals selected from the previously characterized F_2:3_ families heterozygous for *Rf5*. A total of 87 recombinants were identified and were grown in the greenhouse for seeds. Among the 87 recombinants, 24 plants could not develop pollen and were considered sterile. Two fertile plants did not have enough seeds and were later excluded from fertility testing. The remaining 61 fertile recombinant families were grown in the field (35 seeds for each family) to evaluate their genotypes as homozygous or heterozygous, of which 37 were heterozygous fertile, and 24 were homozygous fertile.

Similarly, *R*_*11*_ flanking markers, SSR markers ORS728 and ORS45, were used to screen 8795 F_3_ individuals selected from the previously characterized F_2:3_ families heterozygous for *R*_*11*_ (Fig. 1b). A total of 112 recombinants were identified, and their advanced generation (20 seedlings for each family) was inoculated with *P. helianthi* race 336 for rust resistance testing. Among 112 recombinant families tested, 29 were homozygous susceptible, 18 homozygous resistant, and 65 segregating.

#### New SNP marker development and fine mapping

To further refine the positions of *Rf5* and *R*_*11*_ in the target region, HA-R9 was sequenced at 40 × genome coverage to identify additional SNP markers within the region. The variants, including single nucleotide polymorphisms (SNPs) and insertion-deletions (InDels), were called in the target region of *Rf5* from the two sunflower reference genome assemblies, spanning a 58.2 kb region (216,334,932–216,393,092 bp) on chromosome 13 in the HA412-HO genome and a 60.6 kp region (175,222,724–175,283,334 bp) in the XRQ genome, respectively. A total of 579 variants (536 SNPs and 43 InDels) from HA412-HO and 803 variants (752 SNPs and 51 InDels) from XRQ were identified, respectively. Eighty-four WGS-SNPs (31 from HA412-HO and 53 from XRQ) were selected from the two target regions and screened between the parents, HA 89 and HA-R9, with nine SNPs showing polymorphism. A total of 15 SNP markers (9 WGS-SNPs and 6 SFW-SNPs) were used to genotype 85 recombinants of *Rf5* identified from 3517 F_3_ individuals. Linkage mapping placed *Rf5* on a 0.00071 cM interval on chromosome 13, co-segregating with SNP marker S13_216392091 (Fig. 1c). Most of the WGS-SNP markers were physically positioned in accordance with their genetic positions in the XRQ genome assembly, but genetic and physical positions in the HA412-HO assemblies were reversed in order (Table 1). The flanking markers, S13_216392091 and C13_175253964, delineated *Rf5* to within 35.6 and 30.6 kb regions in the HA412-HO and XRQ assemblies, respectively (Table 1).

**Table 1:** Genetic and physical positions of markers linked to *Rf5* on the fine map of chromosome 13.

Marker	No. recombinations	Genetic distance (cM)	Physical position on XRQ assembly (bp)		Physical position on HA412-HO assembly (bp)	
			Start	End	Start	End
SFW03371		0.00000	169,537,912	169,538,031	220,170,730	220,170,837
SFW02101	0	0.00000	169,600,244	169,600,352	220,283,882	220,283,991
SFW01515	9	0.00128	170,762,684	170,762,803	214,069,367	214,069,487
SFW04100	3	0.00171	172,543,880	172,543,768	218,716,291	218,716,404
SFW04577	1	0.00185	170,914,547	170,914,666	216,832,437	216,832,557
S13_216392091	4	0.00242	175,223,922	175,223,522	216,391,891	216,392,291
***Rf5***	0	0.00242				
C13_175253964	1	0.00256	175,253,764	175,254,164	216,357,107	216,356,707
C13_175255719	0	0.00256	175,255,919	175,255,519	216,355,352	216,354,952
C13_175260181	0	0.00256	175,260,381	175,259,981	216,348,006	216,347,606
C13_175261496	0	0.00256	175,261,296	175,261,696	216,346,692	216,346,292
C13_175273046	0	0.00256	175,273,246	175,272,846	216,344,815	216,344,415
S13_216344422	0	0.00256	175,273,439	175,273,039	216,344,222	216,344,622
C13_175253519	2	0.00284	175,253,319	175,253,719	216,357,552	216,357,152
C13_175276537	3	0.00327	175,276,337	175,276,737	222,305,963	222,306,363
SFW07542	11	0.00483	174,907,936	174,907,818	216,954,392	216,954,277

In the saturation map, the flanking SSR markers, ORS728 and ORS45, delimited *R*_*11*_ to within a 3.4 Mb region (223,364,614–226,744,870 bp) in the HA412-HO assembly with no SNP marker mapped to this interval (Fig. 1b, Table 2). The SNPs/InDels were identified in the *R*_*11*_ target region by aligning HA-R9 sequence to the two reference genomes, spanning a 76.4 kb region (225,224,729–225,301,092 bp) in the HA412-HO assembly and a 1.5 Mb region (180,597,345–182,108,040 bp) in the XRQ assembly. A total of 559 SNPs/InDels were identified in the 76.4 kb region of HA412-HO, and 12,359 SNPs/InDels were found in the 1.5 Mb region of XRQ. A total of 34 SNPs (15 from HA412-HO and 19 from XRQ) were selected to screen between the parents, HA 89 and HA-R9. Ten polymorphic SNPs were used to genotype 112 recombinants of *R*_*11*_ identified from 8795 F_3_ individuals. Fine mapping placed *R*_*11*_ to a 0.00210 cM interval on chromosome 13*,* and the gene co-segregated with S13_225290789 and C13_181790141 (Fig. 1c). The flanking SNP markers, C13_181790141 and C13_181792157 delineated *R*_*11*_ to an interval of 2416 bp in the XRQ genome and 3197 bp in the HA412-HO genome (Table 2). *R*_*11*_ was approximately 8.9 and 6.5 Mb apart from *Rf5* in the HA412-HO and XRQ genome assemblies, respectively (Tables 1 and 2).

**Table 2:** Genetic and physical positions of markers linked to *R*_*11*_ on the fine map of chromosome 13.

Marker	No. recombinations	Genetic distance (cM)	Physical position on XRQ assembly (bp)		Physical position on HA412-HO assembly (bp)	
			Start	End	Start	End
ORS728		0.00000	180,874,637	180,874,661	223,364,590	223,364,614
S13_225290789	35	0.00199	181,793,729	181,793,462	225,290,589	225,290,989
C13_181790141	0	0.00199	181,789,941	181,790,341	225,295,272	225,294,870
***R***_***11***_	0	0.00199				
C13_181792157	2	0.00210	181,791,957	181,792,357	225,292,475	225,292,075
C13_181792447	0	0.00210	181,792,247	181,792,647	225,292,185	225,291,785
C13_181792863	0	0.00210	181,792,663	181,793,063	225,291,769	225,291,366
S13_225294598	1	0.00216	181,790,718	181,790,413	225,294,398	225,294,798
S13_225297018	0	0.00216	181,788,754	181,788,531	225,296,818	225,297,218
S13_225299005	6	0.00250	181,786,867	181,786,467	225,298,805	225,299,205
C13_181786257	0	0.00250	181,786,057	181,786,457	225,299,605	225,299,223
C13_181787450	0	0.00250	181,787,250	181,787,650	225,298,499	225,298,099
ORS45	68	0.00637	–	–	226,744,850	226,744,870

### Candidate genes for *Rf5 *and* R*_*11*_ from the reference genomes

The gene annotation in the 58.2 kb (216,334,932–216,393,092 bp) and 58.8 kb (175,222,724–175,281,566 bp) genomic sequences of HA412-HO and XRQ, respectively, were analyzed on chromosome 13 encompassing the newly identified SNPs closely linked to *Rf5* (https://www.heliagene.org/HA412.v1.1.bronze.20141015/; https://www.heliagene.org/HanXRQ-SUNRISE/). Three and two putative genes were found in the target regions of the HA412-HO and XRQ assemblies, respectively (Table 3). Two genes from the HA412-HO assembly, Ha412v1r1_13g048240 (7171 bp in length) and Ha412v1r1_13g048260 (1878 bp), featured pentatricopeptide repeats, a typical *Rf* gene motif identified from most other crops. One gene, HanXRQChr13g0420371, 40,234 bp in length on the XRQ assembly, also showed a typical tetratricopeptide-like helical domain (Table 3). Using Ha412v1r1_13g048260 sequence (1878 bp) as a query revealed that Ha412v1r1_13g048240 and HanXRQChr13g0420371 share 92 and 100% sequence identity with Ha412v1r1_13g048260, respectively. They were likely to be candidate genes for *Rf5* based on functional domains and physical positions. Other genes in the target region included Ha412v1r1_13g048250, 1598 bp in length in HA412-HO, and HanXRQChr13g0420361, 2365 bp in length in XRQ, and both were predicted to encode AMP-dependent synthetase/ligase (Table 3).

**Table 3:** The candidate genes for the male fertility restoration gene *Rf5* and the rust resistance gene *R*_*11*_ from the reference genomes.

Candidate gene	Description	Physical position	Length (bp)
*Rf5* interval in HA412-HO		216,334,932..216,391,891	56,959
Ha412v1r1_13g048240	Pentatricopeptide repeat	216,342,878..216,350,048	7171
Ha412v1r1_13g048250	AMP-dependent synthetase/ligase	216,351,056..216,352,653	1598
Ha412v1r1_13g048260	Pentatricopeptide repeat	216,354,988..216,356,865	1878
*Rf5* interval in XRQ		175,222,724..175,281,566	58,842
HanXRQChr13g0420361	Putative AMP-dependent synthetase/ligase	175,250,835..175,253,199	2365
HanXRQChr13g0420371	Putative tetratricopeptide-like helical domain	175,253,986..175,294,219	40,234
*R*_*11*_ interval in HA412-HO		225,284,300..225,344,300	60,000
Ha412v1r1_13g051750	UDP-glucuronosyl/UDP-glucosyltransferase	225,284,360..225,286,525	2166
Ha412v1r1_13g051760	UDP-glucuronosyl/UDP-glucosyltransferase	225,291,376..225,292,258	883
*R*_*11*_ interval in XRQ		181,774,000..181,844,981	70,981
HanXRQChr13g0422111	Probable anthocyanidin 3-O-glucosyltransferase 1	181,773,846..181,792,996	19,151
HanXRQChr13g0422121	Putative UDP-glucuronosyl/UDP-glucosyltransferase	181,815,958..181,817,466	1509
HanXRQChr13g0422131	Putative NB-ARC; P-loop containing nucleoside triphosphate hydrolase; Leucine-rich repeat domain, L domain-like	181,835,968..181,841,168	5201

For the gene *R*_*11*_, the 60.0 kb (225,284,300–225,344,300 bp) and 70.981 kb (181,774,000–181,844,981) genomic sequences of HA412-HO and XRQ on chromosome 13 were extracted and analyzed, respectively. Two putative genes, Ha412v1r1_13g051750 (2166 bp) and Ha412v1r1_13g051760 (883 bp), were discovered from the HA412-HO assembly, and both were predicted to code for UDP-glucuronosyl/UDP-glucosyltransferase (Table 3). Three putative genes, HanXRQChr13g0422111, HanXRQChr13g0422121, and HanXRQChr13g0422131, were discovered from the XRQ assembly. Both HanXRQChr13g0422111 (19,151 bp) and HanXRQChr13g0422121 (1509 bp) were predicted to code for glucosyltransferase, while HanXRQChr13g0422131 (5201 bp) was predicted to code for a putative NB-ARC protein (Table 3).

### Sequence comparison of HA-R9 with the candidate genes

HA-R9 whole-genome resequencing generated a total of 487,190,276 raw reads. After removal of 797,556 reads with adapters (0.16%) and 74,547 reads containing > 10% undetermined bases (0.02%), a total of 486,318,173 (99.82%) paired-end clean reads were used for assembly and gap repair. By using SOAPdenovo2^58^, a total of 8,547,762 contigs were constructed, with most contigs (6,933,581 contigs; 81.12%) ranging between 100 and 500 bp in length. Only 20 (0.0002%) and 545,224 (6.38%) contigs were more than 10 and 1 kb in length, respectively. Additionally, a total of 7,010,438 scaffolds were identified, with most of them (6,032,509 scaffolds; 86.05%) ranging between 100 and 500 bp in length, while 11,749 (0.17%) and 565,585 (8.07%) scaffolds were more than 10 and 1 kb in length, respectively. The small size and similar total numbers of contigs and scaffolds were most likely due to the short reads (350 bp of 150 bp paired-end) resulting from the Illumina HiSeq/MiSeq sequencing platform and the wide distribution of repetitive sequences in the sunflower genome.

**Figure 2:**
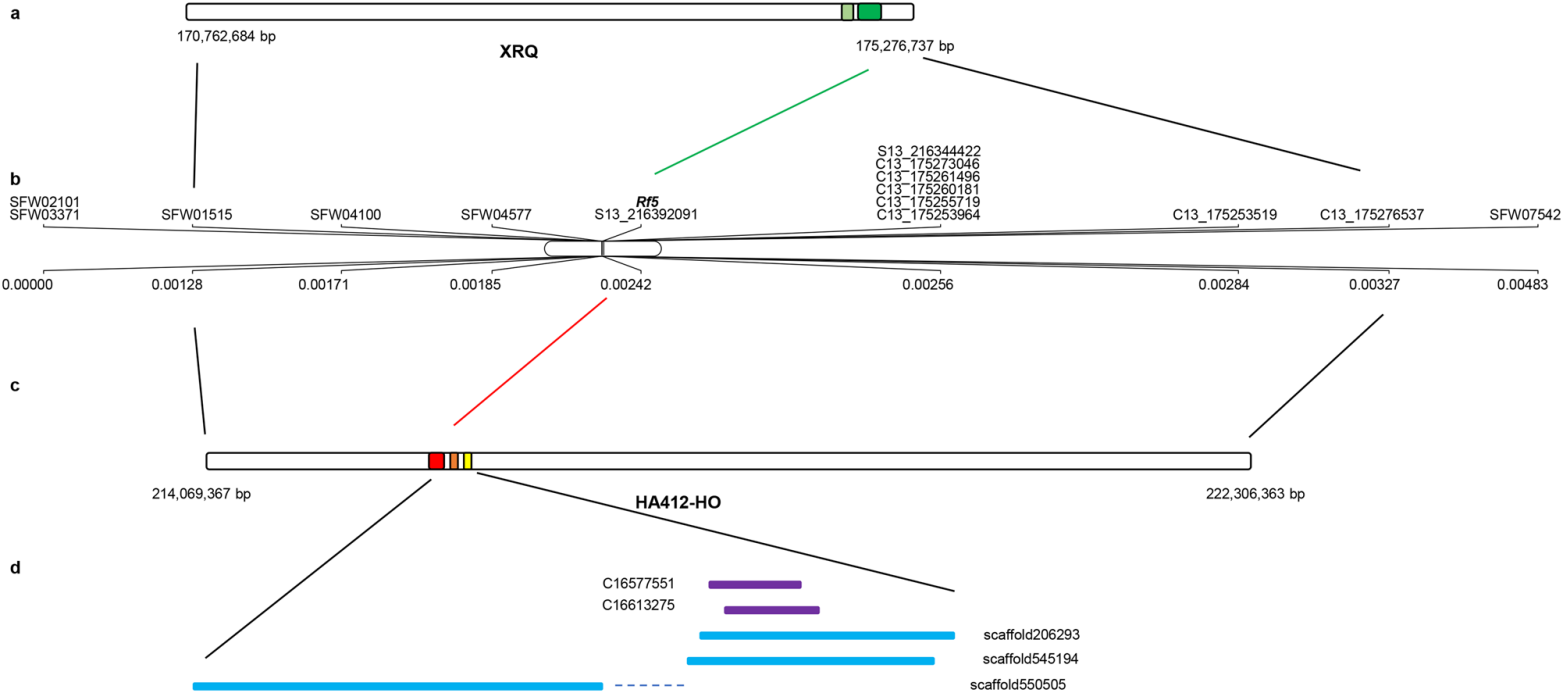
Illustration of *Rf5* gene along sunflower chromosome 13. (**a**) Extracted XRQ genomic region covering *Rf5*. Light and dark green bars highlight candidate *Rf5* genes HanXRQChr13g0420361 and HanXRQChr13g0420371; (**b**) Fine map of *Rf5* on sunflower chromosome 13 showing the relative positions of *Rf5* and its linked markers; (**c**) Extracted HA412-HO genomic region covering *Rf5*. Red, orange and yellow bars highlight candidate *Rf5* genes Ha412v1r1_13g048240, Ha412v1r1_13g048250 and Ha412v1r1_13g048260; (**d**) Solid purple and blue bars show the relative positions of HA-R9 contigs and scaffolds, respectively, aligned to the *Rf5* targeted region of the HA412-HO reference genome, and the dotted line represents the gap.

**Figure 3:**
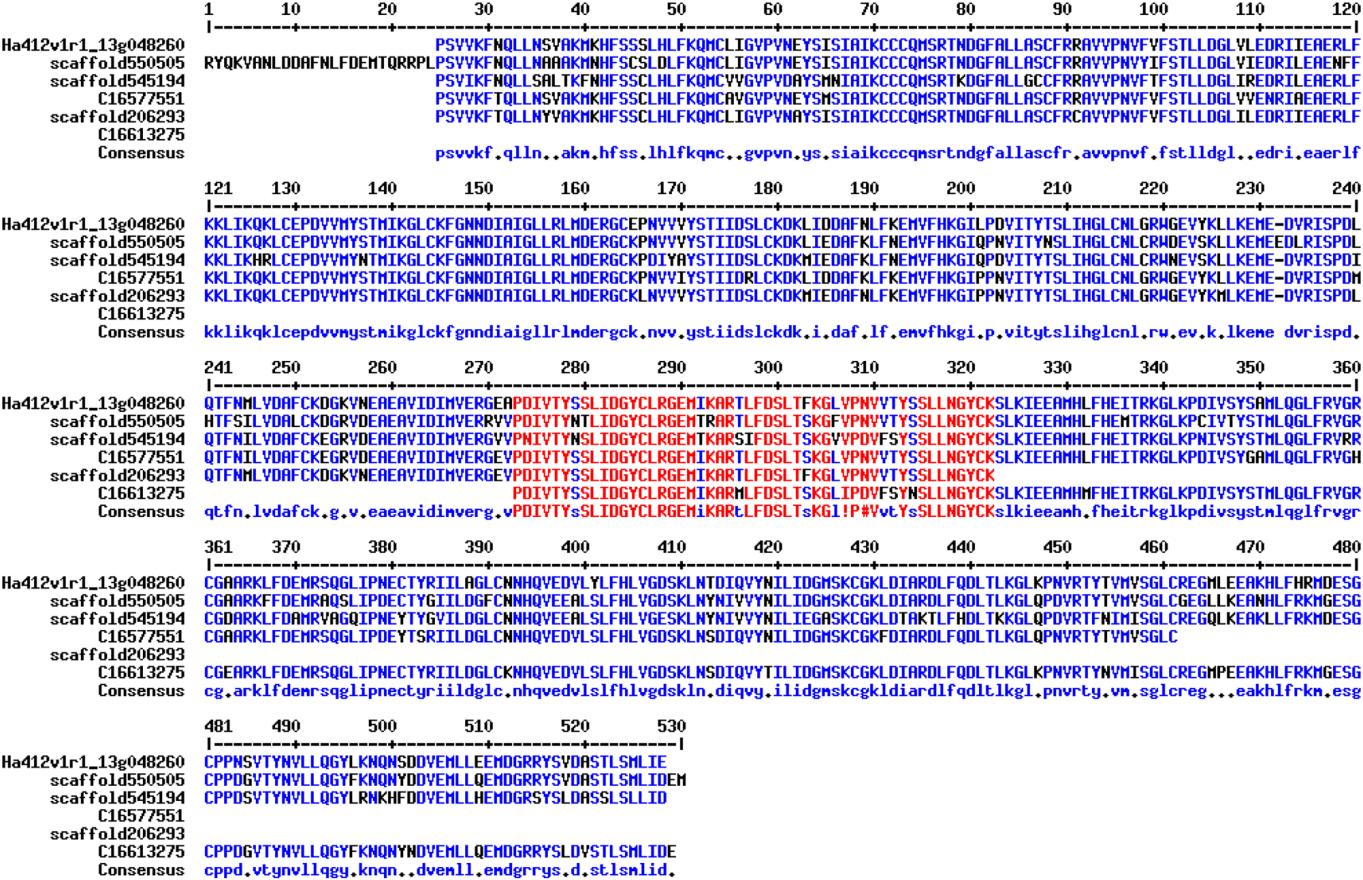
Multiple alignment of amino acid sequences deduced from the best ORFs from two contigs and three scaffolds that are aligned to the *Rf5* candidate gene Ha412v1r1_13g048260.

A stretch of 58.2 kb genomic sequence between 216,334,932 and 216,393,092 bp of chromosome 13 covering the *Rf5* gene was extracted from the reference genome HA412-HO and used as query to search against the HA-R9 assembled contigs and scaffolds. A total of 106 contigs and 393 scaffolds were identified, and 2 contigs (C16577551 and C16613275) and 3 scaffolds (scaffold206293, scaffold545194, and scaffold550505) were selected based on their positions in the target region (Fig. 2). The selected contigs and scaffolds were aligned to the three candidate *Rf5* genes, Ha412v1r1_13g048240, Ha412v1r1_13g048260, and HanXRQChr13g0420371, and showed high levels of sequence identity (Table 4). Contig C16613275 shared the highest level (99%) of identity with candidate gene Ha412v1r1_13g048240, followed by scaffold206293 with Ha412v1r1_13g048260 (97%) and HanXRQChr13g0420371 (97%). The aligned sequence between contig/scaffold and candidate gene was usually over 1 kb. Open reading frames (ORFs) were analyzed using ORFfinder (https://www.ncbi.nlm.nih.gov/orffinder/) among the selected contigs and scaffolds, and the longest ORF for each contig/scaffold was further analyzed by repeat and deduced amino acid numbers (Table 5). Not surprisingly, all of them belong to PPR superfamily with a series of degenerate 35-amino-acid repeats with different copy numbers, suggesting their candidacies for the *Rf5* gene. The best ORFs from each contig/scaffold were aligned, and high similarity was found among them (Fig. 3).

**Table 4:** Summary of the sequence identify between the *Rf5* candidate genes and selected scaffolds and contigs from the HA-R9 whole-genome resequence.

Candidate gene		Contig and scaffold		% identity	Alignment length (bp)	E-value
Query id	Length (bp)	Subject id	Length (bp)			
Ha412v1r1_13g048240	7171	scaffold206293	3988	91	1048	0
		scaffold550505	6443	90	1579	0
		scaffold545194	3878	87	1786	0
		C16577551	1435	91	1420	0
		C16613275	1480	99	759	0
Ha412v1r1_13g048260	1878	scaffold206293	3988	97	1190	0
		scaffold550505	6443	91	1723	0
		scaffold545194	3878	87	1719	0
		C16577551	1435	95	1437	0
		C16613275	1480	94	956	0
HanXRQChr13g0420371	40,234	scaffold206293	3988	97	1829	0
		scaffold550505	6443	93	2072	0
		scaffold545194	3878	87	1927	0
		C16577551	1435	95	1434	0
		C16613275	1480	95	1186	0

**Table 5:** Prediction of the candidate gene for *Rf5.*

Sequences/Line	Alignment Length to Ha412v1r1_13g048260 (bp)	Domain hits	Longest ORF (bp)	# of aa	# of repeats
Ha412v1r1_13g048260/HA412-HO	1878	PPR superfamily	1878	626	14
scaffold206293/HA-R9	1190	PPR superfamily	1188, incomplete	396	8
scaffold550505/HA-R9	1716	PPR superfamily	1836	612	15
scaffold545194/HA-R9	1700	PPR superfamily	1869	623	14
C16577551/HA-R9	1434	PPR superfamily	1362	454	12
C16613275/HA-R9	954	PPR superfamily	885	295	7

Comparative analysis of amino acid sequences of the candidate *Rf5* gene, Ha412v1r1_13g048260, with other characterized Rf orthologues from petunia, radish, rice and sorghum was also performed to reveal the sequence similarity along the PPR motifs. Although multiple sequence alignment showed overall low sequence identity, relatively higher sequence similarity was found in the PPR domains (Supplementary Fig. S1). This comparative analysis indicated that the candidate *Rf5* gene is phylogenetically distant to other characterized *Rf* orthologous from different plant species.

**Figure 4:**
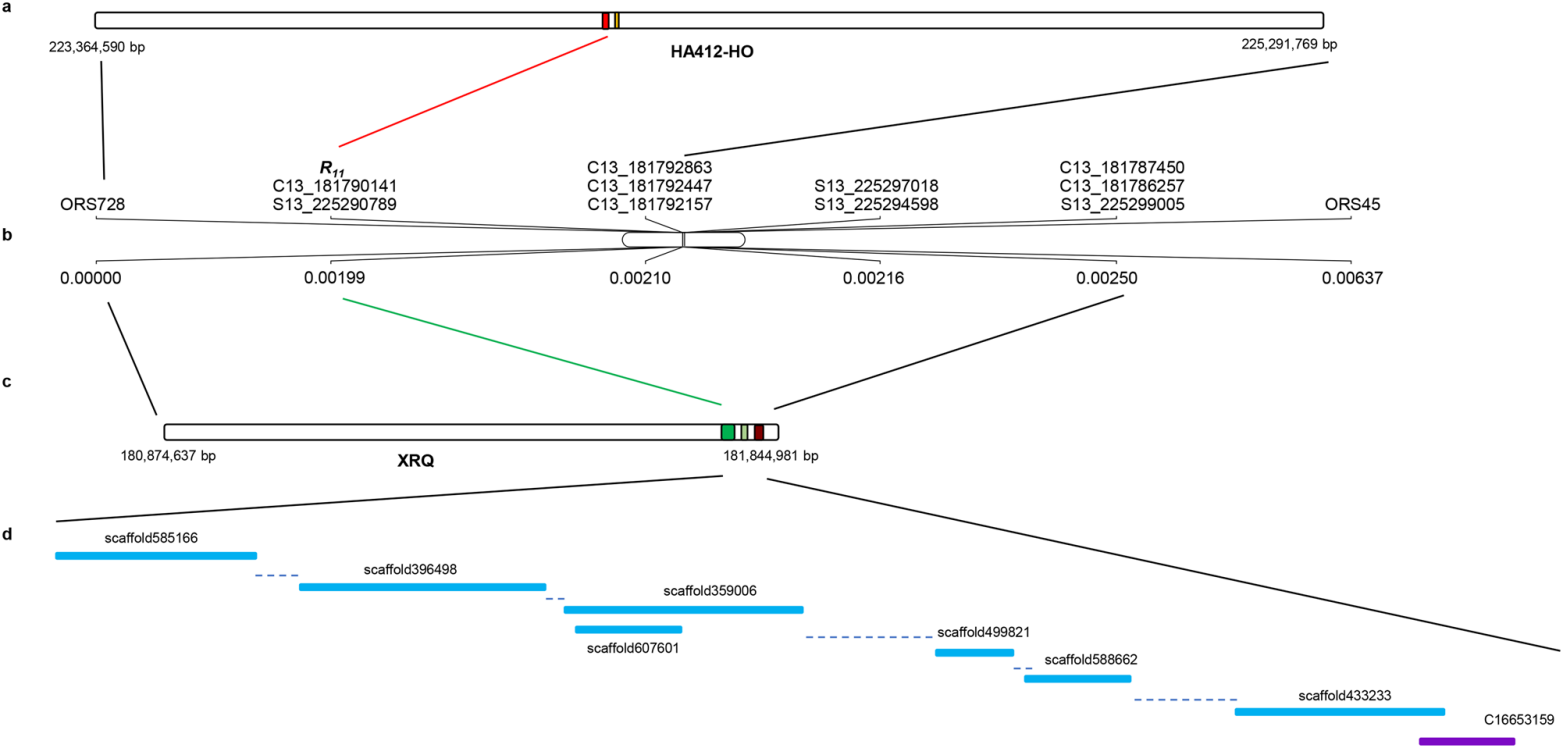
Illustration of *R*_*11*_ gene along sunflower chromosome 13. (**a**) Extracted HA412-HO genomic region containing *R*_*11*_. Red and orange bars on HA412-HO assembly highlight candidate *R*_*11*_ genes Ha412v1r1_13g051750 and Ha412v1r1_13g051760; (**b**) Fine map of *R*_*11*_ on sunflower chromosome 13 showing the relative positions of *R*_*11*_ and its linked markers; (**c**) Extracted XRQ genomic region containing *R*_*11*_. Dark green, light green and brown bars on XRQ assembly highlight candidate *R*_*11*_ genes HanXRQChr13g0422111, HanXRQChr13g0422121, and HanXRQChr13g0422131; and (**d**) Solid purple and blue bars show the relative positions of HA-R9 contigs and scaffolds, respectively, aligned to the *R*_*11*_ targeted region of the XRQ reference genome, and the dotted line represents the gap.

A stretch of 70.981 kb (181,774,000–181,844,981 bp) genomic sequence harboring the *R*_*11*_ gene was extracted from XRQ chromosome 13 and used as a query to search against HA-R9 assembled contigs and scaffolds to identify the *R*_*11*_ gene sequence. After analysis of numerous contigs and scaffolds aligned to the query sequence, one contig and seven scaffolds were selected based on their positions in the target sequence (Fig. 4, Table 6). The selected contig and scaffolds were aligned to the four candidate *R*_*11*_ genes, Ha412v1r1_13g051750, HanXRQChr13g0422111, HanXRQChr13g0422121, and HanXRQChr13g0422131. Scaffold607601 could be aligned to both Ha412v1r1_13g051750 and HanXRQChr13g0422111, respectively, while scaffold585166 and scaffold396498 showed high levels of sequence identity with HanXRQChr13g0422111 only (Table 6). Three scaffolds shared 92 to 98% sequence identity with HanXRQChr13g0422121, while one contig (C16653159) and one scaffold (scaffold433233) showed 95 to 96% sequence identity with HanXRQChr13g0422131.

**Table 6:** Summary of the sequence identify between the *R*_*11*_ candidate genes and selected scaffolds and contigs from the HA-R9 whole-genome resequence.

Candidate Gene		Contig and Scaffold		% identity	Alignment length (bp)	E-value
Query id	Length (bp)	Subject id	Length (bp)			
Ha412v1r1_13g051750	2166	scaffold607601	1724	97	353	5.00E−174
HanXRQChr13g0422111	19,151	scaffold607601	1724	98	852	0
		scaffold585166	3253	97	1745	0
		scaffold396498	4018	97	1928	0
HanXRQChr13g0422121	1509	scaffold359006	3887	92	539	0
		scaffold499821	1257	98	490	0
		scaffold588662	1710	98	713	0
HanXRQChr13g0422131	5201	scaffold433233	3394	96	1999	0
		C16653159	1533	95	1054	0

### SNP marker specificity for *Rf5 *and* R*_*11*_

**Figure 5:**
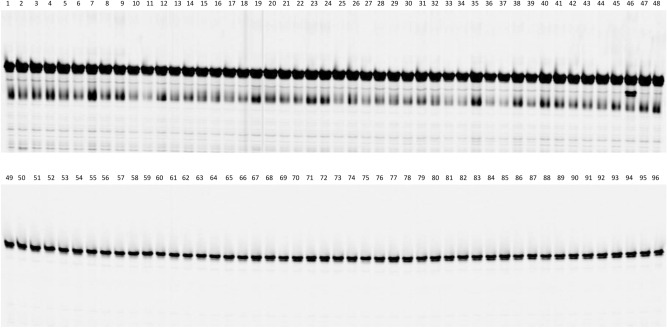
Cropped gel image from PCR amplification of SNP marker C13_175260181 diagnostic for *Rf5* on a panel of 96 diversified sunflower lines including lines with *Rf1* and *Rf7* (Supplementary Table S1). Lane 37 is RHA428 with *Rf7*; lane 46 is HA-R9 carrying *Rf5* and *R*_*11*_, and lane 49 is RHA 464 with *Rf1*. Diagnostic bands were 97 and 102 bp in length including 21/26 bp tail primer, respectively. Full-length gels are presented in Supplementary Figure S2.

The eight SNP markers closely linked to *Rf5* in the fine map were used to screen six sunflower lines: HA 89, HA 234, HA-R9, RHA 397, RHA 428, and RHA 464 (Supplementary Table S1). HA 89 was a recurrent parent for creating HA-R9 with *Rf5*, and HA 234 was a parental line used in mapping *Rf7* from the RHA 428 line. RHA 397 carries an unknown *Rf* gene, while RHA 428 and RHA 464 harbor *Rf7* and *Rf1* mapped to chromosome 13 close to *Rf5*, respectively^8^. Out of eight SNP markers, two exhibited a unique PCR pattern in HA-R9, different from that of the other five lines, and were subsequently used to test a panel of 96 diversified sunflower lines. One dominant SNP marker C13_175260181 was a diagnostic marker for *Rf5* (Fig. 5).

**Figure 6:**
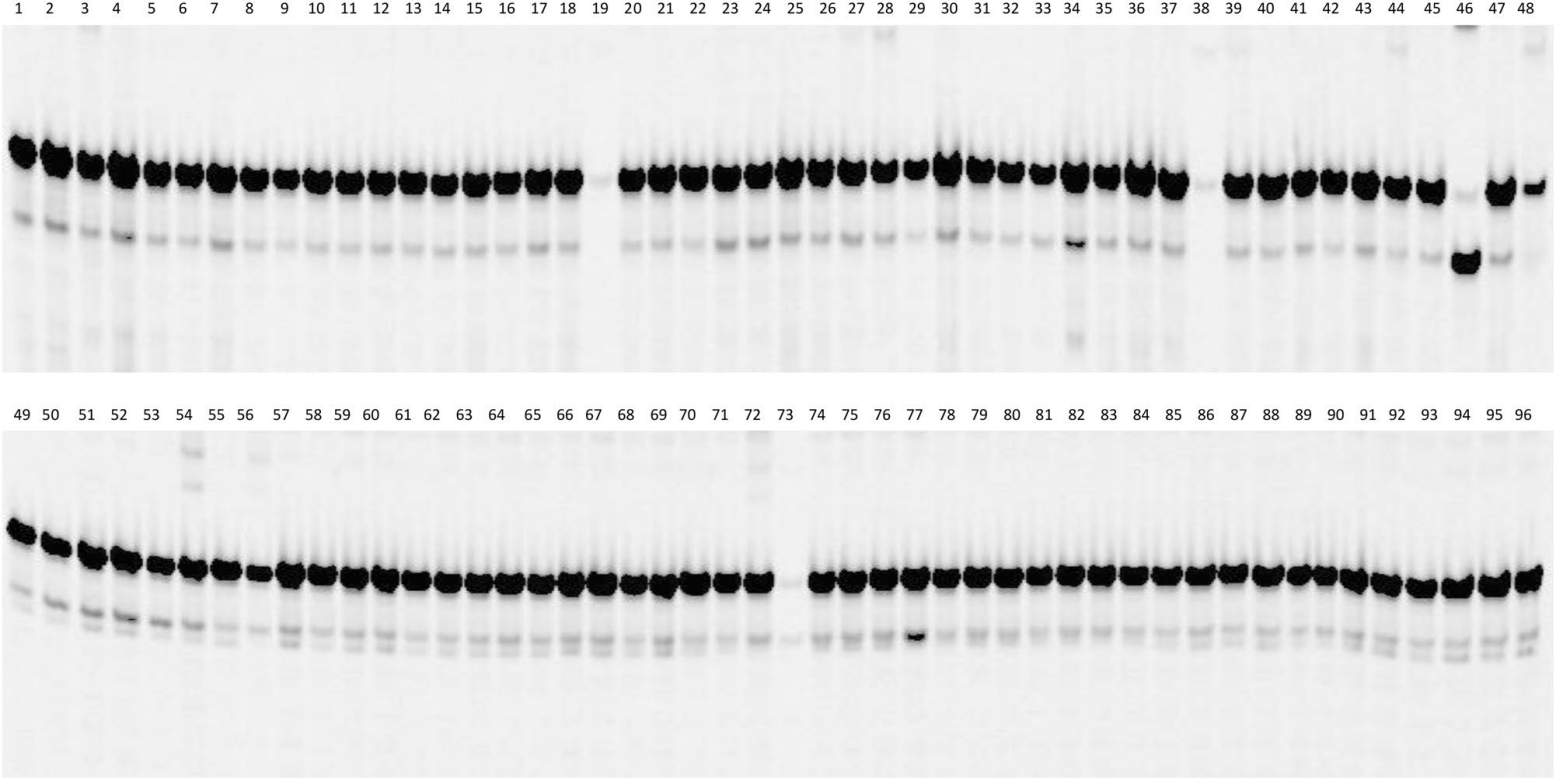
Cropped gel image from PCR amplification of SNP marker C13_181792157 diagnostic for *R*_*11*_ on a panel of 96 diversified sunflower lines. Lane 23 is RHA 340 with *R*_*adv*_; and lane 46 is HA-R9 carrying *Rf5* and *R*_*11*_ (Supplementary Table S1)*.* Bottom diagnostic bands were 115 and 120 bp in length including 21/26 bp tail primer, respectively. Full-length gels are presented in Supplementary Figure S3.

The ten SNP markers closely linked to *R*_*11*_ in the fine map were used to screen six sunflower lines: HA 89, HA-R3, HA-R6, HA-R9, RHA 397, and RHA 464. HA-R3, HA-R6, and RHA 397 carry the rust resistance genes, *R*_*4*_, *R*_*13a*_, and *R*_*13b*_, respectively, all mapped to the lower end of chromosome 13, while RHA 464 harbors a rust *R* gene *R*_*12*_ mapped to chromosome 11^48,51,52^. Among 10 SNPs tested, three exhibited a unique PCR pattern in HA-R9, different from that of the other five lines, and were subsequently used to test a panel of 96 diversified sunflower lines. The two SNPs, C13_181790141 co-segregating with *R*_*11*_ and C13_181792157 proximal to *R*_*11*_ at 0.00011 cM genetic distance were diagnostic markers for *R*_*11*_ (Figs. 1c, 6).

## Discussion

Sunflower chromosome 13, particularly its lower end, harbors a number of economically important genes locating within the cluster. This valuable gene cluster was further divided into two sub-clusters^51^, i.e. sub-cluster I including the rust *R* genes *R*_*adv*_ and *R*_*11*_ and the male fertility restorer genes *Rf1*, *Rf5* and *Rf7*, and sub-cluster II including the rust *R* genes *R*_*4*_, *R*_*13a*_, *R*_*13b*_, and *R*_*16*_, and the four downy mildew *R* genes *Pl*_*5*_, *Pl*_*8*_, *Pl*_*21*_, and *Pl*_*34*_^8,16,22,47,48,51,55^. The two sub-clusters are approximately 23 Mb apart based on evidence from the two linked genes, *Rf7* and *Pl*_*34*_, originally from the wild *H. annuus* species, accession PI 413157. Both genes were mapped to an interval of 5.8 cM genetic distance on chromosome 13 and located in the two sub-clusters, respectively^8^. The two SNP markers, NSA_001167 closely linked to *Rf7* and SFW08875 closely linked to *Pl*_*34*_, located at the positions of 170,812,277 and 193,131,123 bp, respectively, in the XRQ genome, delimited the two genes to a physical interval of 22.3 Mb^8^. In the current study, sequencing-based chromosome walking combining with fine mapping delineated *Rf5* and *R*_*11*_ to regions of 30.6 and 2.1 kb in the XRQ genome within the sub-cluster I, and diagnostic SNP markers for *Rf5* and *R*_*11*_ were developed to facilitate marker-assisted breeding. Sequence alignment indicated that scaffold206293 from the HA-R9 sequence assembly shared 97% sequence identity with two candidate genes, Ha4121r1_13g048260 and HanXRQChr13g0420371, which codes a PPR protein, a motif of most cloned *Rf* genes, providing a starting point for *Rf5* gene cloning in the future (Table 4). Its predicted ORF was 1188 bp in length and incomplete, suggesting the first step for future work is to retrieve the surrounding sequences for a complete ORF.

**Figure 7:**
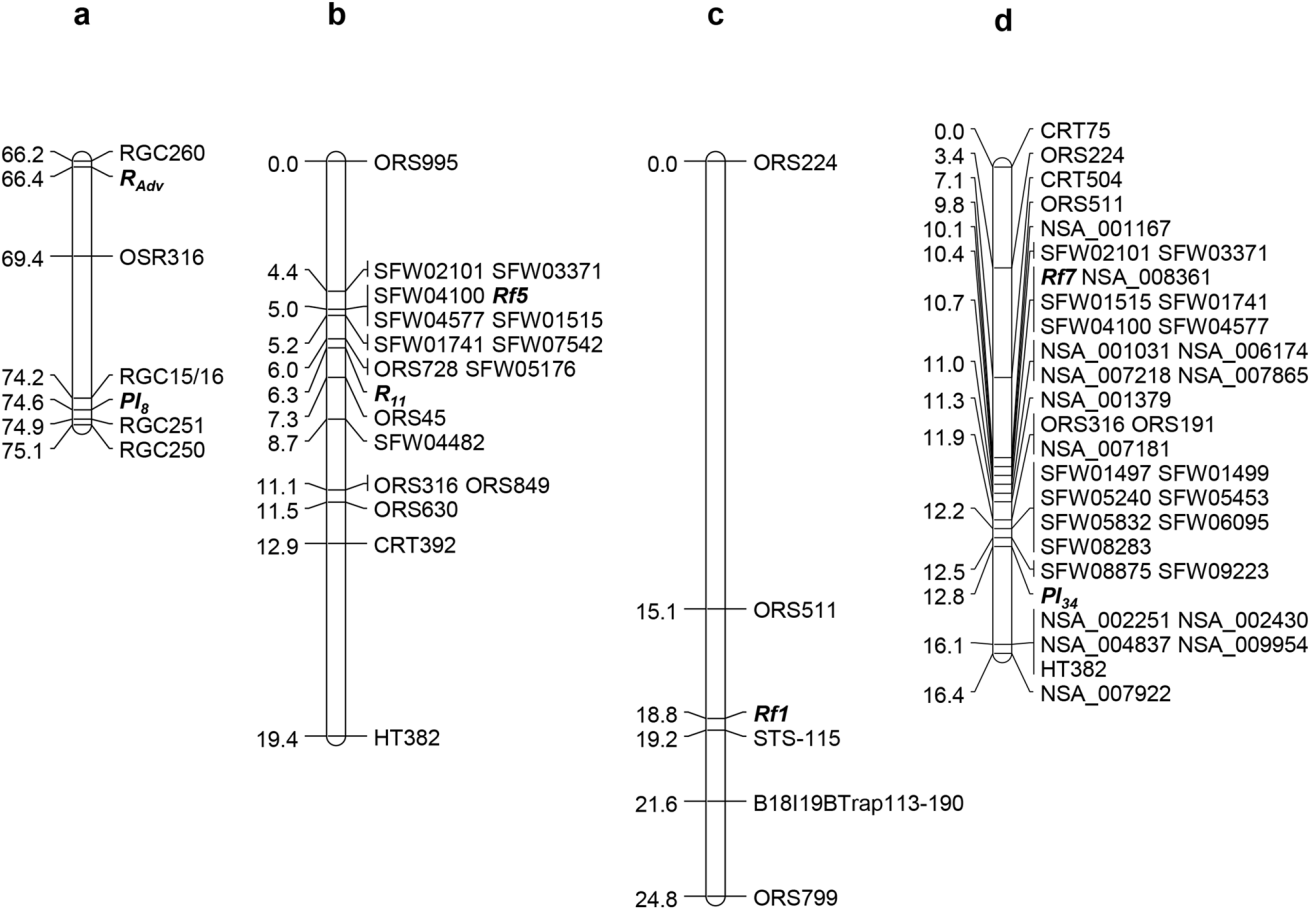
Comparison of the male fertility restoration genes and rust resistance genes mapped to sub-cluster I of chromosome 13 in different studies. (**a**) the positions of the *R*_*adv*_ gene^47^; (**b**) the positions of the *Rf5* and *R*_*11*_ genes (current study); (**c**) the position of the *Rf1* gene^16^; and (**d**) the position of *Rf7* gene^8^.

Clustering of *Rf* genes is common in plant species, having been reported in common bean, rice, and petunia^25,42,59^. The *Rf* gene cluster harboring five active genes (*Rf1a*, *Rf1b*, *Rf4*, *Rf5* and *Rf98*) located on rice chromosome 10 shows extreme variation in structure and gene content^37^. In sunflower, among seven *Rf* genes reported, three of them, *Rf1*, *Rf5*, and *Rf7*, were mapped to sub-cluster I in the lower end of chromosome 13^8,16,22^. Yue et al. (2010) localized *Rf1* at a position 3.7 cM proximal to SSR marker ORS511 on LG 13, equivalent to chromosome 13^16^. *Rf7* was mapped at a location 0.9 cM proximal to ORS511 in chromosome 13^8^, while *Rf5* shared a common SSR marker ORS316 with *Rf7* in the target region^22^, suggesting the close genetic relationship of *Rf1* and *Rf7*, as well as *Rf5* (Fig. 7b,c,d).

Three *Rf* genes, *Rf1*, *Rf5*, and *Rf7*, originated from the different accessions of the sunflower wild species *H. annuus* collected from Texas (*Rf1*), Oklahoma (*Rf5*), and New Mexico (*Rf7*), respectively^8,22,60^. Recently, a genome-wide association study identified 24 significant SNP markers associated with *Rf1*, which are located in a region where *Rf5* and *Rf7* reside^8^. Among 24 SNPs associated with *Rf1*, only five and seven SNPs retained the *Rf1* alleles in HA-R9 (*Rf5*) and RHA 428 (*Rf7*), respectively, suggesting that they are three different genes within a gene cluster. Owens et al. (2018) reported a candidate gene for *Rf1*, HanXRQChr13g0419821, which encodes an aldehyde dehydrogenase gene similar to a cloned *Rf2* gene reported in maize^23,24,61^. HanXRQChr13g0419821 is located at the 174,022,089 bp position of the XRQ genome within the region where 24 SNPs associated with *Rf1* reside. In the present study, fine mapping positioned *Rf5* in an interval of 175,223,522–175,254,164 bp on chromosome 13 in the XRQ genome. Additionally, one of the candidate genes for *Rf5*, HanXRQChr13g0420371, was found at the 175,253,986 bp position of the XRQ assembly (Table 4), while *Rf7* was reported in the interval 170,762,684–172,543,880 bp on chromosome 13 of the XRQ genome^8^ (Table 7). Taken together, we propose a hypothesis of three genes ordered within sub-cluster I: *Rf7* near the 172 Mb position, *Rf1* at 174 Mb, and *Rf5* at 175 Mb.

**Table 7:** Genetic and physical positions of markers linked to *Rf5* and *Rf7* on chromosome 13.

SFW map^a^	*Rf5* fine map^b^	*Rf7* map^c^	Marker	Physical position on XRQ assembly (bp)		Physical position on HA412-HO assembly (bp)	
cM	cM	cM		Start	End	Start	End
45.1	0.00000	10.4	SFW03371	169,537,912	169,538,031	220,170,730	220,170,837
45.1	0.00000	10.4	SFW02101	169,600,244	169,600,352	220,283,882	220,283,991
45.5	0.00128	10.7	SFW01515	170,762,684	170,762,803	214,069,367	214,069,487
45.5	0.00185	10.7	SFW04577	170,914,547	170,914,666	216,832,437	216,832,557
		10.7	***Rf7***				
45.5	0.00171	10.7	SFW04100	172,543,880	172,543,768	218,716,291	218,716,404
	0.00242		S13_216392091	175,223,922	175,223,522	216,391,891	216,392,291
	0.00242		***Rf5***				
	0.00256		C13_175253964	175,253,764	175,254,164	216,357,107	216,356,707
	0.00256		C13_175255719	175,255,919	175,255,519	216,355,352	216,354,952
	0.00256		C13_175260181	175,260,381	175,259,981	216,348,006	216,347,606
	0.00256		C13_175261496	175,261,296	175,261,696	216,346,692	216,346,292
	0.00256		C13_175273046	175,273,246	175,272,846	216,344,815	216,344,415
	0.00256		S13_216344422	175,273,439	175,273,039	216,344,222	216,344,622
	0.00284		C13_175253519	175,253,319	175,253,719	216,357,552	216,357,152
	0.00327		C13_175276537	175,276,337	175,276,737	222,305,963	222,306,363
	0.00483		SFW07542	174,907,936	174,907,818	216,954,392	216,954,277

^a^Markers taken from Bowers et al. (2012)^67^, ^b^markers taken from present study, and ^c^markers taken from Talukder et al. (2019)^8^.

Sub-cluster I with three the *Rf* genes also harbors two rust *R* genes, *R*_*adv*_ and *R*_*11*_ linked to *Rf5*, which are positioned distal to a common SSR marker ORS316 at the genetic distances of 3.0 and 3.7 cM in the two maps, respectively^22,47^ (Fig. 7). *R*_*adv*_ originated from a sunflower wild species *H. argophyllus* and encodes specific recognition to rust infection, different from that of *R*_*11*_^51^. Bachlava et al. (2011) reported that an NBS-LRR-encoding resistance gene candidate (RGC) marker RGC260 most closely linked to *R*_*adv*_ was mapped to 0.2 cM distal to the *R*_*adv*_ locus^47^ (Fig. 7a). Alignment of the RGC260 reverse primer sequence to the XRQ genome sequence indicated that RGC260 is located at the position of 178,056,184 bp in the XRQ genome assembly. In the present study, fine mapping delimited *R*_*11*_ to an interval between 181,789,941 and 181,792,357 bp in the XRQ genome, indicating that *R*_*adv*_ and *R*_*11*_ are two closely linked, but different genes.

Although the success of *Rf* gene cloning has been reported in maize, peanut, radish, rice, sorghum, and sugar beet, its cloning from sunflower is precluded due to the large genome size and high proportion of repetitive sequences^23,25,26,32,35,38,39,62^. Sunflower is a diploid species with a genome size of approximately 3.6 Gb and more than 80% repetitive sequences. The availability of sunflower genome sequences of two inbred lines, HA412-HO and XRQ, has enabled the development of high density molecular markers and accelerated fine mapping and map or sequence-based gene cloning^57^. With reference-guided chromosome walking, we identified three and two candidate genes for *Rf5* from the HA412-HO and XRQ assemblies, respectively. Among them, two from the HA412-HO assembly, Ha412v1r1_13g048240 and Ha412v1r1_13g048260, both had PPR motifs typical of *Rf* genes, and one from XRQ assembly, HanXRQChr13g0420371, showed a typical tetratricopeptide-like helical domain, which shares 100% sequence identity with Ha412v1r1_13g048260, indicating the sequence of Ha412v1r1_13g048260 is highly conserved in sunflower, at least among the sunflower lines we studied.

The majority of cloned *Rf* genes in plants encode a specific clade of the RNA-binding PPR protein family^42,63^. Duplicated PPR-containing genes residing within the *Rf* locus are habitual in plant species. A pair of duplicated PPR-containing genes, *Rf-PPR591* and *Rf-PPR592*, was found to reside in the *Rf* locus in *Petunia*, share 93% sequence similarity and are identical in PPR organization, but only differ in the last 12 C-terminal amino acids^25^. Further functional characterization confirmed *Rf-PPR592* was able to restore fertility to CMS plants, but not *Rf-PPR591*, suggesting *Rf-PPR592* as the *Rf* gene in *Petunia*^25^. In the current study, the two candidate genes, Ha412v1r1_13g048240 and Ha412v1r1_13g048260, were located within a narrow 12.1 kb region at positions 216,342,878 and 216,354,988 bp in the HA412-HO assembly, respectively (Table 3). Both genes encode a PPR protein and share 92% sequence similarity, suggesting it is likely one of the two is the candidate *Rf5* gene in sunflower.

As HA412-HO does not have the *Rf5* gene, the candidate genes in HA412-HO could be *rf* or pseudo-alleles and fail to interact with cytoplasm for fertility restoration. Thus, it is important to determine the physical location of *Rf5* in the HA-R9 genome. The contigs and scaffolds from HA-R9 whole-genome resequencing and assembly were searched with the use of the candidate gene sequences as queries, and contig C16613275 and scaffold206293 were determined to share very high sequence identity (97–99%) with queries. However, the predicted ORF of scaffold206293 is incomplete, and a large portion of it consists of undetermined nucleotides, which is not uncommon of the repetitive sequences in the sunflower genome and the sequencing system. All these targeted contigs and scaffolds feature a typical PPR motif with a series of degenerate 35-amino-acid repeats of different numbers (Table 5). Due to the unavailability of a stable transformation system in sunflower, we were unable to confirm their function to restore male sterility. Alternatively, we developed an EMS mutant population of HA-R9, and sterile male plants, resulting from mutations in the *Rf5* locus in the M1 generation, were obtained in the M3 families. Target region sequencing of the *Rf5* mutant plants and the *Rf5* donor HA-R9 is underway using PacBio long-read sequencing for further functional analysis.

HA-R9 carrying *Rf5* has been tested for its male fertility restoration to eight different CMS lines, including PET1, PET2, MAX1, GIG1, ANN2, ANN3, RIGX, and GIG2^56^. The results indicated that *Rf5* can only restore PET1 CMS, just as *Rf1*. *Rf5* is approximately 6 Mb apart from *R*_*11*_ with a recombination ratio of 1.3% between the two genes (Fig. 1b). Therefore, *Rf5* and *R*_*11*_ could be used as a linkage block in sunflower breeding programs. The introgression of these two genes into new hybrids is important, as *Rf5* provides a new *Rf* gene to PET1 CMS, and *R*_*11*_ provides resistance to all *P. helianthi* races identified so far in North America. The high-density map and diagnostic SNP markers developed provide the information and tools required to accelerate the transfer of *Rf5* and *R*_*11*_ into elite sunflower lines.

## Methods

### Plant materials

An F_2_ population with 192 individuals previously used to map *Rf5* and *R*_*11*_ with SSR markers was used for saturation mapping in the current study^22^. In its original cross, the inbred line HA 89 was susceptible to rust, while the wild *H. annuus* accession PI 613748 was used as a donor of *Rf5* and *R*_*11*_. A sunflower germplasm line, HA-R9, characterized as homozygous for both *Rf5* and *R*_*11*_, released by USDA and North Dakota State University in 2013, was used as a gene donor for whole-genome resequencing^56^.

For fine mapping of *Rf5* and *R*_*11*_, recombinants were screened from 3517 and 8795 F_3_ individuals selected from the previously characterized F_2:3_ families heterozygous for *Rf5* or *R*_*11*_, respectively. Each selected heterozygous F_3_ family was considered a segregating F_2_ population for *Rf5* or *R*_*11*_.

An evaluation panel was assembled consisting of 96 inbred sunflower lines with diverse origins, including 20 and 17 lines known to harbor the different male fertility restoration *Rf* genes and the rust *R* genes, respectively (Supplementary Table S1). This panel was used to validate the diagnostic DNA markers linked to *Rf5* and *R*_*11*_.

### Male fertility evaluation

F_2:3_ individuals and F_2:4_ families were visually scored as fertile or sterile based on the presence or absence of pollen. Plants that could develop anthers and shed pollen were considered fertile, while those that could not develop anthers or pollen were considered sterile. Recombinants selected from the fertility restoration segregating F_2:3_ population were grown in the greenhouse to evaluate male fertility. From 3517 F_2:3_ individuals, 86 were selected as recombinant between the markers SFW03371 and ORS728; of these recombinants, 23 were male-sterile, and 63 were fertile. The subsequent generation of 61 fertile F_2:4_ recombinant families (35 seeds each) were grown in a field at Glyndon, MN, in the summer of 2015 to evaluate their homozygosity and heterozygosity. The family was considered homozygous fertile if all the plants in the family were able to develop anthers and shed pollen. Conversely, the family was considered heterozygous fertile if the plants in the family were segregating for male fertility.

### Rust resistance evaluation

The recombinants selected from the fine mapping population were evaluated for rust resistance in the greenhouse in 2015. Twenty seeds from each of the selected F_2:4_ recombinant families, together with the parents HA 89 and HA-R9, were grown in 4 × 9 cell plastic flats filled with Sunshine SB 100B potting mixture (SunGro Horticulture, Bellevue, WA, USA). Regular greenhouse maintenance was performed until seedlings reached the four-leaf stage. The *P. helianthi* isolate of race 336 was chosen for testing seedlings using an artificial inoculation procedure described by Qi et al. (2011)^64^. Leaves were inoculated with urediniospores of *P. helianthi* race 336. Resistance against rust was evaluated 12 to 14 days after inoculation for both infection types (ITs) based on the 0 to 4 scale described by Yang et al. (1986)^65^ and the percentage of leaf area covered with pustules (severity) described by Friskop et al. (2015)^66^. Infection types 0, 1, and 2 combined with pustule coverage of 0 to 0.5% were classified as resistant, and ITs 3 and 4 with pustule coverage > 0.5% were considered susceptible.

### Saturation mapping, whole-genome resequencing, and SNP marker identification

For saturation mapping, a total of 45 SNP markers potentially mapped around *Rf5* and *R*_*11*_ gene loci on chromosome 13 were selected after comparison with a published genetic map^67^ (hereafter referred to as SFW-SNPs, Supplementary Table S2).

HA-R9 was sequenced on the Illumina HiSeq/MiSeq sequencing platform at Novogene Corp. according to their protocols. Briefly, quality DNA samples were randomly fragmented using Covaris cracker to 350 bp in size for library construction, and later qualified libraries were pooled for sequencing according to effective concentrations and expected data volume. Raw reads resulting from next generation sequencing were trimmed and filtered to remove adapters, reads with > 10% undetermined bases, and reads with more than half of the bases of low quality (Q_score_ ≤ 5). After filtering, clean reads were separately mapped to the two publicly available sunflower reference genomes, HA412-HO (https://www.heliagene.org/HA412.v1.1.bronze.20141015/) and XRQ (https://www.heliagene.org/HanXRQ-SUNRISE/). All SNPs and InDels were identified using the genome-mapped reads. The SNP markers were named with prefix C13 or S13 followed by a number representing the physical position of each SNP along chromosome 13 of each reference genome assembly (hereafter referred to as WGS-SNPs, Supplementary Table S3a and S3b). The prefix C13 represents the SNP from the XRQ reference genome assembly, while S13 represents the SNP from the HA412-HO reference genome assembly.

### Genotyping of PCR-based markers

SSR marker genotyping was performed as described by Qi et al. (2012)^49^. Genotyping of polymerase chain reaction (PCR)-based SNP markers was conducted as described by Qi et al. (2015)^68^ and Long et al. (2017)^69^. For each SNP, two-tailed forward allele-specific primers (AS-primers F1 and F2) and one common reverse primer were designed (Supplementary Table S4). A universal priming-element-adjustable primer (PEA-primer 5′-ATAGCTGG-Sp9-GCAACAGGAACCAGCTATGAC-3′) with an attached fluorescence tag at the 5′ terminus was used in each PCR. The PCR protocol for SNP genotyping was conducted as described by Ma et al. (2017)^70^. Upon amplification, PCR products were loaded on a 6.5% polyacrylamide gel for visualization using an IR2 4300/4200 DNA analyzer (LI-COR, Lincoln, NE, USA).

### Sequence assembly, alignment and candidate gene identification

The clean paired-end reads from HA-R9 whole-genome resequencing were assembled using SOAPdenovo2 and gaps were repaired^58^. The two genomic regions, 216,334,932–216,393,092 bp and 225,224,729–225,301,092 bp on chromosome 13 from the HA412-HO genome sequence assembly were selected to identify contigs and scaffolds possibly having *Rf5* and *R*_*11*_ genes, respectively. The sequences of the selected contigs and scaffolds are presented in Supplementary Table S5. A standalone BLASTN program downloaded from the NCBI (ftp://ftp.ncbi.nlm.nih.gov/blast/executables/blast+/LATEST/) was used to conduct a BLAST search of the reference sequences of the two genomic regions mentioned above against the assembled HA-R9 contigs and scaffolds, at E-value e-20. Selected contigs and scaffolds showing sequence similarity were again aligned with candidate genes identified in the reference assemblies using the BLASTN suite (https://blast.ncbi.nlm.nih.gov/Blast.cgi). Open reading frames (ORF) were identified among the selected contigs and scaffolds using ORFfinder (https://www.ncbi.nlm.nih.gov/orffinder/). Multiple sequence alignments of deduced amino acid sequences of the *Rf5* candidate gene Ha412v1r1_13g048260 with the contigs and scaffolds from HA-R9 and the characterized *Rf* orthologues from different plant species were performed using MultAlin version 5.4.1 (http://multalin.toulouse.inra.fr/multalin/).

### Ethical standards

The experiments were performed in compliance with the current laws of the USA.

## Supplementary Information

Supplementary Information.
