# Supplementary material for: Map and sequence-based chromosome walking towards cloning of the male fertility restoration gene Rf5 linked to R11 in sunflower
Source: Sci Rep. 2021 Jan 12;11:777. doi: 10.1038/s41598-020-80659-6 (PMC7804242; doi:10.1038/s41598-020-80659-6)
Supplement: Supplementary file 1 — Supplementary Information. [file 41598_2020_80659_MOESM1_ESM.pdf]

Map and sequence-based chromosome walking towards cloning of the male fertility restoration gene *Rf5* linked to R_{11} in sunflower

Guojia Ma[1], Yunming Long[1], Qijian Song[2], Zahirul I. Talukder[1], Md Shamimuzzaman[3], Lili Qi[3]*

[1]Department of Plant Sciences, North Dakota State University, Fargo, ND, 58108 USA
[2]USDA-Agricultural Research Service, Soybean Genomics and Improvement Laboratory, 10300 Baltimore Ave., Beltsville, MD 20705, USA
[3]USDA-Agricultural Research Service, Edward T. Schafer Agricultural Research Center, 1616 Albrecht Blvd. N, Fargo, ND 58102-2765 USA

*Corresponding author (lili.qi@usda.gov)

Map and sequence-based chromosome walking towards cloning of the male fertility restoration gene Rf5 linked to R_{11} in sunflower

Guojia Ma[1], Yunming Long[1], Qijian Song[2], Zahirul I. Talukder[1], Md Shamimuzzaman[3], Lili Qi[3]*

Supplementary Figure S1. Multiple sequence alignment of deduced amino acid sequence of the candidate gene Ha412v1r1_13g048260 with characterized *Rf* orthologues from petunia, radish, rice and sorghum. Sequence identifiers started with Ha, Ph, Rs, Os and Sb denotes for *Helianthus annuus*, *Petunia x hybrid*, *Raphanus sativus*, *Oryza sativa* and *Sorghum bicolor*.

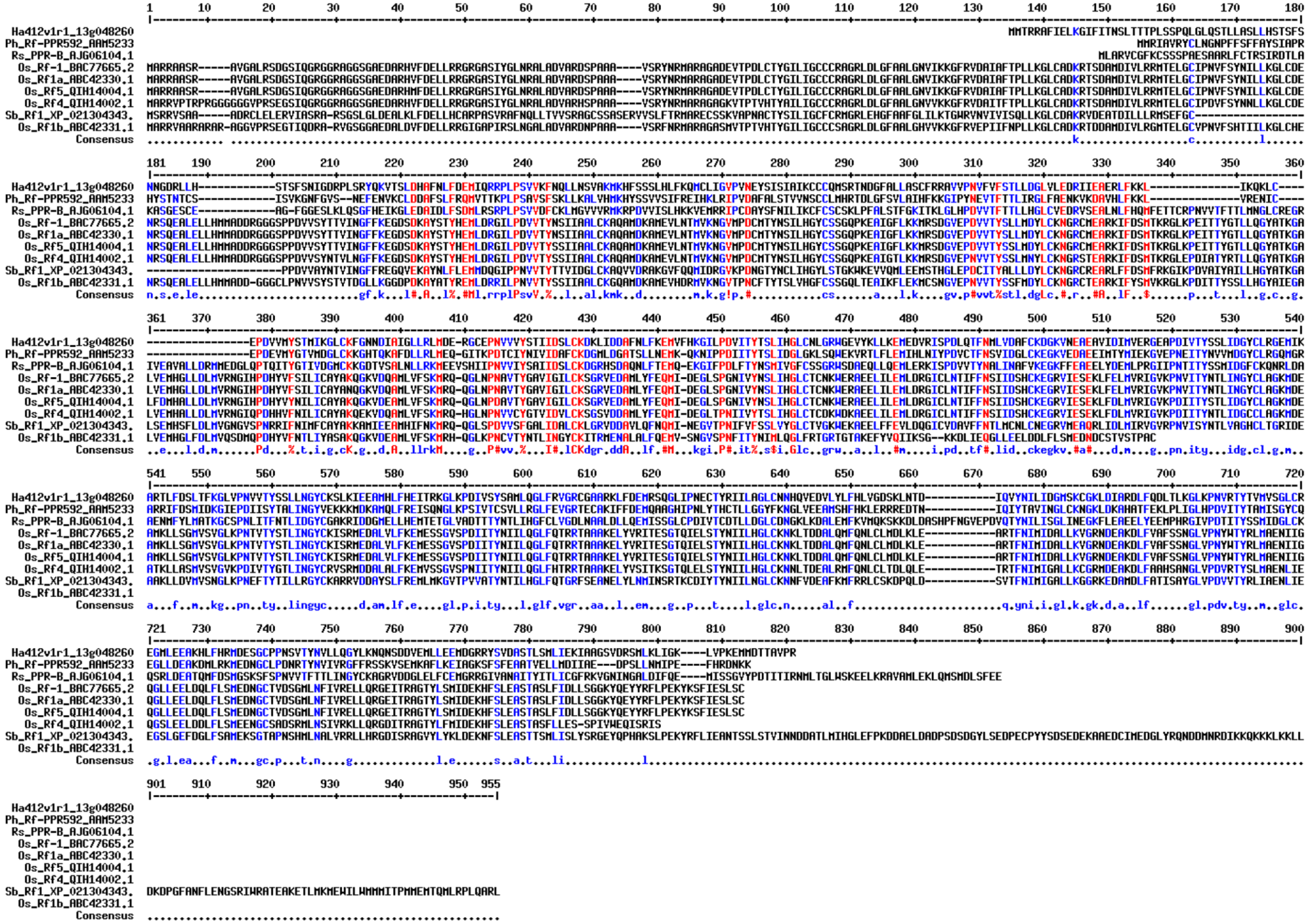

Map and sequence-based chromosome walking towards cloning of the male fertility restoration gene Rf5 linked to R_{11} in sunflower

Guojia Ma[1], Yunming Long[1], Qijian Song[2], Zahirul I. Talukder[1], Md Shamimuzzaman[3], Lili Qi[3]*

Supplementary Figure S2. Original gel image of SNP marker C13_175260181 diagnostic for *Rf5* on a panel of 96 diversified sunflower lines, lanes 1-48.

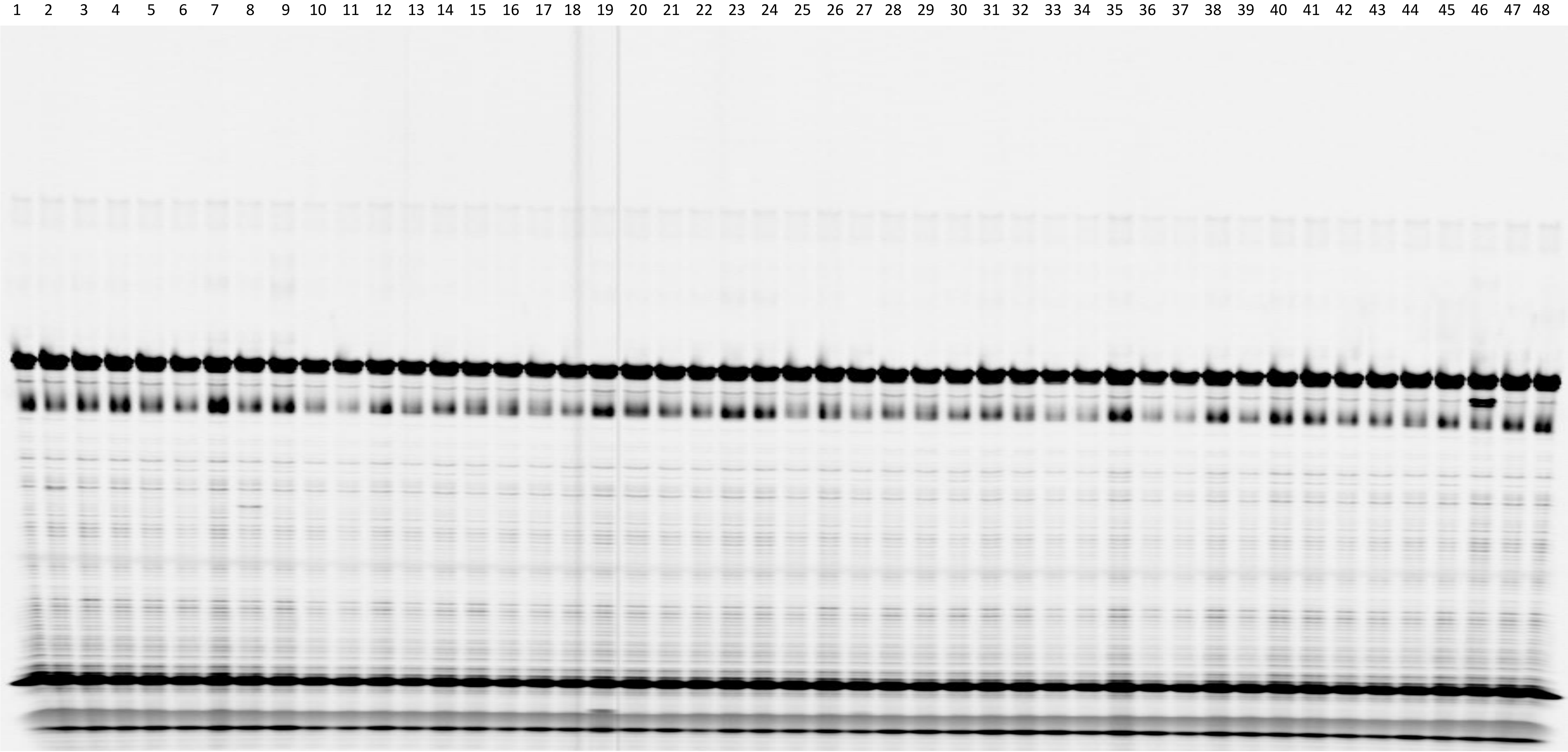

Map and sequence-based chromosome walking towards cloning of the male fertility restoration gene Rf5 linked to R_{11} in sunflower

Guojia Ma[1], Yunming Long[1], Qijian Song[2], Zahirul I. Talukder[1], Md Shamimuzzaman[3], Lili Qi[3]*

Supplementary Figure S2. Original gel image of SNP marker C13_175260181 diagnostic for *Rf5* on a panel of 96 diversified sunflower lines, lanes 49-96.

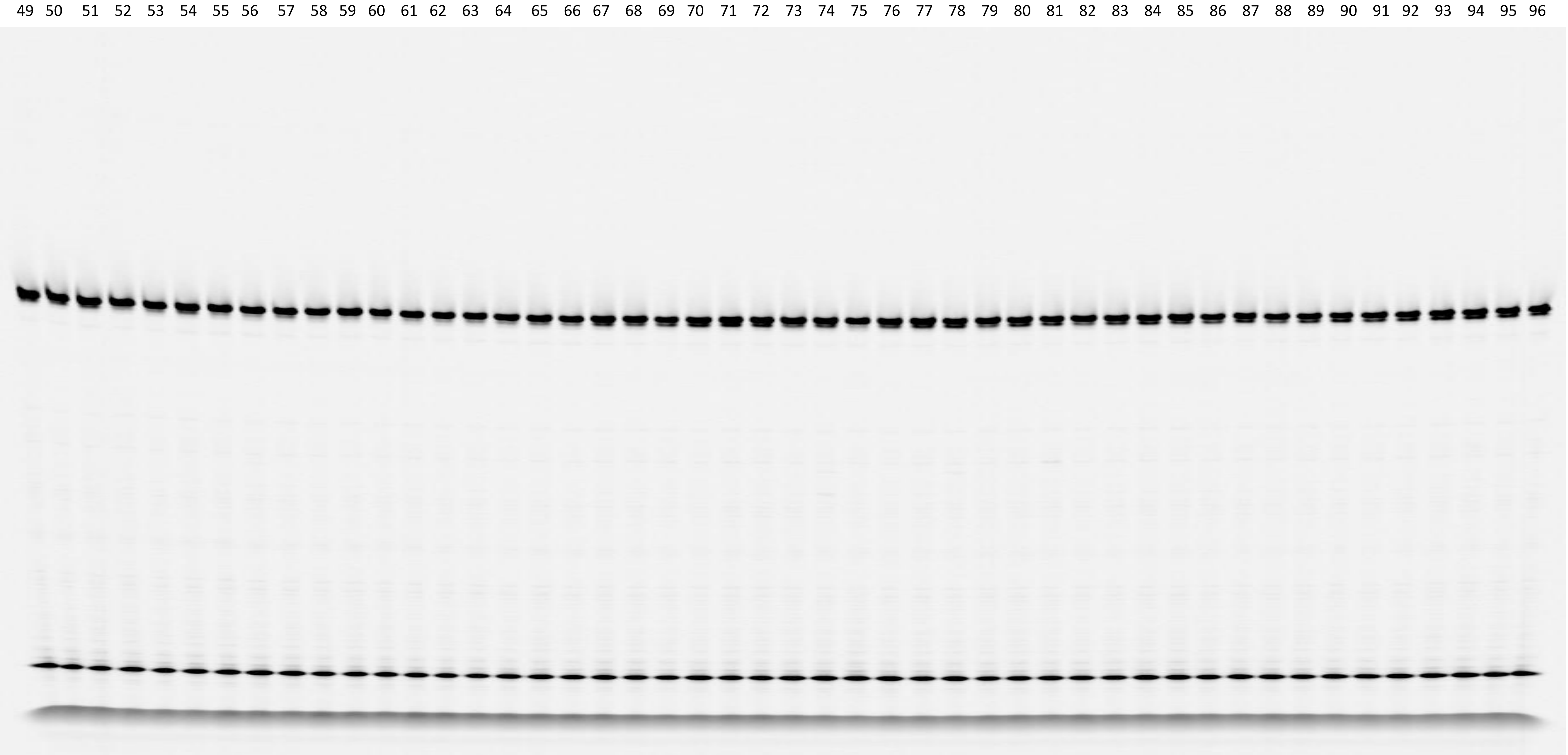

Map and sequence-based chromosome walking towards cloning of the male fertility restoration gene Rf5 linked to R_{11} in sunflower

Guojia Ma[1], Yunming Long[1], Qijian Song[2], Zahirul I. Talukder[1], Md Shamimuzzaman[3], Lili Qi[3]*

Supplementary Figure S3. Original gel image of SNP marker C13_181792517 diagnostic for R_{11} on a panel of 96 diversified sunflower lines, lanes 1-48.

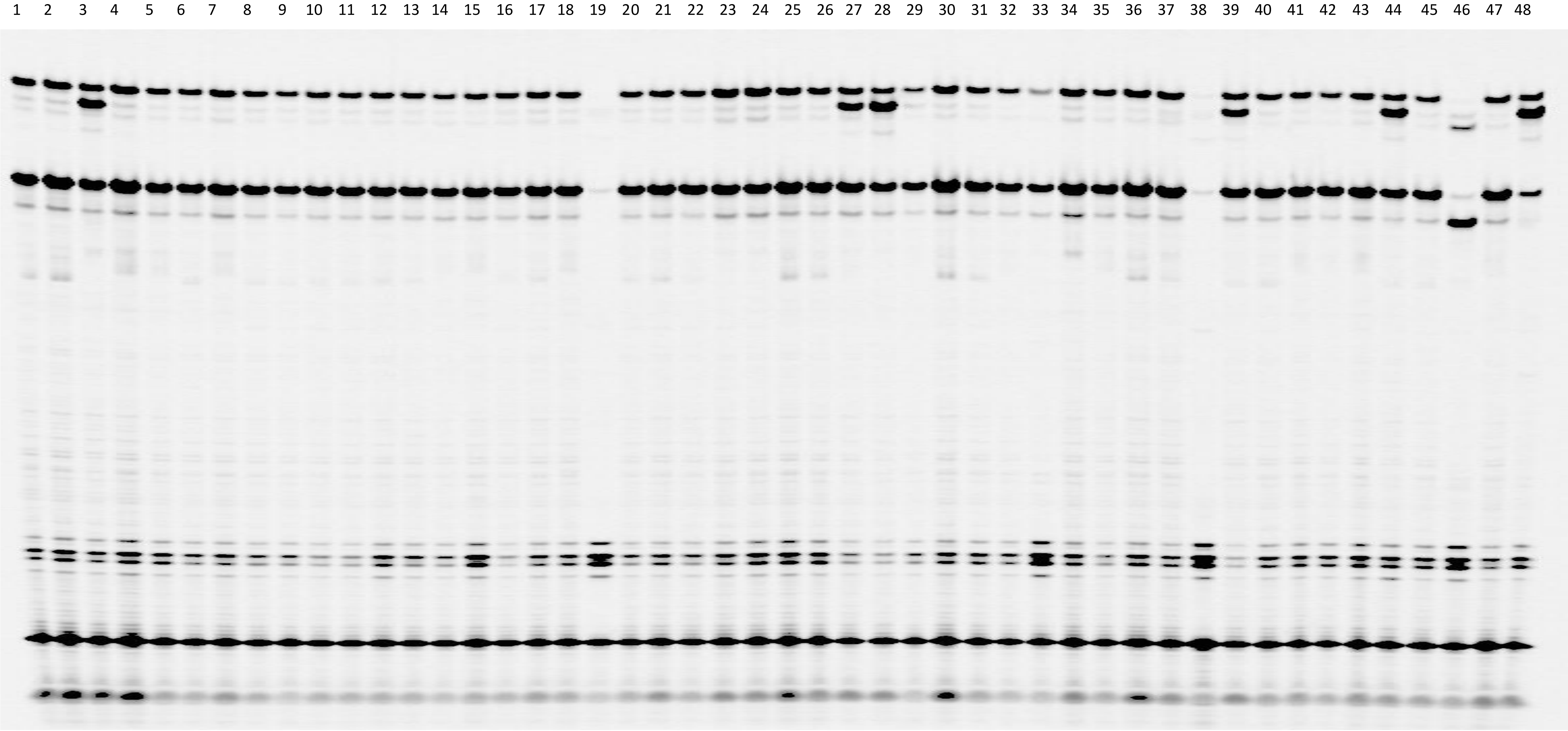

Map and sequence-based chromosome walking towards cloning of the male fertility restoration gene Rf5 linked to R_{11} in sunflower

Guojia Ma[1], Yunming Long[1], Qijian Song[2], Zahirul I. Talukder[1], Md Shamimuzzaman[3], Lili Qi[3]*

Supplementary Figure S3. Original gel image of SNP marker C13_181792517 diagnostic for R_{11} on a panel of 96 diversified sunflower lines, lanes 49-96.

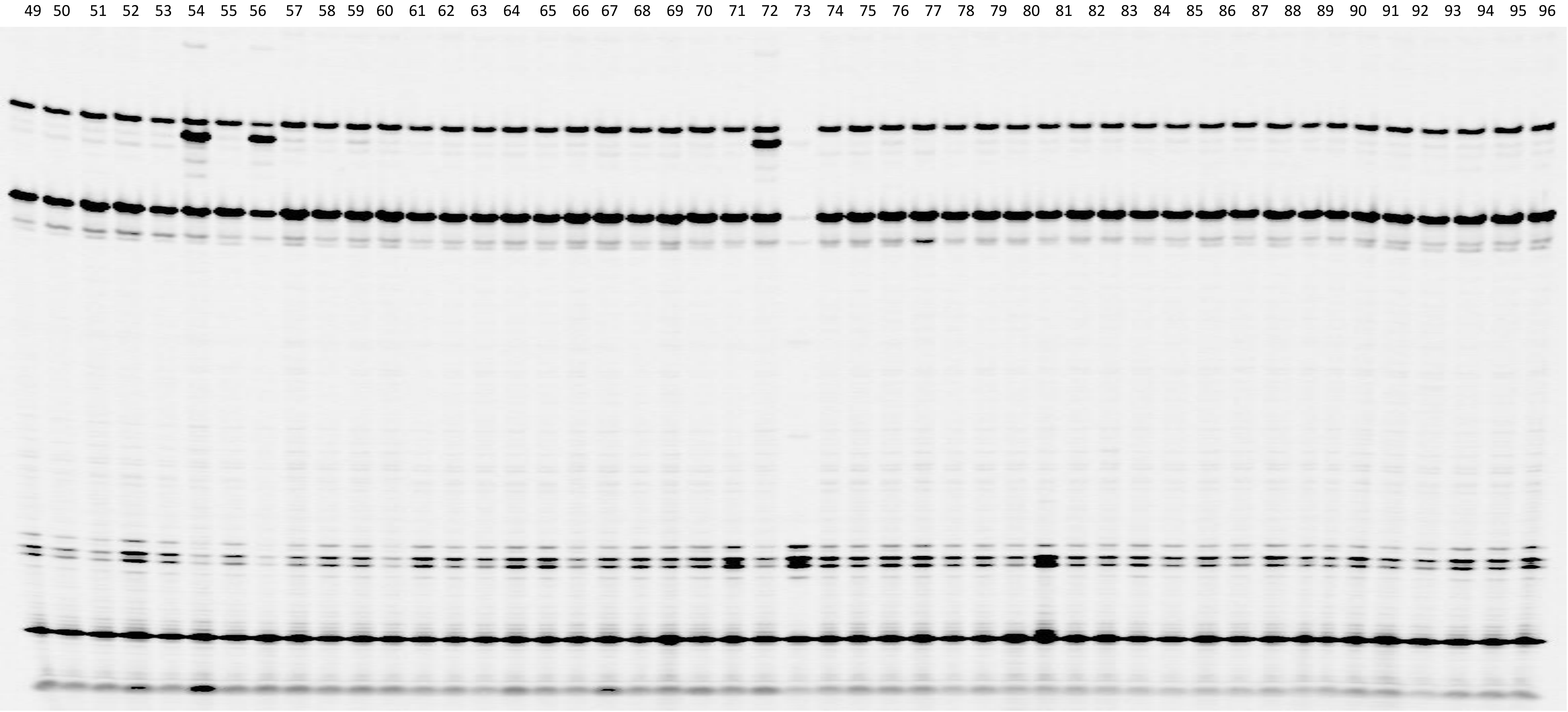

Map and sequence-based chromosome walking towards cloning of the male fertility restoration gene *Rf5* linked to R_{11} in sunflower

Guojia Ma[1], Yunming Long[1], Qijian Song[2], Zahirul I. Talukder[1], Md Shamimuzzaman[3], Lili Qi[3]*

Supplementary Table S1 Evaluation panel of 96 sunflower lines used to determine specificity of *Rf5* and R_{11} markers

Pl: downy mildew R gene; R: rust R gene; Rf: male fertility restoration gene; BSR: Sclerotinia basal stalk rot

Lane No. in PCR gel	Sunflower line	Gene	Pedigree	Release date
1	HA 89		VNIIMK 8931 Sel	October 1971
2	HA 234		2*Smena//HA6/HA8	October 1971
3	HA 291		INRA 6501 Sel	April 1976
4	HA 321		HA 287/HA 286	April 1985
5	HA 323		Sundak Selection	April 1985
6	HA 342		HA 89 *2/Pervenets High Oleic	April 1986
7	HA 350		HA 292*2/Pervenets High Oleic	April 1986
8	HA 378		HA 821/DDR	May 1990
9	HA 412		USDAB/SCL B-3 CYCLE 1	May 1995
10	HA 412 HO		HA 412*5/HA 434	May 1995
11	HA 434		HA424 High Oleic selection	April 2001
12	HA 441		HA412/SD Sclerotinia Tolerant	May 2003
13	HA 850		High Oil Population	April 1985
14	CONFSCLB1		Maintainer selection from HA 441/ROM PH//HA 442/HA 441/4/CONF/5/CONF	2006
15	CONFSCLR5		Restorer selection from RO12-13//RHA 274/Dobritch/3/PSC 8/4/CONF/5/CONF	2006
16	RHA 265	Pl_1	2*Peredovik/953-102-1-1-41 = T66006-2-1-3-1	1971
17	RHA 274	Pl_2/*Rf1*	CMS PI343765/HA119//HA62-4-5 /2/T66006-2	May 1973
18	HA 61	Pl_3	953-88-3/Armavirski 3497	1968
19	DM-2	Pl_5	Composite - Source = Novinka	February 1984
20	HA 335	Pl_6	HA 89 *3/H. annuus 423	1986
21	HA 452	Pl_6	HA335/HA412	May 2005
22	HA 337	Pl_7	HA 89 *3/H. praecox 417	1986
23	RHA 340	Pl_8/R_{adv}/*Rf3*	HA 89 *3/H. argophyllus 415	April 1986
24	RHA 436	Pl_8	RHA340 / RHA344 High Oleic	April 2001
25	RHA 419	Pl_{Arg}/*Rf1*	RHA 373 / ARG 1575-2	May 1999
26	RHA 468	Pl_{Arg}/*Rf1*	RHA 428/3/RHA 426/RHA 419//RHA 440/4/RHA 426/3/RO 12-13//RHA 274/Dobritch	May 2006
27	HA-R5	Pl_{13}	Guayacan INTA Selection	January 1984
28	HA-R4	Pl_{14}/Pl_{16}	Saenz Pena 74-1-2 Selection	January 1984
29	RNID	Pl_{15}	Proprietary restorer inbred line which traces back to an Argentine sunflower open pollinated population	-
30	HA 458	Pl_{17}	HA434*3/PI468435	May 2006
31	HA-DM1	Pl_{18}	HA 89*2/NMSHA89/PI 494573	June 2015

32	HA-DM2	Pl_{Arg}/R_{12}	BC4F4 confection restorer selection from CONFSCLR5*5/RHA 464	June 2017
33	HA-DM3	Pl_{17}/R_{13a}	BC4F4 confection maintainer selection from HA HA-R6*5/HA 458	June 2017
34	HA-DM4	Pl_{18}/R_{13a}	BC4F3 confection maintainer selection from HA R6*5/HA-DM1	June 2017
35	HA-DM5	Pl_{19}	BC2F4 confection maintainer selection from CONFSCLB1*2/CMS CONFSCLB1/H. annuus PI 435414	June 2017
36	16-039-48	Pl_{20}	Homozygous BC2F2 of HA89/H.argophyllus PI494578	-
37	RHA 428	Pl_{34}	RHA 801 // RHA 365 / PI 413157	June 2000
38	803-1	Pl_{803}	Former Yugoslavia selection	-
39	TX16R	Pl_{33}/R_{16}	TX16/HA 89//TX16/HA 89	2005
40	MC 90	R_1	-	-
41	MC 29 (Australia)	R_2	Australian selection of oil-type Canadian line MC29	-
42	MC 29 (USDA)	R_2/R_{10}	USDA selection of oil-type Canadian line MC29	-
43	PhRR3	R_3	Backcross between S37-388 and Hysun 33s	-
44	HA-R3	R_4	Charata Selection	January 1984
45	HA-R2	R_5	Impira INTA Selection	January 1984
46	HA-R9	$R_{11}/Rf5$	Improved derivative of RfANN-1742	January 2013
47	HA-R10	R_5	Maintainer line from CONFSCLB1*4/HA-R2	December 2013
48	HA-R11	R_4	Restorer line from CONFSCLR5*5/HA-R3	December 2013
49	RHA 464	$R_{12}/Pl_{Arg}/Rf1$	RHA418/RHA 419/3/ RHA801//RHA365/PI 413047	May 2006
50	HA-R6	R_{13a}	Confection mainatiner line PI 607509	2001
51	HA-R12	R_2/R_{13a}	Maintainer selection from CONFSCLB1*5/MC29 (AUS) and HA-R6	Junly 2014
52	HA-R13	R_5/R_{13a}	Maintainer selection from CONFSCLB1*4/HA-R2 and HA-R6	Junly 2014
53	RHA 397	R_{13b}	Male fertility restorer line of oilseed sunflower from RHA 274/RO-20-10-3-3-2	May 1993
54	PH3	R_{14}	BC2F4-derived line from the cross and backcrosses of HA 89/South Dakota wild H. annuus L. (PI 413038)	1993
55	HA-R8	R_{15}	RHA 377/PI 432512	May 1997
56	P-386	P_{u6}	Selection from Charata INTA	1986
57	RHA 282	*Rf1*	Boneta Giant Manchurian/Mennonite RR	March 1974
58	RHA 293	*Rf1*	3 * Commander /Mennonite RR	April 1976
59	RHA 299	*Rf1*	CMS P-21 VRI/T70050 T70050=PI343765/HA119 //HA62-4-5/2/T66008-2	November 1976
60	RHA 325	*Rf1*	R811-3	April 1985
61	RHA 348	*Rf1*	RHA 274 *2/Pervenets High Oleic	April 1986
62	RHA 374	*Rf1*	ARG-R43	May 1990
63	RHA 386	*Rf1*	82 ROM. R-LINE BULK	May 1992
64	RHA 400	*Rf1*	AUSTRALIA 85 R-LINE POP.	May 1995
65	RHA 408	*Rf1*	ROMANIA R-LINE SCL POP-1	May 1995
66	RHA 417	*Rf1*	RHA 801/NS-RF POP 3 (SUNBURST)	May 1998
67	RHA 439	*Rf1*	RHA377/AS3211 Sclerotinia Tolerant	May 2003

68	RHA 801	*Rf1*	Derived from a Restorer Composite	May 1980
69	RHA 280	*Rf3*	Sundak Sel	March 1974
70	Rf GIG2**	*Rf4*	cmsGIG2/H.maximiliani amphiploid	December 2016
71	RHA 295		CMS Mennonite RR- 18-1*3 / T66006-2	November 1976
72	RHA 364		FELIX	May 1988
73	RHA 366		RF-8784/RHA 274	May 1988
74	RHA 368		NS-H-27/RHA 274	May 1988
75	RHA 392		SELECT	May 1993
76	RHA 398		RHA 274/BCD LINE BULK	May 1995
77	RHA 426		RHA 409 / RHA 376*2 / H.annuus IMI resistant	June 2000
78	RHA 443		RHA426/RHA419//RHA440 Imidazolinone Herbicide Resistant	May 2003
79	RHA 447		RHA377/RHA348 High Oleic	May 2003
80	RHA 454		RHA 447//RHA440/PSC 8 (High ol.)	May 2005
81	RHA 472		Restorer oilseed line from RHA 801/AS 4379//RHA 439	August 2011
82	RHA 475		Restorer oilseed line from RHA 801/AS 4379//RHA 426	August 2011
83	RHA 478		RHA 443/RHA 455	December 2016
84	RHA 479		RO 12-13//RHA 274/DOB/3/PSC8/4IRHA418/RHA 419/3/R012-13/IRHA 2741PRS5	December 2016
85	RHA 855		CMS HA 89/RHA 273	April 1985
86	RHA 856		RHA 299/Sorem HT 58	April 1985
87	HA-BSR1	BSR resistance	F_7–derived RIL line from HA 441/RHA 439	August 2016
88	HA-BSR2	BSR resistance+Pl_{17}	BC_2F_6 maintainer oilseed sunflower line from HA 89/3/HA 458//NMSHA 89/PI 435843	October 2017
89	HA-BSR3	BSR resistance+Pl_{17}	BC_2F_6 maintainer oilseed sunflower lines from HA 89/3/HA 458//NMSHA 89/PI 494573	October 2017
90	HA-BSR4	BSR resistance+Pl_{17}	BC_2F_6 maintainer oilseed sunflower lines from HA 89/3/HA 458//NMSHA 89/PI 494573	October 2017
91	HA-BSR5	BSR resistance	BC_2F_6 maintainer oilseed sunflower lines from HA 89/3/HA 458//NMSHA 89/PI 494573	October 2017
92	HA-BSR6	BSR resistance+Pl_{17}	BC_2F_6 maintainer oilseed sunflower lines from HA 89/3/HA 458//NMSHA 89/PI 468853	October 2017
93	HA-BSR7	BSR resistance+Pl_{17}	BC_2F_6 maintainer oilseed sunflower lines from HA 89/3/HA 458//NMSHA 89/PI 468853	October 2017
94	HA-BSR8	BSR resistance+Pl_{17}	BC_2F_6 maintainer oilseed sunflower lines from HA 89/3/HA 458//NMSHA 89/PI 468853	October 2017
95	HOLS 1		BC_3F_4 maintainer genetic stock from HA 466/4/HA 466/3/RS I/HA 466//HA 466	June 2015
96	HOLS 4		F_8 maintainer genetic stock from HA 466/PI 170414 + PI372259 bulk	June 2015

Map and sequence-based chromosome walking towards cloning of the male fertility restoration gene *Rf5* linked to R_{11} in sunflower

Guojia Ma[1], Yunming Long[1], Qijian Song[2], Zahirul I. Talukder[1], Md Shamimuzzaman[3], Lili Qi[3]*

Supplementary Table S2

Map position and sequences of SNP markers on LG13 selected from published sunflower linkage map used for map saturation in the present study

SNP ID	Map position (cM)[a]	Target sequence
SFW02101	45.13	ATCCAAGGTCCCNAAAAGCTTCCTATTGTCGATTATCGGTGAAGATCGTGTTACGAAATT[T/C]GTCATTCAAGAAATAGTTAGCTCCACAATGGCTGATTATGTGAAGAAGAACAATATTCC
SFW03371	45.13	ACTTGCTGGTAAAGATTCAACTTCAACTGCACTCACATGGTTCTTTTGGCTATTAGACGG[A/G]CACCCGCATTGTAAACACATGATCCACAAAGAAATATCTATGTTAATGACCTCAACCCA
SFW01515	45.45	ATAAACTCCTAGATATTAAAAACACGNTTCGTTCATGTTATTAACCGTCCACTAGTCGCC[A/G]ACTTTTTCCGACTAACTTTGAAACACAATGAGCAGATTGAATTTGTAGTTTGATCTATT
SFW04100	45.51	AACGGTTATACCAATGCTTGAAGCTGTCATGGAAGTTGTGCCTGTTGAAAAGCTTGCGGT[T/G]CACTTTCACGACACTTATGGTCAATCACTTCCAAACATACTNNTATCCCTTC
SFW04577	45.51	TTTGAAAGTCAACTGCACTGTCGGAGTGGTGGTTTCGGCAACACATTGCCCTAGACATTC[A/C]ATTCAAGTCCCCGAATCCGATATNGGATCAAATTTCGGTATGTTGCTNGAAAACATGGA
SFW05990	45.51	AAGCATCNATGAAACGAACATGTAAAGGTTTTGAACTCATAAAATACAAATTGCCAAATC[A/G]CTATTTTAGTTTTTCCCAGACTGTGTCATCTAGGCAGGAGTGTCCATCAATTCTTTTGG
SFW06285	45.51	ATCCTGAATTGAGTAAAATGGCGTCTGATAGCATGAAAAATATGAGACCTGAAGACTTGA[A/G]GCGTGCTGTAGAACAGTTGAAGTCTACTCAGCCAGATGTGTTGGCTGAAGTTGGTGAGA
SFW07542	45.51	GAGGGTTTATTGCCCGAATNTGTCGGTTCGGGTTGATGGAAATGGTGGTAATGGNTCTTT[A/G]GTTGGCGGTTCGAAGGAATGTAGTGTGGGGTTGTGAATTGGTTAAGAAAGATTTTTGAG
SFW01741	45.68	CGACTGCCGTGTACAAGGTGTCATCTGTTGCCGCTCA[A/C]AGTGGCATTCCCATCATTGCCGATGGTGGCATTTCGAATTCCGGACACATNGTCAAAGC
SFW03345	45.68	GTACTTCTACAAGATGATATCGGCGGGGCTGCAAATACTTCATCAAAATCAATGGGTTGA[T/C]GTTCCACCTACTCCTGGCGCGCTTGTGATCAATATTGGTGATTTGTTACA
SFW03552	45.68	GTGGCAATCGACGCCGGAGGAGCAGTTTCGACGGCTNCGGAATCCAGGTGCAGATCATAT[T/C]CGCTTAAAATCGACCGAACTAGCAGACGGGTAATGCCTTTGTCTACCGTTGGATACATG
SFW00549	45.87	TTTGACCGATGTTGANCCACGTATAATTACAACTGGACCACTGGACCCACCGTGAGACTT[A/G]TCCCGGTGTGATCTTTCGTTATCAGCCCTAGATAAGATATTATCGTCTATCCCTTTTTC
SFW01981	45.87	CCGGAACTATTCGTCGTTCCAAGGGTGCCTATGGCATTCATCGGAATCCCGGTTGTGGCA[A/G]AGCATATGGATTTTGGGGCATGACGTTCGTCTGAACCCTAGATCGTCGCATGCCTTCTA
SFW02861	46.22	CAGAAAGTGGTGTGACCGGGAGAGGTGTTGCAAACAGCATATCCATTTAGTTGGATTGTN[A/G]AGTAGTAGTTTATGTGGTAATAAGGTCCTAATCCATGATTTAGTTGTGTTTTATCATTA
SFW06525	46.22	TGGTGAGGGCTACGCANATTCTTGAAATCTCTCAAGTGCACCAACCATAATAAGTATACC[A/G]AGTGGTTCTAGAGATGGTATGCTATAAAAATTCTACTAAATCCATGCTACAGAGGCAGA
SFW01987	46.40	GAAGCCAAAGGTGTGGGTTAAGCCATGGGAGAATTTCAAGGAATTGCAGGAACTCAAACT[T/C]GTTTGATGCTGCCACCTCNCCTTAACTCTATATGGACGGGTGTGCTNTGACAAAACAGC
SFW02478	46.40	CCATTTTAGTCAACAGGGTTCCATGAACCATTTCAACAAC[A/G]GTGGTATGTGGAGCAGAACAGTGGGTATCGCCACCACTCCTACATTAGCAGCCGCTTCT
SFW05176	46.40	AAGATGGTGTGATACCTCTCACCCCAAGTGATGTTTTCATAGATGCTCTAGTATCGCCAT[T/C]GGGTTTCATCCCTATTACCCCAGCGGCCCAGGAAAT
SFW06529	46.40	CACTTTGGTGTTGTAATTGTAGAGCAAAGCAGGAGGGATGATCTGGAGTCTCTTGGTTAT[A/G]TGCTCATGTATTTTGTGAGAGGAAGGGGTTGAAAGCCGGCACAAGAAGTAAAAATACGA

SFW01063	46.48	AGCTAAGTATGTAGGAACAGGNCATGCAGACATGAGCAGATTTGAGTGGGCNGTTAATAT[T/C]CAGAGAGACAGCTATGCGTCATACGTTGGCCATT ATCCCATGCTCGCTTATTTTGCGAT
SFW05198	46.48	ANGTCGCCGCCGCTCGCCACCGTAATTCTCTTTTCAGGGTAAAATGACGACCAGGTTGAG[A/G]AAGAACCGCAAGAAGCGAGGCCACGTGAGCG CNGGACACGGCCGTATCGGAAAACACCG
SFW06860	46.48	TTTCTCGGCATGACTCCGGAGATTCTCCTTAACATCCACCATATCCATAACTTCGCTACC[A/G]ATTTCCTCAAACTTAGTAACGGTACATTCTTGCTCA AAGGTCCTAAGTTTGCCAATATA
SFW07224	46.81	ANTTTCTGCGTCTTGCCCGTTGAATATCTCCTCATGCTCGAAAAATAATAGCCCTAACTC[A/G]TTGAAGAAATGTTTTTCTTCTTTACCTACTTCACTC ACTTTNCAAACTTCNGTTAAACC
SFW00057	47.82	CAANGANTTTTCGGGTGACATTTCTACCCCTCTTTCTCCTCAGTTAAGCATTTTAGACCT[T/C]TCGTTCAACTCGTTTTCCGGAAACATCCCTCGAAC GCTAAACAGCTTGACGCGTTTAAC
SFW06900	48.29	TTCCGGCTTCACATGCCCGAGCAACACCTTCCGTCGGAAATCCGGGTTTTTCGGATCCTT[A/G]ATATTGAACATTATCGATCGGTATTTGAACTTCTG TGCACCGTTGGATCTCCCCCANTT
SFW02249	49.80	TTTAAAAGAATACAACTTCCCAAACCAATCTATTCCATTTGGTCCAAGCTCTTATCTATC[T/C]GCACAAACCAGAGTTATCGAGGAGTACACCATGTC TCAGATAATTTTACAGGCTGTNNT
SFW05859	50.52	GTACAAGTCAAGGATCACTTCGGACTCAAGGCGTGGATTTGCATCTCAGATGAGTTTGAT[A/G]TCTCTGTTAGAAGCAAAGAGATCTTTAAAGCTT TTGGAGGTGAGGTAAAGGAGAAACAT
SFW08376	50.52	TTCTGCANCAAATTCATCAATACTCGTTTTAATTACCGGCTATTGCTATCTTTTTGTTAT[T/C]GGTATTTATGTATCTTAATTTTCTTTTAAACCTTTTTAT TTGATAAATATTGGTTCTTG
SFW02392	50.95	CAATAACATTCCACCGCCTGGATTTATGGCACCACATCCGGCTGCATTTCAGGCTGGAGC[A/G]NNCAAGATGCCNGTTTTTCCAGGCTACGGTTACA TTCCAGTGTGGCAGTATTTACCACC
SFW03464	50.96	TATCCAAAGGATCTGGATGGGTCAACCCAAACCGCAGAAGCTTGACCCAATCATTAAAGT[A/G]TTGGAATAATGTAATTNATGGAGTAGTGGTATTG GAATACTTTAATATAAATTTTAAAG
SFW00232	51.05	CGGTCNNTAAGAAAGCTCGGATCATCCTACTTAGGCGGTGATGACGAATCTGATCTCGAT[A/G]GTAAGNCAAGATGAATTTAAGTTCGGGCTCGAT GACGAGGATGGTATTGTACCAAAAAG
SFW01540	51.07	GGNATCAATNGATGGGTAGTGAACCGGACCATCATCCATCATTTCTGGCAATAGTGGTGC[T/C]GATGGGACNGAGGGTGGTGTNACAATATTAATAG GTGCGGTCGGTACAATTTGAGAATA
SFW03126	51.07	CGATAATCAAGGGTGTTACCTTGGCAAGCACAGGAAGCTTATGCCAACAGCTTTGGAGCG[T/C]ATNATTTGGGGCTTTGGNGACGGGTCACATATA CCCGTTTTTGATACTCCCGTTGGAAA
SFW01884	51.16	TTTCGCGATATCCCTTGTCACGAGATTGCTTGGCCGTATTTACTACCACAAAGATGGACC[T/C]AAGCCTGGAACACTACCGCCTGGGGTGGCTTCAT CGGTTACTGGTGTCGCCCTTGTTGG
SFW07091	51.16	CATTGACATGTTTTGCAGTTGTATGGTAGATGTGGCTAATGAGTTCAATGTTCCAACCTA[T/C]NTGTTCTTCACTTCTAATGCTACTTTTCTTGGGTTT AAATTTTATATCCAGACGCTTAG
SFW07275	51.16	GTTCTATCTCCTTAGCTGTCACAATAATTGTGTCAGCCCTAGGATTAGACACATTCTTCN[T/C]ATAATCCAATTTTATCTCCACCGCCAATCCCAACTC CACCACCATTTCAAACGCATTCA
SFW02829	54.07	TAATACACTAGCCATGAATAAAGGCGGGTCTTCGAATACNAACTTCAGTTACCGAAAAGG[T/G]TTCCCTACTTGGGTTAAGCCGGGTGATCGCAAG CTATTGCAATCTACAAACCCGTCTTC
SFW03316	54.24	ACCAACCCACCGTTTTGGCGACAATGCGATGTGAATTAACAAGCAAAATGTTATTAGAAT[T/C]AAGCTCTACTTTGGAACCTTGTCGGCCTGCCACC ACATATAAACTAGCTATACCTGGAG
SFW04482	54.42	GGTGTTGAGAGGGGATTTAAGAATAAACC[A/G]TACCCGAGGCTTGTGAAGATGGTTCTATCCGCCACACTAACCCAAGATCCGAGCAAGCT
SFW02999	54.45	AACTTTAATCTCCCAATGGATTACTCCAAATCAGTAATCCCCTCACACCTCTTCGGCGAC[A/C]GAGGCTCCAATCTTGTCGTCATTAATCCAGGGTCT GTGAATATACGAATTGGGTTGGCT
SFW06319	56.52	ATTTGGGTAAAACTCAAGCGCCCGTTGGTGTTGATATGAAACCGAAGCCTGATTATTTCT[A/G]AAGGNNGGAAGTTTTGTACTCAGATCCGTTGCCA TTTTTGCTTGATTTTTGTCGAATAT
SFW08932	56.52	AAGATGATGGAAAACCTGGTTAGAATTGCATTGAAATGTGCCGAGGATGATATGCAGGTC[T/C]GACCCACAATGAATCAAGTGGNTAACATGCTTCT ACATCCAGANAAATGGATAGTATAT
SFW03806	57.50	ATCTTCCACCGTTGGTTCAACTGGCCTTCCCAAACCACTATCGATGCCGTCCAACCACCA[A/G]AAACAGAACAACCTCTTCCTACCCAGAGNCTCC ATCATCCCCCATCGGCCGGTTGATGC

SFW06703	57.50	NCTCATATACTTCCACTTTATCTTCTCACCATCTCTCCACCGTCAACCGACTTTCAACTT[T/C]CGGCACCGGCGAACGATGGTACCGGACTCCGAAC TCACCGATCGGCTACAACAAATCCT
SFW03904	64.55	AATGTGGCAGTATTTACCGCAATCTAGTTGTGATACTTCTCATGATCATGAGCTCAGGCC[A/G]CCTGCTGCTTAATAAAAGTGGCTAAGGGACGACA NCCAATGAGAATATTGTTAGCTCAT

[a] taken from Bowers et al. (2012)

Map and sequence-based chromosome walking towards cloning of the male fertility restoration gene *Rf5* linked to R_{11} in sunflower

Guojia Ma[1], Yunming Long[1], Qijian Song[2], Zahirul I. Talukder[1], Md Shamimuzzaman[3], Lili Qi[3]*

Supplementary Table S3a Sequences of SNPs selected from the HA-R9 whole-genome resequence for *Rf5* in the present study

*the sequence with lowercase letters are repetitive in the reference genome

SNP	Target sequence
C13_175223520	*atgacctaattttatgttgtctaataattaaaacatgacctaatttaaataaccaaatgttattgaacaatataaaaaagcccttacatttgatccaattaaccttaataatgggttaggctttgaaggcccattaaacccttataacaaatacgaccccaaattttaatcactcatttaccgctccatctccgttcttc[g/a]aagggcgacaacgaaatgacagacaaatcttcagatcatattgaaacacgggtcgtttcttaggaaaccatgcataacatattttaatagaaagtataaataggtgtatagatattctattttttttagttttttgtttgattaatattaaaaactctgtttgaggtatctaaaagtctgtttgactaaagtcaaccac
C13_175225873	atcggggcatggcactaagcgaaacagattgtccagaagtttccataacaattatcattactatttaatttaaaataacatgtcccataccatgcctcaaatagtaaacaaattattacagacaaaaaatctaggcaattaattctgttccgacaactcagatttaaatatagcaattgtttatctgcttctagagaccc[c/a]tgattcagtttgtacagacaactatttgttggactctagagccttattctagcctcgctttcctaacagataagcccccctaaacacctgtcacatacgttaaaataaagacaatacacatagtgtaaaggcgagtatacaagtttgataatggcatatggatttcgaaatagtttacgcataaccaacacgtacacagag
C13_175227801	cacattagggtgatactttgtcgtttgtcgttaaatcgataaatgagttgaagtatagcactttgtcgttcattgtgagaatttgatctcgtgagttatcgtaactactgtattgatcactaaccctgttttgtgtgcattattgtgaaattaggttaacagggctaaactcttattctatcagtactaaatctgcaatg[t/c]gagtcattctcttttattaactgttttacaatactccaaatca_ttttcagaattataattacagtgattaagttgatgtaatcaccaaattacagccagtatgtggggtattgtgcacattactacagtttaaatcatttttggtgggcgaacctaatttaaatgatttacgtcattcattggggcagccaatggtgat
C13_175227854	tatagcactttgtcgttcattgtgagaatttgatctcgtgagttatcgtaactactgtattgatcactaaccctgttttgtgtgcattattgtgaaattaggttaacagggctaaactcttattctatcagtactaaatctgcaatgtgagtcattctcttttattaactgttttacaatactccaaatcattttcaga[a/g]ttataattacagtgattaagttgatgtaatcaccaaattacagccagtatgtggggtattgtgcacattactacagtttaaatcatttttggtgggcgaacctaatttaaatgatttacgtcattcattggggcagccaatggtgatatgaccactgtcacagatccggtcgagtgacaaatactgtgggtagttggttg
C13_175228962	ggcacaagttccatttctattggttttgaaggcattgagtgtaaaagagttgaaagaaagttggaaaaaccatctttcaatccctttcaccgtgaatatgtttagatctgtgaagaatatttgtttgtttatgtggaaatcggttagatccaagttattcttggtggattgaggccaaaacatgaagttcttcaagaaca[a/c]atgattacgtcatcctagaataccttgaatcttgatgatttcacggttaaaagttaagatttaaaagatagaaaggtgtaggagtgcgtgtagatcaagaaagtacaagatttaggacgagatcttaccggagtcgagagaaatctgagaaaaggtgaggagcacgagctggtcggacgtcacttgccaaaagtcgtaag
C13_175229095	tggaaatcggttagatccaagttattcttggtggattgaggccaaaacatgaagttcttcaagaacaaatgattacgtcatcctagaataccttgaatcttgatgatttcacggttaaaagttaagatttaaaagatagaaaggtgtaggagtgcgtgtagatcaagaaagtacaagatttaggacgagatcttaccgga[g/a]tcgagagaaatctgagaaaaggtgaggagcacgagctggtcggacgtcacttgccaaaagtcgtaagttggagagtgacaagaggtatttataggatgctaaaagaggaaatgggtggccgatcggccaggcagctcgatcggacagcctgtctgatcggacggcagtccgatcggccggctggtcgatcggctggtagc
C13_175230552	tgcgtcgtctatcggcgtaacttttctgtctacttcgagctttcaacaagttgtctcggatttgtaggactttgtcagtcgtttcttgcagtagctcaggaccagttaattgcgagtgaccaatctcgtgccatacaataggcgatcgacatcttcttccgtataaagcctcgaatggtgtcatttggatgctggtatga[c/t]aactgttgttgtacgagaattcgactaacggcaggtatttatcccaattaccaccgaagtctatgacacacgagcggagcatgtcttcaagagtacgaatcgttctttcagtctgtccgtcggtttgggggtggaatgcggtacttaagttaagcgtcgtaccgagagccgcttgaaacgtttcccacaatcgcgaggta
C13_175230845	gtacgaatcgttctttcagtctgtccgtcggtttgggggtggaatgcggtacttaagttaagcgtcgtaccgagagccgcttgaaacgtttcccacaatcgcgaggtaaaccaagcatcacggtcagagatgatgtcacgaggtgtaccatgattacagatgatctcgtcggtgtagattcgggctaatcgttctacctt[g/t]tagtcttcccgtatcggcaaaaagtgagcagatttggtcaatcgatcaacaacaacccaaatgctgtcgtgacctgatgacgtgtgcggaagctttgttatgaagtccatagctatactctcccacttccatatcgggatcggcggttgttcgagtaagccagaggatctttgatgttcagctttgactcttgcacaagt
C13_175231256	ccgacgtagagagcgatatccctttcaagatcctgatacattttgtcggcaccgggatgaatagaatatcgggatttgtgggcctcgtccatcaaaatctttcgaaaatcggtccgcttagggatccaaattcggtctagaaaatagaagatcccattcgatttacttacaagctgagctccatcgtgatagattcttt[t/c]cttcttcaaagtgtgttcgttgaaacaagcgtgctgagcttcgcggatgagggtttcgagatcgtgttgggattgggtattacgaatgctgagcaaataactccgcctgctgagcgcgtcggcaacaatatttgctttgcctgggtgataacgaatctcacattcgtaatcgttgagaagttctacccatcggcgttgac
C13_175231557	ctccgcctgctgagcgcgtcggcaacaatatttgctttgcctgggtgataacgaatctcacattcgtaatcgttgagaagttctacccatcggcgttgacgcatattaagttctttctaatcaaagatgtgttgtaaactcctgtgattggtgaagatcgtacacttggtaccatacaggtagtgtcgccaaatctttaa[c/t]gcaaaaacaaccgcgcctagctcgagatcaagggttgtatagttcttttcgtggattttgagctgacgagatgcgtaagctataaccttgtcccgctgcataagaacacaaccaagaccaagattggaagcatcacaatagataatgaagtcgttgtttccgtcgggcaatgtgagaacaggagcattgcaaagcatgtg
C13_175232324	gacatctcgatgaatacgatgacgaaacggtctagaaatggcttacacacacgactcatcagatccatgaaaaccgcaggtgcgtttgttaaaccaaaatgcatgactatgaactcataatggccgtagcgagtgcgaaaagcggttttgggtatatcttcttcctgaatgcgtagttgatgatagcctgagcgtaaatc[g/a]atcttcgagaaacacgtagcaccttgcagttaatcaaacaaatcatcgatatgaggcagaggatagcggttcttgatggttaacttattcaactcccgatagtcgatgcacatcctgaacgacccatccttctttttaacgaagaggactagcgcgccccccaaggagaggtgctcgggcaaatgaaacctttctcaagta
C13_175232776	aaggagctttggctacagggttggctccagggatgaggtcgatacgaaagtcgatatcacgacttggtgatagaccgggaaggtcgccagggaacacatcaggaaattcatgaaccacaggaacgtctttcacttttgtctttcctttcgtttccttctccgctactacaatgttagccaagaaagctctgtattccttg[t/c]ggagatacttgctagcctggacacatgacatgagcttgagtcctttcaaagcagtttcatcatacacacataaagaatcaccattcgcgagcgagaatcaaatcatcttatcgaagcacacaacttcagcatggttttcgcgaaggaagtccatgcctactatgatgtcaaaacttctgagttgcatcggaatcaggtcg
C13_175233623	atgagcagtggcttgaactggggcttggcgaggaccagtacggcaattagcagtgaaatggccgtacatgttgctgtgagcgcaaaaccgataggcaagacccaccagatgatgatatgggcatgctgggcaaaccgggtgggggcctgtgtatgcacgctttgctggcggtgcatatgtaactggtgctggtcggtgat[g/a]agattgctgctgagccggtacggcttggagaggagcaacagtggtagtgacagcacagttcttgttgctggagctgttgttgttcttcttcttcttgcgacgtgatgactttgacggttgagcggtggcggtttcagcggttggtggagtggtagcttgatgcagagacttgggggggcttatcccaaaaaccagtttta

C13_175233818	gtgatgagattgctgctgagccggtacggcttggagaggagcaacagtggtagtgacagcacagttcttgttgctggagctgttgttgttcttcttcttcttgcgacgtgatgactttgacggttgagcggtggcggtttcagcggttggtggagtggtagcttgatgcagagacttgggggg<br>gcttatcccaaaaacca[g/gc]ttttactcgcttgtcgttgatttcagcggcaagcaagtaagtctcttcaatcgatgatgtcttcgcggcgtgaacaaaatctgcaacacagtcgggtaaagctcggatgtacttcttgattgccatatcagacgtcttgacgtgatcagggcagataatgctg<br>agctgcttaaagcgagcagtcagggcagcgttgtctc
C13_175234341	ccgcgtttgtttctctcggtcgtccaccagtctaaagtgcgggactggaagactccggtagcattgagggtacggagattatcaggacatccgctttggcgcagagtgacttcgacggaattgaaccattggaacatcgctgttgggccatcttccccagtgaattcctttggaccgcatgcc<br>ttgaactgtttaaaact[a/g]aaagcagtcttgggagcaactttaggagcatctgttctagactcctcagacgacttgctgcgttctcatagacatcactcacagcttttgctacttgcttggcgatgatagcagtaagacgcctgtctctcttttcttaacgagtaagtgggtgacgggatcctg<br>atgatggtccagttgacattgtctgcaatagacat
C13_175234439	gcgcagagtgacttcgacggaattgaaccattggaacatcgctgttgggccatcttccccagtgaattcctttggaccgcatgccttgaactgtttaaaactaaaagcagtcttgggagcaactttaggagcatctgttctagactcctcagacgacttgctgcgttctcatagacatcactcac<br>agcttttgctacttg[c/t]ttggcgatgatagcagtaagacgcctgtctctcttttcttaacgagtaagtgggtgacgggatcctgatgatggtccagttgacattgtctgcaatagacattgtcgtaggtctcagacacgtcataaccgaatctcacctcaaatgtctaacacttactaaagctca<br>ggaacacagtcgcataattacttaaacacata
C13_175235054	gtcgtccatattgtagcgacttgttcgcgtgttgagatagaagtgcagtgtgagtcgtgcgtgtcgatcgagcaattctccaaaagtttaaacacataaaaaggtaaattcacgtataaacacataaaacgacgaatcatcagagtcagcagttgcgggatcagaatcaaaacacacaaaat<br>cgggaagtggattgtctg[c/t]gactattagacattgactacccaaagtgattcgactattctcaaggacttggtgtgcacattttgactttcaggattgggcctctgcctcgaatcttgggcatccggcgcatatccctcttgtcatatcgtgcgttcaggtcgttggagtccgtcgtgactttgg<br>tgagagttttgtaaaaaccatggataagccgtcaa
C13_175235291	attctcaaggacttggtgtgcacattttgactttcaggattgggcctctgcctcgaatcttgggcatccggcgcatatccctcttgtcatatcgtgcgttcaggtcgttggagtccgtcgtgactttggtgagagttttgtaaaaaccatggataagccgtcaaggttagggtttcacccctggctt<br>gacaacttccctt[t/C]gattttaataaatagaggagattattcatcaaaatatggatttcactcctgatttggtatacttctccccgttggtaagtaaaagcgcgggtcgatttagcagattatggtcgagagtttgcggttttcacacctattctcgacttaaccacttgtctttatatgaaaattcg<br>agtgcagtaacagtgtagtgtagatga
C13_175235714	gctcatacataatccctaatggacatgcacgagtcggtctattgggtcctaactatagactaggtctctaagacatcgacccggactaggtcgagtcttgcctaattccctatagttatggctctgataccaatctgtcacaccccaaccgatggcggaatcatcggggcatggcactgagcg<br>aaacagattgtccagaa[c/g]tttccataacaattatcattactatttaatttaaaataacatgtcccataccatgcctcaaacagtcaacaaattattacagacaaaaaatataggcaattaattttgttccgacaactcagatttaaatatagcaattgtttatctgcttctagagacccttcattca<br>ctttgtacagacaactatttgttggactcta
C13_175237524	cagctcgtgctcctcaccttttctcagatttctctcgactccggtaagatctcgtcctaaatcttgtactttcttgatctacacgcactcctacacctttctatcttttaaatcttaacttttaaccgtgaaatcatgaagattcaaggtattctaggatgacgtcatcatgtgttcttgaagaacttcatgtt<br>ttggcc[t/c]caatccaccaagaataacttggatctaaccgatttccacataaacaaacaaagatccttcacagatctaaacatattcacggtgaaagggattgaaagatggtttttccagctttctttcaactcttttacactcaatgctttcaaaaccgatagaaacggaacttgtgccgactta<br>ctaatcattccggtggtcgagtg
C13_175237731	aacatgaagttcttcaagaacacatgatgacgtcatcctagaataccttgaatcttcatgatttcacggttaaaagttaagatttaaaagatagaaaggtgtaggagtgcgtgtagatcaagaaagtacaagatttaggacgagatcttaccggagtcgagagaaatctgagaaaaggtgag<br>gagcacgagctggtcgga[t/c]gtcacttgccaaaagtagtaagttggagagtgacaaggggtatttataggatgccaaagaaggaaatggctggccgatcggccaggcagctcgatcggacagcctgtcctgtgacaactcgtatttcgaacctatctttgtgtaacgttatatgattac<br>gtgaactttattaattgaatgaatgtatgttatgttatatg
C13_175238533	tgttttgaattatgtagaatgataattatgtgctaatgatctagaaccgattgaatgataatcggctgtgatatgttcctgttcgaatgatttgtggagggggattgttatgttagtatgcatgctagtgatcaagaatatgaatcatgttaatatgtttaagtgttatgattcttgttcacgctttgattatg<br>attagg[g/ga]ttcatatgaattttgtgataaaaacccttgtttgatcaaatattgattaagtttgaaactgttgattgtgattgattgatcgaataaatggaaactttgtaattgatttgcaagataaatgttatgtaactgattgccataatattcggaatttaaaaccaatcgcacaagggctctgatc<br>gcacaagagcccaatcaca
C13_175238718	ttgattatgattagggttcatatgaattttgtgataaaaacccttgtttgatcaaatattgattaagtttgaaactgttgattgtgattgattgatcgaataaatggaaactttgtaattgatttgcaagataaatgttatgtaactgattgccataatattcggaatttaaaaccaatcgcacaagggctct<br>gatcgca[c/t]aagagcccaatcacacaagtgaatcgcacaagttagaatatgctaaggtacaactgatgatacactaggtactgtttaatgatgcatgatcgcacaagaccttgtgggtcgcacaagacagtgccgatctcacaaggcttgggccacacacacatacagttggaccttat<br>tatgttgattgggcctcaaagcccaatccc
C13_175241961	gctccagtattatttgttaaaaagaaggacggtaccttccgcatgtgcatagattaccgcgaactgaacaaggtgaccgtgaagaaccgctatcctcttccacgtattgacgacttattcgaccaactgcaagggtcgagctactactcaaagatagatctgaggtcagggtatcatcaattg<br>agagtccgagatgagga[c/t]gtctccaaaacagcattcagaactcgctacggccactacgagtttctagtcatgccattcgggttaacaaacgcacctgcagtctttatggatcttatgaacagagtgtgcaagccttatctagacaagttcgtcatcgttttcatcgacgacattctgatcta<br>ctccaagagtcaggaggaacacgaacaacatttacg
C13_175243512	gaaatttccatggattttgttactggcctacctagatcccagcgtgggaacgatactatttgggtgatcatggattgactcacaaagtctgctcatttcttggctataaaagaaacggataagctctctaccttagcagacgtctacttgaaagaagtggtttcgaggcacggagtgccaacctct<br>attatttcggatcg[g/T]gatgcacgattcacttcagaactgtggcaagcaatgcacaaatcttttggctcacgattagacatgagcacagcttatcaccctcagacggatgggcagtctgagcgcactatccagactctagaagacatgcttcgggcatgtgttatcgatttcagcaacag<br>ctgggaaaaacatctccctttggtggagttctcgta
C13_175243669	tggtttcgaggcacggagtgccaacctctattatttcggatcgggatgcacgattcacttcagaactgtggcaagcaatgcacaaatcttttggctcacgattagacatgagcacagcttatcaccctcagacggatgggcagtctgagcgcactatccagactctagaagacatgcttcgg<br>gcatgtgttatcgatttc[a/g]gcaacagctgggaaaaacatctccctttggtggagttctcgtacaataacagttaccacaccagcattcaagccgctccattcgaggcattgtacggacgtaaatgccggtcacctctctgttgggcagaggtgggggatagtcaaatcacaggtccag<br>aaatggtagttgatgctactgaacggattgcacagatacgg
C13_175244842	ttatcattctacataattcaaaacatgttacacatatgaaccgatttcatgaacatcatacttcatcagttttcattacaatcaaacgcataccatctctaaatcatatgtaaaacatataagtcaacacatcgtaaatcatgaaatcaaaagtattacactaaccggttaaagaatggaacgaggatt<br>gataaagcttc[A/g]agagagatgagatgtggctgccgtcaagtttgagaggagagaaagagagtagggtttgtaaacttgtgtaattaggtaaagtgtgggatttataccaacaccctaagtgttgtgtgagttgatgaagtgggccgaaccctcctaatgggctgccctttatgggtttc<br>gagtacaacaaggtggagcccaaatggacttgt
C13_175245069	ctttaaccggttagtgtaatacttttgatttcatgatttacgatgtgttgacttatatgttttacatatgatttagagatggtatgcgtttgattgtaatgaaaactgatgaagtatgatgttcatgaaatcggttcatatgtgtaacatgttttgaattatgtagaatgataattatgtgctaatgatctagaac<br>cgat[t/c]gaatgataatcggctgtgatatgttcctgttcgaatgatttgtggagggggattgttatgttagtatgcatgctagtgatcaagaatatgaatcatgttaatatgtttaagtgttatgattcttgttcatgctttgattatgattagggttcatatgaattttgtgataaaaacccttgtttaatc<br>gaatattgattaag

C13_175245777	atacacagaaaggttacttgagctaaggggtcaaccacgtcctaacatggttaccatacaagtcagcttttggtttcaagtaatgcacgtatgtttacgcataactaacaggtttcgagcacttatggatcatggcaaacacataacagtcacgagcaatacacattcatacttgtgcgattcaa<br>aagaatgggctcgca[t/c]aagcctagcagccttgtgcgattcacttgttgtagcccaaaaatacccggcccaaataatatattagtccaaacatatacacggcctaacagttaatcgaaaagtaaattgtgccatccaattagtcttgtatgatccggacgacttgtgcgatccggataactt<br>gtgcgattgggattgggctttgaggcccaatcaac
C13_175246065	acctgttagttatgcgtaaacatacgtgcattacttgaaaccaaaagctgacttgtatggtaaccatgttaggacgtggttgacccccttagctcaagtaacctttctgtgtatctgccgagcaaaccaaggtgagttcacacccttaccaaggcatgggattcccggtgggtagggaatgggat<br>tgaaggaatgggattg[t/a]acaactacttggacttacactgttactagactatctaccatcgtcctcggttgggaaggacccttacgtaaaacctacgtaaacatgcgcttactactgtcctcagggttcgagggacacttacgtaaaacctacgtaaccttatgcttactactgcctcgggtc<br>atgaagggcacttacgtaaaacctacgtaaacccc
C13_175252320	ACGTTTATGACCCTTAAAGATCCTACAAAGCAAATAACTGTCGAAAAGTAGTTAGATGTGGAAAACGAACAAGAGGGTGTTTGCGTTGGAAAGCCTGCGCTACATGTGGAGTTA<br>AAGATCAGAACCGAGGATTCAACTCATGTTGGACATATATTAACGAGGGGGGCCACCTAATGATTGGATATTGGGACCAAATTCAA[A/C]AGAAAGAAAATCATTGCAATGAAG<br>GCTGGCATGAAACTGGTGATATTGGGCGGATAAACGATAATGGTAATCTATGGCTCATTGGGCGCATGAAAGGGCGTATCAAAAGTGGAGGCGAAAACATTTATCCTGAAGAG<br>GTAAGCATCATCAAATTACAAAAGCTGATTTAACTGGTATAAGTTAGGTTTGAAATTTAACGG
C13_175253519	CAGCTCTAAATACAGCAAAGTATTCCACACTTACAAAAAGGGATGATTTATGTTTTTAGTCAGCTCAAGTTTCACCGGATGGAACGTTATGACAAGCTTCTAGTAGGATCTGTAC<br>TCTGTTAATTTGGAATCTTGACACTTATTTTCCATGATCATGAAGGACATACCCATACAGGTTCAAGTGAAGAACATGTGCACCG[T/G]AAGCTTTTTATGCTATTATGAGAATCT<br>TAACAGGTCCTGTAACCCTTGGCCCTTTAAAAAAAACTTTATTTTCGATTACTTTTTTTATGTTGTCACACAGGTGGTGGTTGTCTATTGATGACAATGGAAATCCTAATTATATG<br>AAAATCTGATGTATCTTGTAAAGTATGGCGATTCTGGGGCACATCCACTGCAGAATT
C13_175253964	GTGATCAACGCTGTGATATCAACTTTGTAGATATCAAACCGGAATTCATATATAATGTCGATGAAATGCGACTGCTAGTATTAAAAATACAAATATGTTGAACAATAAACTCATT<br>CGATAGAATATACCACAAGTAAAGACCAAAACTAATTCGATACAAATTTCCAATTTTAATTTCTTATCCTACAAAAGTACAAATA[C/G]CTCAAACCAACAATAACTAGTTCAGT<br>CTCTCTCCCAGATTGTTATCTAGGCACTGCAGTAGTATCCATCATTTCTTTTGGGACAAGTTTACCTATCAATTTAAGCATACTTCTATCTACTGAACCAGCTGCGATTTTCTCAA<br>TCAACATCGATAAAGTTGAAGCATCAACTGAGTACCTTCTTCCATCCATTTCCTCTAA
C13_175254345	CTTCCATCCATTTCCTCTAAGAGCATCTCAACATCATCGGAATTCTGGTTCTTCAGATATCCTTGGAGAAGAACATTGTAGGTAACACTATTTGGTGGGCAGCCACTCTCATCCA<br>TTCTATGAAACAAGTGCTTTGCTTCCTCCAGCATACCTTCTCTACATAGGCCACTAACCATCACGGTATATGTCCGAACATTAGG[T/C]TTCAAACCTTTTAGAGTTAGGTCTTGG<br>AAAAGATCCCTTGCAATATCAAGCTTCCCACATTTGCTCATACCATCAATAAGAATATTGTACACTTGAATGTCAGTGTTTAGCTTGCTATCACCCACCAAATGAAACAAATAG<br>AGCACATCTTCAACTTGATGGTTGTTGCAAAGACCGGCTAATATTATTCTATAGGTGCA
C13_175254871	TTCTTGTTATCTCATGAAACAAATGCATGGCCTCTTCTATTTTCAAACTCTTGCAATACCCGTTCAGTAAACTGCTATAAGTTACAACATTAGGGACGAGACCTTTAAAAGTAAG<br>TGAATCGAAAAGCGTCCTCGCTTTGATCATTTCACCTCGTAGGCAGTAACCGTCTATAAGTGAACTGTATGTCACTATGTCAGGA[G/A]CCTCACCTCTCTCAACCATGATGTCT<br>ATAACAGCCTCCGCTTCATTCACCTTACCATCCTTGCAAAATGCATCAACTAACATGTTAAAGGTTTGCAAATCAGGAGAGATCCTCACATCCTCCATTTCTTTCAGCAGCTTAT<br>AGACCTCACCCCAACGACCTAAGTTACAAAGGCCATGAATCAAAGAGGTGTATGTGATG
C13_175255359	ATGATGGTGCTATATACAACAACATTAGGCTCACAGCCTCTTTCATCCATGAGCCTAAGCAAACCAATGGCAATATCATTATTGCCAAACTTGCAAAGCCCTTTGATCATAGTG<br>GAATACATAACTACATCAGGTTCACATAGTTTCTGTTTGATGAGCTTCTTGAAGAGCCTCTCAGCCTCAATAATCCTATCTTCTAG[C/G]ACCAGTCCATCTAAGAGTGTACTAAA<br>TACGAACACATTTGGTACAACAGCACGCCTGAAGCAAGAAGCTAGGAGTGCAAAACCGTCATTGGTGCGAGACATCTGACAACAACACTTGATTGCAATACTGATAGAGTACT<br>CGTTAACAGGCACTCCAATGAGACACATTTGTTTGAAAAGATGGAGAGAAGAAGAAAAATG
C13_175255719	CCCTTCATCGATTCTGCTTCTCTTCTCTGTTGGATCATGATGACTCGTCGTGCTTTCATCGAGCTCAAAGGTATTTTCATCACCAATTCTTTAACAACAACCCCTCTTTCATCTCCC<br>CAATTAGGTTTACAATCCACTCTTTTAGCTTCTCTTCTTCACTCCACTTCCTTTTCAAATAACGGGGATCGTCTTCTTCACTC[C/T]ACTTCCTTTTCAAATATTGGGGATCGTCCC<br>CTGTCTAGGTATCAAAAAGTTACCAGCTTGGATCATGCCTTTAACCTGTTCGATGAAATGATTCAAAGACGACCACTGCCATCTGTCGTTAAGTTTAATCAGTTGTTAAATTCTG<br>TTGCCAAAATGAAACATTTTTCTTCTTCTCTCCATCTTTTCAAACAAATGTGTCT
C13_175256815	ATTTCTTCAACGATTGACGAAATTTGAATGCTAGAAACACTCATCAGCGATTGATCGAACGAATCGAGTGATTCGCTTCAAAATCACGGGAAAAATGAGATATTGTATGAAATT<br>AAACTGGGTTTTCTTTCAAAAAAAGTTGAAGAACATATTGATTGGGTGTTTGAATCATTGACTGGTGATAAAAATCGTACTATAAT[G/A]TAGTGATTATTGAGATAGAAAGTGA<br>AGAAAGTTGAATATAGTGGGTTTTGAAATTACAGGGGAGAAGATTGGATAAATTGACTGAAATACCCTTCTCCTTCATTTTTAAATTTTGACAAATATTATAATTGTGCTTTCTAT<br>TCTTACTACTCAAACTAAACTTTCTATTTGACTCCCTCCTTATGAAACAATTCAAATAA

C13_175258189	TTCGGATCGAACCACAACCCATTTAGCATCCCTAATGAAATATATGAGTAACTTAAATAAAAATAACTAAAAACAAAAAAGGTTTATAAAAATCTATTTTTATGGAAGTTTAAA<br>AAATATGGGTTATTGGATTTTAATAATCCAAACTTTGACCATTTGGCCCGCAATACTCTCAACTTAAAAAATTCCCCCAGCCAGTC[T/C]CAACTTTCAAGTTATTTTTCTCCACC<br>AATCCTTTACTAACTGGGTTATAAGTATAACCCAGTTAGTGTTTTGTGTACGTGGCAGCTTATGTGGCACACAACCTGGTTAAATGATGACATGGAAGCTTACGTGGCACCATCA<br>CCACCTTCATCCCCACCACCCACAACCACCGTTTGCCACCACCATCACTACCACACACC
C13_175259348	TAATGTGATGATTAGTGGCCTATGTAGAGAAGGTATGCCAGAGGAAGCAAAGCACTTGTTTCGTAAAATGGGTGGGAGTGGCTGTCCACCAGATGGTGTTACTTACAATGTTCT<br>TCTCCAGGGATATCTTAAGAAGCAGAATTACGATGATGTTGAGATGCTTTTACAGGAAATGGATGGAAGAAGGTACTCACTTGATG[T/C]TTCAACTTTATCGATGTTGATTGAT<br>GAAATCGCAGCTGGTTCAGTAGATAGAAGTATGCTTAAATTGATATCTACAAAGTGTTGATATCTATATAAATGAATTCTGCAGTGGATGTGCCCCAGAATCGCCATACTTGTTC<br>AGTTGTTGCACATTCTTCCTGCCCAACGGTTGTGGGTAGTCACATTAATTTCTATTTCTA
C13_175260181	ATTGATAGTCTTTGCAATGACAAAATGATAGAGGCTGCTTTCAAGCTTTTCAATGAAATGGTATTTGCCAAAGGCATTCAACCAGATGTCATCACATACACCTCTCTGATTTATG<br>GCCTTTGTAACTTGTGTCGTTGGGACGAGGTCTCTAAGCTGCTAAAAGAAATGGAGGATCTAAGGATCTCTCCTAGTTTGCGAAC[A/C]TTTAGCATATTAGTTGATGCATTTTGC<br>AAGGAAGGTAGGGTGGATGAAGCGGAGGCTGTTATAGACATCATGGATGAGAGAGGTGAGGCTCCTGACATAGTGACATACAGTTCACTTATAGACAGTTACTGCCTACGAGG<br>TGAAATGATCAAAGCAAGGATGCTTTTTGATTCACTTACTTCTAAAGGTCTCATCCCTGA
C13_175261496	TCTTTTTTAAATAAAACTCTGAAACACAAAATATATTTATTTTCTTTTTATTTCTTTTCCTTTCCTTTCATTAGTAAATGAAACTTGAGAACATAACATGTTTTATTTTCCCTTTTTT<br>TATTTCCTTAAATAAAACTCTAGAACACAAAAATATTTTTACTTTCTTTCTATTAGCGAAGTCTGGAACAATATAACATCAT[T/C]TATATTATGATTTTTAGAGTAAAATGCTTGG<br>ACGCTCCATGTGGTTTGGTAAAATAATGCATATAGTCCCCAACTTTTGGAAATTACATGTATGCTTTTTGTGGTTTGCTCGTTTGTTACTAGGATAGTCTCTGGAGTGAATGGACA<br>TTAGTTTTCTCAGTCTAGTAGATATGAAATGACAAACTTACCCTTAATGATAA
C13_175263312	AAAGTCAAAACCACACATAGAGGTCAAAAACTGTTAGAAAATTACCGCTGCATAAACTGCAAAATAATTAGAGACAGAAAGAAATAGTTAACGAGTGAACCGGGCTTGTGTTT<br>GACACAATTCCCTTAAACAGATTCGTCGTCTGTTCCCAGGGTACGCCGGTTCGAAGCAGCACCGCGCACTGCCGGACTTGAGCACTT[T/G]CCTGGAAAGTAAACCGGAATGAT<br>GTTGTAGAGACCGAGAGAGAAGAGAGGAGAAGTTGTTTTTGTGTGTCTCAACTGATCAGCTTTGCCTTTGATTTATAGTGTCAGAACTATAACCGAAATCAGCAAATTAAAGAC<br>CAAATAATGGTCATTAGAATATCAGTTACGAAACTGATAATCATTTTGAAAAATGAAATAATG
C13_175264594	GAATCTGAGTTCATCGCTTTAGATAAAGCAGGTGAAGAGGCAGAATGGCTACGTCAATTCGTTGAGGATATACCAAGATGGCCTAAGCCGGCAACGGCCATTTGTATACATTGT<br>GATAGCCAATCGGCACTTGGCAGAGCTCGTAGCACAATGTATAATGGTAGGAACAGACATATCAGACGTAGACATAATACGATACG[G/A]CAACTAATCTCTACAGGAATTATC<br>ACTGTTGACTACGTGAGGTCAAAGGATAATATTGCGGATCCGCTGACAAAAGGCCTAAGTAGAGAGTTAGTACAGAAGTCGTCCGTGGGAATGGGACTGAAGCCCTTGAAAAA<br>TGAAGTTCATATGATGGAAACCTAACTCAATAGACTGGAGATCCCAAGAATTGAGTTCAATAG
C13_175266436	CATATTCCAGGATTCTTAGAATCATGCACAAGAAAACGATAAGGACCATAAGGACGGGTGTATCCTATAAATACACAATCCACCGTTTTGGGTCCTATTAGTAGGCGCTTAGGT<br>GGAGTGACCACCACCTTAGCTAAACACCCCCACACTTTCAAGGATTTATATGGTGGTTTCTTTCCCGTCCATAATTCGTAAGGCGT[T/A]ACATCCTTTTTCTTATTGGGTATCAT<br>GTTCAACACATAATTTGCCGATAAGATTGCTTCCCCCCACATGTTTTGGTTTACACCAGAACTTATCATCATGGCATTCATCATTTCTTTCAAAGTACGATTTTTCCGTTCCGCTA<br>TGCCATTTGATTGAGGGGAGTAAGGAGCCGTGAGTTCGTGGATGATTCCACTCTTTGC
C13_175268380	CACAGGAAATCCGAATGATTCCAAGCATGCATCGCGCTCATAGTTTGAGCATCTATCTCCCCTTCCTCAACATGGGGTTTCGGTTCAGTCAAGAACCTTGCCAGATTCAGCGTG<br>GTCAGATAAAAGAACATCTTTTGCTGCCACTGTTTGAAATTCACACCACTGAATTTCTCTGGTTTCTCAGCATGCGACACTGCATT[A/C]GGCAGTGGTGTCACTAGACCAGTTGT<br>TACAGCAACAACGGAAGTCATGACAGCAGTCACGACTGTAGAAACAGCAGTGGTCACTGGGGTTTCCATTCTGAAAAGTAAACAGGAACAATCAGTAAATAAAATCTGTTTAA<br>GTTTGTTAGAAAATTACCGCTGCATAAACTGCAAAATAATTAGAGACAGAAAGAAATAGTT
C13_175269779	ACCGTAAGAGTCTTCACAGTTCTAACTGTAGCCGTCATTTCTCGCCTCTATTTAGTAACAATCATAACTAAATGTTCTTTGTATGAACATACAGCGTAAGATTGTGATTCATCATT<br>CATTACACTAGATATCAAAATGGTTATTTGAAGCACAAGTTCGACTAAGAAAAGTCAAATGTGTGAAATTGTTAGACAACTATG[G/C]CTTTGGAACGTTTTTAGATGTGTATTA<br>TATTATTTCGAAACAAAAAGTGGATTAATAAAAGGTAAGTATTTGACGAGTCAATGAAAGAATAAAGTATGATTTTATATAAAAACCCATTAAAGACAACATACACAACTCGGG<br>GGTGTTTGGCTAAGCTTTTCAACACCTCCTTATAACTTATTGACTTTTACAAAAAGCTA

C13_175273046	AAAATCGGAACAACATCAGTAAGCAAACAGACCCCAAACAGGTTTAATAATCCAAGGAATGTTAGTGATTCCTTGATAAGCCTGTGCTGCAGAAGGTTGAACTTTCTGCACATC<br>TTTCATGTAATACTCTCTACCCACCCTACCAAGAGCTCCACCAAACCCTTGATTTATTCCATAAACCACCACAACCCCACTAACAA[A/G]ACTCCAATGTGTTTTTCTTGATAAC<br>ATACCACAAAAGCCCCTCTTTTTGGGCCTCCATTTTCATTCTTCAACAACCTCTTTATTGCAGGTTTCATGGTCTTTTCCTTCTTCCACCATCAATATCAACTTTTTTTCACAAGAA<br>AAGTGTTTTTTTTCTCAAATGGGTATTTCAAGATTTATTCTTTTGAGACTAAAGGGTAT
C13_175275104	CTCCCCTAACACCCTAAATTGATGGCGGCACTCCCCTCTTAGGTGAATTGGTTTTTTTTTTTTTTTTTTTTTTTTTTTTTTTTTTTTAAAAAGCATTGATTGGTCACTCCCTCTCTCTC<br>TCCTCCCTCTCTCTTCGGTGAGCCGCCACCGGTTCTCTCTCCTCTCTCTACTCACCGCATGGATGGCGGTGTTCTTGCCGA[G/C]CGGGAAAAGGGGTCACCGCATGGGGTTGCC<br>GAAGGCACCCCGTGCACCCTTATTAAATTTTAAACCGATCCCTAATCCATTTCTTAAATTCCCAACAAGAGCCCTCTTTGCCACTGACTGACCGCTTCATGATCGTTGATTTCTT<br>GTACCTGCCAATAAGAATCATTCTTTGCTTTTTATGCCCTCTCGGCAACCTAGTT
C13_175276537	GTGGCACCATCACCACCTTCATCACCACCACCCACAACCACCGTCTGCCACCACCATCACCACCACACACCGCGGCAACTTCTTCAGGCGACCTGCAACTTGTTCCGGCGACA<br>CTCTTTACCTATGAAACCGCCAAACGAGTATCTTGTAACAGGTGGTTGACTGTTGATTACAATGGAAATCCTAGTTATATGAAAATC[T/C]GATGTATCTTGTAAAGTTCTTCAAA<br>ATCTAATTCTAGTTATATCAAAATCCTTGTTATACTGATTGCATCTTTGATGAAAAGGGAAACTGGTTTTGGTTCTGTTTGAAGTAAGTGTTGGAATACTTGATCAAGCAAACTTG<br>AATAACACCTACGGTTTTATGTAACAAATCACTACTTACGATTCAACTAATATTACCAA
C13_175277364	TGCCTATGCGTATGGGTACATATGTAGAAATAGAAATTAATGTGACTACCCACAACCGTTGGGCAGGAAGAATGTGCAAAAACTGAACAAGTATGGCGATTCTGGGGCACATC<br>CACTGCAGAATTCATTTATATAGATATCAACACTTTGTAGATATCAAACCGGAATTCATATATAAAGTCGATGAAATGTGACTGCTA[C/G]TATAAAAAATTCAAATATGTTGAA<br>CAATAAACTCATTAGATATAATATGCCACAAGTAAACACCAGAAGTAATTCGATACAAATTTCCGATTTTAATTTCTCATCCTACAAAAGTACAAATAGCTCAGACCTACAATA<br>TCTAATGCAGTCGCTCTCCCATATTGCTATCTAGGCAAGAGTGTCCATCATTTCTTATGGGA
C13_175278908	TACAGCCTCTTTCATCCATGAGCCTAAGCAAACCAATGGCTATATCATTATAACCAAACTTGCAAAGCCCTTTAATCATTGTGGAATATGTAACAACGTTAGGTTCACAAATTTT<br>CTGCTTAATGAGCTTCTTGAAGAGTCTCTCTGCCTCAGCAATCCTATTTTCTATGACGAGTCCATCTAAGAGTGTATTAAATATG[A/T]CGATATTTGGGACAACAGCTCGCCTGA<br>AGCAAGAAGCTAGGAGGGCGAAACCGTCATTGGTGCGAGACATCTGACAACAACACTTGATTGCGATACTGATACAGTACTCGTTAACAGGCGCTCCAATGAGACACATTTGT<br>TTAAAAAGGTGGAGAGAAGAAGAAAAATGGTTCATTTTGGCAACAGCATTTAACAACTGA
C13_175280892	TTTACGGCTAAAATTGGATCAAATATCAGCTCGTTTTTCTGCCGAGAAGGGTTTGTTTTGATCAGTTGTTGTTGCAGGTTGTTTGAGATAAGTGAAAAAAGAATTGTTTCAAATCA<br>AATTTTGATCTGTTTCGCTGTACCGTGTGTGCACTTCGGACATTTATCATTGAATTCGAACTGATTTCACAAACACAATCGGAC[G/C]AAGAACTGAGTACTTTGTTTGAAACATA<br>TTACTGATAGAAATCGATGTGTGTGGCTGGGTTGAATTGATAGTTGCAGCTGGTTATAATGAAAGGAAATACGATTTTTATGATAGTTGAAGTGTTGTAAAAAAAATATAATAGA<br>AGCCAGATAAGGTTTGTTGGAAGCCGGAGGCACTGGATGACCGGCAGCCGGTGATGG
S13_216337160	CGCAGCGGTGGTGACGTCGATGGCGGATGAAGAAAATTGCGACCCCCCCCCGGCTGCGGCGCCGCTGGAAGTGGTGGCGGCAGAAATCGGCAGCTCCGGCAGTGGTGATCAG<br>GTAAATCTTCAACAACATCCTCTGTTTCTAATTTCTTTTCTTTCGGATCACATCTTTACGACAGTGGTGATCGAGTCCTTTGTCCTCA[A/G]ATCAGTTCCATACGCAAACACGGT<br>CGAGCCACCATGTTTTCGGACTAATTTACGGCTAAAATTGGATCAAATATCAGCTCGTTTTTCTGCCGAGAAGGGTTTGTTTTGATCAGTTGTTGTTGCAGGTTGTTTGAGATAAG<br>TGAAAAAAGAATTGTTTCAAATCAAATTTTGATCTGTTTCGCTGTACCGTGTGTGCACTT
S13_216342940	AATTTAGGGTGTTAGGGGAGTTTAAGAGGGGAGTTGACGTGGCACACGAGGATTGGTTGGGCGTAAGAGAGGGGACTCACCTCTTAGGTGAGCACCCCTTACACCCTAATATA<br>ATACTATTTGAGCTGATGTATCTCAATGCCTCTGGCTAGAGGATATGGTGATGGTCCTCCAAGGGAGGATGATCCGCCACGTAGGCG[C/T]CACGTCATAGTCCTCCCAAGGATG<br>GTCACTAGAGGGTATGGGAGGATGATCCTACCCCACCACTTTTTTTTATTTATTTTTTAATTTCTATGCCCTATGTACAAGTATTGAAGCCTAATGTACAAATCTCATTCACAAAC<br>AAACAAAGTTGGGCCCACCATGTGGATCGTCCTGGACGTCCATATCTTGACGTTGAAGAC
S13_216344422	ATAAGGGCAATTCTGTCATTCTAGATTACTATAAAGTAATTTATATTAAAGAAAATAAAAAAAAACCTCTTTAAATACCCTGATCAAAACCCTCACATTCCCCATCATCTTTTTC<br>AACTTTGCAAAAGTAATCAAAGATCAAATTTTTATACCATTACCAGCAATAACGAAATACGGCCTTCTTTGATATCCCAAAATCG[G/A]AACAACATCAGTAAGCAAACAGACC<br>CCAAACAGGTTTAATAATCCAAGGAATGTTAGTGATTCCTTGATAAGCCTGTGCTGCAGAAGGTTGAACTTTCTGCACATCTTTCATGTAATACTCTCTACCCACCCTACCAAGA<br>GCTCCACCAAACCCTTGATTTATTCCATAAACCACCACAACCCCACTAACAAGACTCCAA

S13_216346454
AACACATAAAAAGGGTGGACCCACATTAATTTGTTTTTTTATCATTAAGGGTAAGTTTGTCATTTCATATCTACTAGACTGAGAAAACTAATGTCCATTCACTCCAGAGACTATC
CTAGTAACAAACGAGCAAACCACAAAAAGCATACATGTAATTTCCAAAAGTTGGGGACTATATGCATTATTTTACCAAACCACAT[G/A]GAGCGTCCAAGCATTTTACTCTAAA
AATCATAATATAAATGATGTTATATTGTTCCAGACTTCGCTAATAGAAAGAAAGTAAAAATATTTTTGTGTTCTAGAGTTTTATTTAAGGAAATAAAAAAAGGGAAAATAAAAC
ATGTTATGTTCTCAAGTTTCATTTACTAATGAAAGGAAAGGAAAAGAAATAAAAAGAAAAT

S13_216347527
TTTGCACTCCTAGCTTCTTGCTTTAGGCGAGCTGTTGTCCCAAATGTCTTCATATTTAGTACACTCTTAGATGGACTCATTCGTGAAGATAGGATTATTGAGGCTGAGAGACTCTT
CAAGAAGCTCATCAAACAGAAACTATGTGAACCTGATGTAGTTATGTATTCCACTATGATCAAAGGGCTTTGCAAGTTTGGCAA[C/T]AATGATATTGCCATTGCTTTGCTTAGG
CTCATGGATGAAAGAGGCTGTAAGCCTAATATTGTTGTATATAGTACCATCATTGATAGTCTTTGCAATGACAAAATGATAGAGGCTGCTTTCAAGCTTTTCAATGAAATGGTAT
TTGCCAAAGGCATTCAACCAGATGTCATCACATACACCTCTCTGATTTATGGCCTTTG

S13_216353986
CACAATGACTCAATTAAAAAACGCAGATCGTAAAAATACAATTTAGTTTTTTTTACATAGATTGAAGTCGATTTCTTCAACGATTGACGAAATTTGAATGCTAGAAACACTCATC
AGCGATTGATCGAACGAATCGAGTGATTCGCTTCAAAATCACGGGAAAAATGAGATATTGTATGAAATTAAACTGGGTTTTCTTT[C/A]AAAAAAAGTTGAAGAACATATTGATT
GGGTGTTTGAATCATTGACTGGTGATAAAAATCGTACTATAATATAGTGATTATTGAGATAGAAAGTGAAGAAAGTTGAATATAGTGGGTTTTGAAATTACAGGGGAGAAGATT
GGATAAATTGACTGAAATACCCTTCTCCTTCATTTTTAAATTTTGACAAATATTATAATT

S13_216357087
CTAGTTATTGTTGGTTTGAGGTATTTGTACTTTTGTAGGATAAGAAATTAAAATTGGAAATTTGTATCGAATTAGTTTTGGTCTTTACTTGTGGTATATTCTATCGAATGAGTTTAT
TGTTCAACATATTTGTATTTTTAATACTAGCAGTCGCATTTCATCGACATTATATATGAATTCCGGTTTGATATCTACAAAGT[T/G]GATATCACAGCGTTGATCACAAGCAATGT
TTTGTAAAAATCAGTCCTGTATTAGAACATTACTGAATTCTGCAGTGGATGTGCCCCAGAATCGCCATACTTTACAAGATACATCAGATTTTCATATAATTAGGATTTCCATTGT
CATCAATAGACAACCACCACCTGTGTGACAACATAAAAAAAGTAATCGAAAATAAA

S13_216357346
TTACTGAATTCTGCAGTGGATGTGCCCCAGAATCGCCATACTTTACAAGATACATCAGATTTTCATATAATTAGGATTTCCATTGTCATCAATAGACAACCACCACCTGTGTGAC
AACATAAAAAAAGTAATCGAAAATAAAGTTTTTTTTAAAGGGCCAAGGGTTACAGGACCTGTTAAGATTCTCATAATAGCATAAA[A/C]AGCTTACGGTGCACATGTTCTTCACT
TGAACCTGTATGGGTATGTCCTTCATGATCATGGAAAATAAGTGTCAAGATTCCAAATTAACAGAGTACAGATCCTACTAGAAGCTTGTCATAACGTTCCATCCGGTGAAACTT
GAGCTGACTAAAAACATAAATCATCCCTTTTTGTAAGTGTGGAATACTTTGCTGTATTTA

S13_216358901
CCGTATCTGTGCAATCCGTTCAGTAGCATCAACTACCATTTCTGGACCTGTGATTTGACTATCCCCCACCTCTGCCCAACAGAGAGGTGACCGGCATTTACGTCCGTACAATGCC
TCGAATGGAGCGGCTTGAATGCTGGTGTGGTAACTGTTATTGTACGAGAACTCCACCAAAGGGAGATGTTTTTCCCAGCTGTTGC[T/C]GAAATCGATAACACATGCCCGAAGCA
TGTCTTCTAGAGTCTGGATAGTGCGCTCAGACTGCCCATCCGTCTGAGGGTGATAAGCTGTGCTCATGTCTAATCGTGAGCCAAAAGATTTGTGCATTGCTTGCCACAGTTCTGA
AGTGAATCGTGCATCCCGATCCGAAATAATAGAGGTTGGCACTCCGTGCCTCGAAACCA

S13_216360386
TTTGATGAACCTTCTGTAATAGCCTGCCAAACCCAAAAATTGGCGTATTTCCATTGGTGTACGTGGTGCAGGCCAGTTCTTAATCGAGTCTACCTTGGATGGATCAACATGAATC
CCATCCTTGTTTACCACATGGCCTAGAAAGTGGACTTCACGAAGCCAGAAGTCGCATTTTGAAAACTTGGCGTACAGCTGTTCTG[T/A]TCGAAGAAGTTCCAAGATAAGTCGTA
AATGTTGTTCGTGTTCCTCCTGACTCTTGGAGTAGATCAGAATGTCGTCGATGAAAACGATGACGAACTTGTCTAGATAAGGCTTGCACACTCTGTTCATAAGATCCATAAAGAC
TGCAGGTGCGTTTGTTAACCCGAATGGCATGACTAGAAACTCGTAGTGGCCGTAGCGAG

S13_216361268
AAGTGCCATAATGGCAGTGTGACCCTTTCGCAAACATTTCTGAGCCTTCAAAAAGGAGATGATGCCAACCACGGCACCACTCTTGTCGCCTTGAACTTCGAGAGTTCTTTACCA
GAATGGGGAATACAAACTATCTTTTCCTTGCATAAGATCTCTGCTTGCTGTTGGGATAACCAATCCATACCAATGACGATGTCGAA[A/G]CTACCTAAAGCTATGGGAATGAGAT
CGATAGAGAAAGTCTGACCAACGAGGATAAGATTACAACCCTGAACTATGTGTGTGGCCTCTAGACTTTTACCGTTGGCTAACTCTACGACATGTTTGGTGTTTAAGGGAGTTG
GTGTACGTTTTAACATTTGACTAACTTTCAAAGACATATAACTGGTATCTGCAGAATCAAA

S13_216376422
CAGTGGTACGCAGGGGTTTACGTAGGTTTTACGTAAGTGCCCTTCATGACCCGAGGCAGTAGTAAGCATAAGGTTACGTAGGTTTTACGTAAGTGTCCCTCGAACCCTGAGGAC
AGTAGTAAGCGCATGTTTACGTAGGTTTTACGTAAGGATCCTTCCCAACCGAGGACGATGGTAGATAGTCTAGTAACAGTGTAAGT[C/A]CAAGTAGTTGTACAATCCCATTCCT
TCAATCCCATTCCCTACCCACCGGGAATCCCATGCCTTGGTAAGGGTGTGAACTCACCTTGGTTTGCTCGGTAGATACACAGAAAGGTTACTTGAGCTAAGGGTCAACCACGTC
CTAACATGGTTACCATACAAGTCAGGTTTTGGTTTCAAGTAATGCACGTATGTTTACGCAT

S13_216376492 AGGTTACGTAGGTTTTACGTAAGTGTCCCTCGAACCCTGAGGACAGTAGTAAGCGCATGTTTACGTAGGTTTTACGTAAGGATCCTTCCCAACCGAGGACGATGGTAGATAGTC
TAGTAACAGTGTAAGTCCAAGTAGTTGTACAATCCCATTCCTTCAATCCCATTCCCTACCCACCGGGAATCCCATGCCTTGGTAAG[G/A]GTGTGAACTCACCTTGGTTTGCTCG
GTAGATACACAGAAAGGTTACTTGAGCTAAGGGTCAACCACGTCCTAACATGGTTACCATACAAGTCAGGTTTTGGTTTCAAGTAATGCACGTATGTTTACGCATAACTAACAG
GTTTCGAGCACTTATGGATCATGGCAAACACATAACAGTCACGAGCAATACACATTCATAC

S13_216377612 ACAGCCGATTATCATTCAATCGGTTCTAGATCATTAGCACATAATTATCATTCTACATAATTCAAAACATGTTACACATATGAACCGATTTCATGAACATCATACTTCATCAGTT
TTCATTACAATCAAATGCATACCATCTCTAAATCATATGTAAAACATATAAGTCAACACATCGTAAATCATGAAATCAAAAGTAT[C/G]ACACTAACCGGTTAAAGAATGGAAC
AAGGATTGATAAAGCTTCGAGAGAGATGAGATGTGGCTGCCGTCGAGTTTGAGAGGAGAGAAAGAGAGTAGGGTTTGTAAACTTGTGTAATTAGGTAAAGTGTGGGATTTATAC
CAACACCCTAGGTGTTGTGCGAGTTGATGAAGTGGGCCGAACCCTCCGAATGGGCTGCCCT

S13_216378430 ATTCAAGGTATTCTAGGATGACGTCATCATGTGTTCTTGAAGAACTTCATGTTTTGGCCCCAATCCACCAAGAATAACTTGGATCTAACCGATTTCCACATAAACAAACAAAGAT
CCTTCACAGATCTAAACATATTCACGGTGAAAGGGATTGAAAGATGGTTTTTCCAGCTTTCTTTCAACTCTTTTACACTCAATGC[T/C]TTCAAAACCGATAGAAACGGAACTTGT
GCCGACTTACTAATCATTCCGGTGGTCGAGTGGCCCAAGATCCGGATTCTATCCACGAGGTTCACCGTTTTCGGGTTAAACGTTAAACATCGTCCCGAACCGTTCACCAACCGG
ATTTGGGTGATTCCTATCCGATCAGGAGAACCAAGTAAAGACGAGGTTTCCGTTGTTTG

S13_216380099 TAGAGTCCAACAAATAGTTGTCTGTACAAAGTGAATGAAGGGTCTCTAGAAGCAGATAAACAATTGCTATATTTAAATCTGAGTTGTCGGAACAAAATTAATTGCCTATATTTTT
TGTCTGTAATAATTTGTTGACTGTTTGAGGCATGGTATGGGACATGTTATTTTAAATTAAATAGTAATGATAATTGTTATGGAAA[G/C]TTCTGGACAATCTGTTTCGCTCAGTGC
CATGCCCCGATGATTCCGCCATCGGTTGGGGTGTGACAGATTGGTATCAGAGCCATAACTATAGGGAATTAGGCAAGACTCGACCTAGTCCGGGTCGATGTCTTAGAGACCTAG
TCTATAGTTAGGACCCAATAGACCGACTCGTGCATGTCCATTAGGGATTATGTATGAGC

S13_216380508 AATATTCGCCATTCTCATCTACACTACACTGTTACTGCACTCGAATTTTCATATAAAGACAAGTGGTTAAGTCGAGAATAGGTGTGAAAACCGCAAACTCTCGACCATAATCTG
CTAAATCGACCCGCGCTTTTACTTACCAACGGGGAGAAGTATACCAAATCAGGAGTGAAATCCATATTTTGATGAATAATCTCCTC[T/A]ATTTATTAAAATCAAAGGGAAGTTG
TCAAGCCAGGGGTGAAACCCTAACCTTGACGGCTTATCCATGGTTTTTACAAAACTCTCACCAAAGTCACGACGGACTCCAACGACCTGAACGCACGATATGACAAGAGGGAT
ATGCGCCGGATGCCCAAGATTCGAGGCAGAGGCCCAATCCTGAAAGTCAAAATGTGCACACC

S13_216381220 TCGAACGACACGCGAACTTCGGATGATAGGTTTCTGTATTCTGACTGCCTCTGTGACTAAATGCGTATGTGTTTATGTGCTTCTGTGCCCTATGTGCTTACTTGCTTACGTGCTTA
TGTGCTACCATGATTACGTGATTCAATGATTTCGTGCTTATGTGTTTAAGTAATTATGCGACTGTGTTCCTGAGCTTTAGTAAG[T/C]GTTAGACATTTGAGGTGAGATTCGGTTAT
GACGTGTCTGAGACCTACGACAATGTCTATTGCAGACAATGTCAACTGGACCATCATCAGGATCCCGTCACCCACTTACTCGTTAAGAAAAGAGAGACAGGCGTCTTACTGCTA
TCATCGCCAAGCAAGTAGCAAAAGCTGTGAGTGATGTCTATGAGAACGCAGCAAGTC

S13_216382190 TAAAACTGGTTTTTGGGATAAGCCCCCCCAAGTCTCTGCATCAAGCTACCACTCCACCAACCGCTGAAACCGCCACCGCTCAACCGTCAAAGTCATCACGTCGCAAGAAGAAG
AAGAACAACAACAGCTCCAGCAACAAGAACTGTGCTGTCACTACCACTGTTGCTCCTCTCCAAGCCGTACCGGCTCAGCAGCAATCT[C/T]ATCACCGACCAGCACCAGTTACA
TATGCACCGCCAGCAAAGCGTGCATACACAGGCCCCCACCCGGTTTGCCCAGCATGCCCATATCATCATCTGGTGGGTCTTGCCTATCGGTTTTGCGCTCACAGCAACATGTAC
GGCCATTTCACTGCTAATTGCCGTACTGGTCCTCGCCAAGCCCCAGTTCAAGCCACTGCTCAT

S13_216383457 AATGCGTGAACTCTCGAACCAGCTCCAATAATTACTTGAGAAAGGTTTCATTTGCCCGAGCACCTCTCCTTGGGGGCGCGCTAGTCCTCTTCGTTAAAAAGAAGGATGGGTCGT
TCAGGATGTGCATCGACTATCGGGAGTTGAATAAGTTAACCATCAAGAACCGCTATCCTCTGCCTCATATCGATGATTTGTTTGAT[T/C]AACTGCAAGGTGCTACGTGTTTCTCG
AAGATCGATTTACGCTCAGGCTATCATCAACTACGCATTCAGGAAGAAGATATACCCAAAACCGCTTTTCGCACTCGCTACGGCCATTATGAGTTCATAGTCATGCATTTTGGTT
TAACAAACGCACCTGCGGTTTTCATGGATCTGATGAGTCGTGTGTGTAAGCCATTTCTA

S13_216383878 CATCGAGATGTCTTGATCTATTCCAAATCGAAAGCCGAACACGCGCAACATCTACGTTTGGTTCTCAAGCTACTCCAGGGGAATCAACTCTATGCCAAGTTCTCTAAGTGCGAA
TTCTGGCTAGAAGAGGTTCAGTTTCTGGGTCACATCGTTAATAGTCAAGGTATTCATGTCGATCCCGCGAAGATTGAAGCAGTTAA[A/G]AGTTGGATTACGCCTAAGAACCCGT
CTGAAGTCCGTTCTTTTCTCGGATTAGTGGGCTATTATCGACGATTTATCGAAGGATTCTCAAAAATCTCTGTGCCGATTACTGCTCTTACGCATAAAGACAAGCCTTTTGTTTGG
GGAACCGAACAAGAGTCTGCTTTCCAAACCCTTAAGCACATGCTTTGCAATGCTCCTGT

S13_216385258	GTTTACCTCGCGATTGTGGGAAACGTTTCAAGCGGCTCTCGGTACGACGCTTAACTTAAGTACCGCATTCCACCCCCAAACCGACGGACAGACTGAAAGAACGATTCGTACTCT TGAAGACATGCTCCGCTCGTGTGTCATAGACTTCGGTGGTAATTGGGATAAATACCTGCCGTTAGTCGAATTCTCGTACAACAACA[G/A]TTGTCATACCAGCATCCAAATGACA CCATTCGAGGCTTTATACGGAAGAAGATGTCGATCGCCTATTGTATGGCACGAGATTGGTCACTCGCAATTAACTGGTCCTGAGCTACTGCAAGAAACGACTGACAAAGTCCTA CAAATCCGAGACAACTTGTTGAAAGCTCGAAGTAGACAGAAAAGTTACGCCGATAGACGAC
S13_216386547	ATCGAAATCGAATTATGATAATTAGTGGAGAAGAGATAACACGAGATATGAATAGTTGGGATTAAAAGTAATTTGACTAGGAAACTCCTTCGCATCGCACCGCTCGCAATCGAA ATCAAAATATCAAAAATCGTCGCACCAAACACTCGAATCAGGCAACTGATCGATCAGGCTACCAGCCGATCGACCAGCCGGCCGAT[C/T]GGACTGCCGTCCGATCAGACAGG CTGTCCGATCGAGCTGCCTGGCCGATCGGCCACCCATTTCCTCTTTTAGCATCCTATAAATACCTCTTGTCACTCTCCAACTTACGACTTTTGGCAAGTGACGTCCGACCAGCTC GTGCTCCTCACCTTTTCTCAGATTTCTCTCGACTCCGGTAAGATCTCGTCCTAAATCTTGTA
S13_216386718	GCTACCAGCCGATCGACCAGCCGGCCGATCGGACTGCCGTCCGATCAGACAGGCTGTCCGATCGAGCTGCCTGGCCGATCGGCCACCCATTTCCTCTTTTAGCATCCTATAAAT ACCTCTTGTCACTCTCCAACTTACGACTTTTGGCAAGTGACGTCCGACCAGCTCGTGCTCCTCACCTTTTCTCAGATTTCTCTCGA[C/T]TCCGGTAAGATCTCGTCCTAAATCTT GTACTTTCTTGATCTACACGCACTCCTACACCTTTCTATCTTTTAAATCTTAACTTTTAACCGTGAAATCATCAAGATTCAAGGTATTCTAGGATGACGTAATCATTTGTTCTTGA AGAACTTCATGTTTTGGCCTCAATCCACCAAGAATAACTTGGATCTAACCGATTTCCA
S13_216387607	TGTTTATGTGATTTTAGCAGCGGAAATTTTAACATCAGGATCGTTGTAAAGCTAAAACTTTTTCCGATTATTTTTATTTTACAACTCGGGATAAAACCCCGATAATTTACAATACA TAAGTTTTAGTGAATAAAATTTATTTCAAAACATATTTATTTATTTTATATTGAGCCACTATTTTAAGCTTGAAATGCTCCTCA[G/A]CACTTTTCCTGATTTACAGCAGATCACCT GAAACATGTTTGAAAAAGATTTTGTCAGCGGGGAAATACTGAGTGAATCATTCATTTTACTGAAAACGACACATTTTATGATTATTCACAGTGTTAAGATCTTTTACGCATGTTT CTGATATCAACCAACTACCCACAGTATTTGTCACTCGACCGGATCTGTGACAGTGG
S13_216387959	CAACCAACTACCCACAGTATTTGTCACTCGACCGGATCTGTGACAGTGGTCATATCACCATTGGCTGCCCCAATGAATGACGTAAATCATTTAAATTAGGTTCGCCCACCAAAA ATGATTTAAACTGTAGTAATGTGCACAATACCCCACATACTGGCTGTAATTTGGTGATTACATCAACTTAATCACTGTAATTATAA[T/C]TCTGAAAATGATTTGGAGTATTGTAA AACAGTTAATAAAAAGAGAATGACTCACATTGCAGATTTAGTACTGATAGAATAAGAGTTTAGCCCTGTTAACCTAATTTCACAATAATGCACACAAAACAGGGTTAGTGATCA ATACAGTAGTTACGATAACTCACGAGATCAAATTCTCACAATGAACGACAAAGTGCTATA
S13_216388326	ATCAAATTCTCACAATGAACGACAAAGTGCTATACTTCAACTCATTTATCGATTTAACGACAAACGACAAAGTATCACCCTAATGTGGGCAGCACTTAAACATTCTTTGGATAT ATTGATCCCGGAAAAATGAATCGTAATAGCGATCGAGTAATTACCCTGATTGCGGCAGCGTTTCGTGATCGTGTGTGTTTGATTGT[G/A]GTATAACAGTTGGAACTCGACGCAC AAACAGAATTTCTACGTCGTTCTAATACCAGATTTCAAGTGCCAAGGTCGGCTATTTATAGCCCGAATTTCACGTCGCTTACGGACCGTATGACCTATCCCTTACGGTCCCTAAA GGAACCGGTTGGCTTACGGTCCGTAAGGACTAACCCTTACGGTCCGTAAAGGAGCGACCT
S13_216389204	TGTCCGATCGAGTGACATGTTGCTGAAAACATGTTCTTCGCTGGAGTACTAGCCGATCGGGTTGCCGGCCGATCGAACGATCGTCCGATCGACCGACCGGAAAGGTAAAGGTA CTTCAATGTTTTCAAATGCTGCAACAAAAACTTCAAAAGTCAAACCATCATACACAAACACATCTTACTCAAAGGAAGAAACAATCC[A/G]CTCGAACAGCCATCCGATCGGAC TACCGTCCGACCAGATAGCTGTCCGAACGGATTGTCAACCTCACGGTCAGCTGTCCGATCCATCAGCACTTGTTTCCGTTTTACGCGTACTTATCATTATGCTATTGAACCATTC AGGCTAACCCTACTCTCAAGCGCTCCCTTCAATCCATCAACCACTGTGAGTATTCTCGAACC
S13_216389559	CAAGCGCTCCCTTCAATCCATCAACCACTGTGAGTATTCTCGAACCCTTTTTGCTTTCGCACTTTTGGGTGTTACATACGTTACTTATTCTAAATCACAATCGAACACACTACGCA ATACATTTAAACGCTAACTGTTATCGCATGTATACATGACTTAATGAATTCCTGTTTGTTATGTTTACACATGGAATGCTGTCT[A/G]CCTGCCTTAACAACGATAGTACTATAGT TTGGACTCAGCACCCGCTCATGCGGGGGTTGTTAAGGACAATTATTTGCATGGATTACAGTGGTGATCATGTATTACGAACTGCCTCGGGCAGTCAACCCGCAGTCATTGGTAT CGATAGGTTCATGTCGATAACTAACATGCTTCGTTTTCCTCTGTGTACGTGTTGGTTA
S13_216392091	GTTTTTGAAAACAAAGGTTAAATGTTTGTTCACTTTTGGTCAATCAGATGTTTGTTTTTCCTTTTGTAATCTCTGTTTTTGAAACAGAGGTTAGGATTTCAAACAGTAGTATGAGA AACATATGTTTGAAACCTCTGTTTGGTTATAAATAGAGTTTTAAGAAACAGTGATTTGGCGTAACAGAGATTACCTTTTACAAA[T/C]AGTGGTTGACTTTAGTCAAACAGACTTT TAGATACCTCAAACAGAGTTTTTAATATTAATCAAACAAAAAACTAAAAAAAAATAGAATATCTATACACCTATTTATACTTTCTATTAAAATATGTTATGCATGGTTTCCTAAG AAACGACCCGTGTTTCAATATGATCTGAAGATTTGTCTGTCATTTCGTTGTCGCCCT

S13_216392229	TTGGTTATAAATAGAGTTTTAAGAAACAGTGATTTGGCGTAACAGAGATTACCTTTTACAAATAGTGGTTGACTTTAGTCAAACAGACTTTTAGATACCTCAAACAGAGTTTTTA<br>ATATTAATCAAACAAAAAACTAAAAAAAAAATAGAATATCTATACACCTATTTATACTTTCTATTAAAATATGTTATGCATGGTTT[C/T]CTAAGAAACGACCCGTGTTTCAATATG<br>ATCTGAAGATTTGTCTGTCATTTCGTTGTCGCCCTTCGAAGAACGGAGATGGAGCGGTAAATGAGTGATTAAAATTTGGGGTCGTATTTGTTATAAGGGTTTAATGGGCCTTCAA<br>AGCCTAACCCATTATTAAGGTTAATTGGATCAAATGTAAGGGCTTTTTTATATTGTTC

Supplementary Table S3b Sequences of SNPs selected from the HA-R9 whole-genome resequence for R_{11} in the present study

SNP	Target sequence
C13_181780331	TGAGTGAACTAAGTCTTGTAGAGAGCGTATTTTCAGTTTGGTCAAGGGGCTGTAACCGTGTCCACTGAAATACAAGAGAAAACTTTGATATCTCGTGTGTCTACCTTTCTCTTTG<br>TAGCTTGTGTTCTCATATAAACTACCGGTTTGCATTCTAGCTTGGATTCCGCACTTGCTAGTGTGTTAAACATAACAAGGATAAG[A/G]TTTAACCTCAACCTCCGGGGGACCTAC<br>AAGTGGTATCAGAGCCGTGGCTCTTTTCCTTGTTAAAAAAAAAAAACCGAGTTTATACAAGACTTTGGAATGTTTGAACAAAAACTCACATGGTTTAGCACCCGTTTTTAGTGTT<br>TTTCTCGCATTTCTTGACGTAAAAACGTGTTCTAAACTAACCGGGTGTGTTAAAGGAC
C13_181780894	CACCTATTTATAGCCGAAGTAGTGAAGGAGGAGATGTGCTGATGGGCCTGGAGTCGACAACAAGGAATATCCTTCTTGTCCCCTCTGCAGTAACCGTTAGTGCTTCTAGGTGAG<br>GACGGAGTTGGCGCACGTTGAATGGATCCACGTGTCCTGACCGTTGTCCTTGTTGTCTCTTTGTCATCAGCGGCTGAGTGGAGATC[G/A]TGGAGCAGGTGTCGACGCATGCTGA<br>TTGGTGCCACGTATGCGTCCTTGTGTACTCTCCTGTCTCCTGTATGATTGTTAATCGTGGAGCACGATCGAATACAGCCTGTTGGACGCTCATTGGTCCACTTCACTGCCACTCGT<br>ACCTTTAGTATTTGTCCTATGACGACCTGCGCTTCTGTCCTCCGCGCGGCCCATGGGGG
C13_181781392	GGTTTCACTTGACTAGTCGGCGCAGGGCTGGGAAAGATGCCAGATAAGCATAATAGGAGTATGGTCTCGCGCGCAACCTGATTGGTCTGCGCGGCTTTTTGGGACAATACCCCT<br>TCAAATCACATTTTCCCACATATAAAACATGATAAGGGTGCTTGGTGTGGTAAGGTAAAACCATACGAGTATCGAAATCTTGTCCT[G/A]GGTTGCGTTGCTTTCGTTGGGTACC<br>GAGGGGAAACAAAAACTAAGGGCTTCCATGACTTTATTATATTTTAATTTAATTAATTAAATATCATATTTGTATCATAGGATTGTAGCGGTTTGGGTACCTTTGTTACTAGTAAA<br>CTCGATTGTTACCTTTGTTACAATAAAACACAAGTCCAAATCAAGAGTTACTTCTTAGA
C13_181783295	TAAGAAATTTGTGATCACATGGACCATTTTGTTTTTGTACCTCACAATAGAGACCGAGATTATCAGCATGCTTGACACAATCATTATCTAGGGACATTCTTCATTTCACATCACA<br>TTTTCAACTGACGATATAAATCTATAAAAGTCTATTAACTTCACTTAGGCCATTGGTCGTGGTGCGTGATGGGGCAATCATCACG[C/T]GTGGAAGCATGGAACTTACCACCGCC<br>ACCAACTAATTCCACGAGTGATGATTAAGGGGGTGTTTGTTTTTTGCAAGAAGTACTTCCTGACCTCTTATGTCTGCGCCGCGCAGACCACGCAAAGACCTTTGGGTCTGCAGCG<br>TGTTTGTTTTTTGGAAGACATTTCACTAAAAAACCTCTTCTTGATGTCTTCCTCGTGCA
C13_181784617	TTGGGGGGTGTTGTGAGTGATGAGTGAGTGATGGGTGATGACCACCACCACCCCCCTTACATTGTTTATTTAGCTATCTCGTAACTTTAATACGTCTATATAAACAAATTTCAAA<br>TTCATAATAAAAGAAAATGTTCTCAGATACAGTGGCGGGGCTTGAACAAATATATAGGGGGCCAAACTCAAGAAAATCAAGGGGC[C/A]AGGAATTTTAGGCAAAATCAATTTG<br>GGGCCGGACTCAATACGTATATACAATTTCTGACGAAATACGCGTAAATTTACACTGCTTTGCAAAGGGGGGGGGGGGCGAGCACCCCCGGGCCTCTTAAAAGCTTCACCTAT<br>GCTCAGATATTCATACTTTCGACTAGGGGTGCAAACGAGCCGAGCGGCTCGCGAGCTACTCG
C13_181786257	GTTTTCGCCTAAAAATTACACTTAACTTTTTTTAAGGGGAGGGGGCGCCCCTACACCAAATACAGATCCGCCACTATTCTCGGTAATATGAAACATCTTGATAGAAAAAAAAAA<br>CCATATTTGCTTAAGTTTCTTAAATTCAGCATATCAACATTATCGTTCAAGTATTTTATGCAACTAATTACAAAGTATTTAGACAT[G/C]AGTTAGGCCTTTAACTAAGTACTTAA<br>ATTCCTCTCATGCCTTAGGTAGACACACATCATTGGTTCAAATGACCAATAATCAATTTACTCAAATTATTTGTGGCCTCCCTACATATTCCTAATGACATACAATTATTTGTGGC<br>CTCCCTACCTACATATTCCTAATGACATACAATTATTTGTGGTCTCCCTACATATTCC
C13_181787450	TTTACTTTTGTTTTAACCTCATTGTCCTCCATAACTCGCCTTATCCCACGTTCTATCTCCTCAGCTGTCACAATAATTGTGTCAGCCCTAGGATTAGACACATTCTTCCTATAATCC<br>AATTTTATCTCCACCGCCAATCCCAACTCCACCACCATTTCAAACGCATTCATATGTTGCTCGGCGTACATCGGCCATGTTGC[C/T]AATGGTACACCGAACCATAAACTCTCCA<br>ACAATGAGTTCCACCCACAGTGGGACACAAACCCTCCAACTGCATCATGACCCAACACCGCCGCCTGTGGGGCCCACCCAATCACCTTCCCTATTCCACATGTTCGCTCCAAAA<br>ACCCTTCTGGCAGTACCAAACTCGGATCCTCATAATCACTTGAGATCCTTGATGTTTG

C13_181788711 GACGTTGATCGCGATTCACAAGTAATTTTGCGATCTCAACGGTTGACATAATGTGACCAACCCCTGGGGAAGGGATGAATATTAGCTCTAAAACTTTACTAGCCATATTTGTTTT
GGGTTCATGAATGGTTTGTTTGGAGTCTTATACATACATATATTCAGGTTATATACCATTACCTGTTGATGGTATTGCATTGCTG[G/A]CGAAAATCAGGTGGAAAGTTAGGCCCA
CATGGCCCATTTTATTATAGTGTTTCACTGAATTATATAAGCAAATATCCTCGAAATGAGCAGTTTTCAAATCAGGTGGCTCAAACAAGGGTCCATGAAAACTAAGTACTTAAAT
TTCTTTCATCCCATGGGGCACACATATCCTAGGTTGAACTGACCAATACTTTAAATTA

C13_181789832 TGTAGTGTGCGAGAACTTTTTCCAAAAACCTTCCAATTTTTGTTGATTGCCTTTTTTCTGGTTAACACTACAATGCTCATACGTCATCGCTAACGCCTCTTCTTGTTTCTTCGTCCA
TCTTTGCGGATCCGCCTTACCCCTCTTTCCTTTCCGTTCATTTTCGGCTTCTTTATTTTCGTCTTCATTTAACTCTTGAGTCT[C/T]GGCTACAAATTCTTCATCTTCGTCATCATCGT
ATATTGGAAGCATGTGTTTAACATTATCTCTTGGAGATTGAGTCGGTGGACTCCGATAAGCAAACGGGTCCAAATCGTTTTGGGCTTCCGTAGGATACTCGTACACGCCCGGTGC
AACCATCGTGGGACCTTGTGATAATGAAAAAAAATTGTGGTGGATAGTTGGAT

C13_181790141 GTTTTGGGCTTCCGTAGGATACTCGTACACGCCCGGTGCAACCATCGTGGGACCTTGTGATAATGAAAAAAAATTGTGGTGGATAGTTGGATTGGGGTTGGGTGTTGTAAAAAC
CGGATTGTTTGGGCGTAAAAATATGGAGGTTGAAAGGGTGGGGAGTAACCGTGTTGTGATTGAAATGGCACACTTGAACCACTCGA[T/G]CCACCTCGTCTACTTTGCGAGCCGT
CCCCCTCGCCTTGGAACCGGAACTCGTAACTTTTTTCTTGTCATCGCCCTTGTTTTGTCGGTCCATAGTATGCTTTGAGAGTATGTGAATTGGGTGAAAAATGGAATGTAAAATG
GTAATATTTATAATGTATTTAATTATTTTTTTTAATTTAAACTGCACTATAGCCGTTAAC

C13_181790455 ATTGGGTGAAAAATGGAATGTAAAATGGTAATATTTATAATGTATTTAATTATTTTTTTTAATTTAAACTGCACTATAGCCGTTAACATCATGTCCAATGGTCCTTTTCCATGCCT
AACGGCTATATTGTGAAGCGGTGAAGAGTCCCCGATTTTGAAGCCTGTCGCGTAGTGATAGGGGACCATGGCGGTGTTCCCCGC[G/GC]CGGTGAAGTTCACCGCCCTTCACCC
CTCACGCCCCGTTCACCCTAATAAGAAAACCAACAACAAAAACATATGGATGCCGATCTACTCTTCTCACTCATCTCTTTTACTTCTTTTACTTTTAAGCTAATTGTTTATCCTTT
GTCATCCATCTTTTATTAGTTTATATCTTCTTAGTTATCCAAGATTTCTCTCTTTGATA

C13_181790676 CACCCCTCACGCCCCGTTCACCCTAATAAGAAAACCAACAACAAAAACATATGGATGCCGATCTACTCTTCTCACTCATCTCTTTTACTTCTTTTACTTTTAAGCTAATTGTTTAT
CCTTTGTCATCCATCTTTTATTAGTTTATATCTTCTTAGTTATCCAAGATTTCTCTCTTTGATATTAAATGTAATATTAAATTT[T/C]AAAGTTTCACACTCTTGGTTCATTTTGGTTT
TAATGATATATATATTAAATGATGAATTTTAACTATTAAAGTTATCCAAGATTTCTCTCTTTGATATTAAATGTAATATTAAATTTCAAAGTGTCACACTCTTGGTCCATTTTGGTT
TTAATGATATATGTGGGTTTCCACTCTCCTAGCTAGATCTTATCCTACCTTA

C13_181791091 AAGTGAACCATCCTGGTTTTAAACCCAAATCGAACCCCATTTTGAAACCAATTAGGTTGAACCCTAACCGAACTGTAGAGTTGAAATCGGTTTGTAACCCTATGCTTAAAAATC
AGTTCGAACCTACTTAAACCTGAACCAAGCCAGTACCCAATTTCAAGCTGGTCAAACCCGTACTGAACCAGTACCCGGTCAAACCC[A/G]AGCCTTTCCCCTAAAAAACCAGGT
CAGAACCCGAATCTATTTCACCAAAAACCAGGTTTGACTTTCTTTGGGCCAACCCGACCAGGTTTCAACCGAACCAGTTTGAGCCACAAACCTGATTTGCGCACCTCTACTTCT
CCTAGGTACCAACTGGAAATACATCTACTGCCAAGTCTGCCATACAAAACGTACCGGCGTAG

C13_181791896 TTCACTCTTACTCTAACTTCGTTATCCTCCATTACTCGTCTCATCCCACTTTCTATCTCCCCAGCTGTCACATTAATGATCTTATTAGCCTTGGGATTAAACGGATTAAACACATC
CTTCTCATAATCCAACTTTATCTCCACTGCCAATCCCAGGTCCACCACCATTTCAAACGCATTCATCTGTTGCTCAGCGTACAT[C/T]GGCCATGTTGCCGATGGTACACCAAACC
ATAAACTCTCCAACAATGAATTCCACCCGCAGTGGGACACAAACCCTCCAACTGCATCATGAGCCAACACCGCCACCTGTGGGGCCCACCCGATTACTTTCCCTATTCCACGTG
TCCGATCCAAAAACCCTTCTGGCAGTACCACACTCGGATCCTCATAGTCGCTTGGGAT

C13_181792157 TGGGACACAAACCCTCCAACTGCATCATGAGCCAACACCGCCACCTGTGGGGCCCACCCGATTACTTTCCCTATTCCACGTGTCCGATCCAAAAACCCTTCTGGCAGTACCACA
CTCGGATCCTCATAGTCGCTTGGGATCCTCGTAGTCGTTTGTTCTGACGGAGGTCGACGTAGGGACCACAAGAAACGGTGACCACT[C/T]TGCTCTAAACCACGTGCTATCTCCT
TCACTTGGGCCTCATCAAAACTTCCCATACTCCCAAAACACAAAAACACAACCGAGGAAGGTGGTTGACTATCCAACCACCTAATGACGTCATCGTCAAAAGGTTTCCCACCA
CCAGAACCGCCTTCTAAGTTGAGTATGGGTCCCACCGGATACACCGGTGGGATACTAATGTC

C13_181792447 TTGACTATCCAACCACCTAATGACGTCATCGTCAAAAGGTTTCCCACCACCAGAACCGCCTTCTAAGTTGAGTATGGGTCCCACCGGATACACCGGTGGGATACTAATGTCAGC
AGACAACGACTCTATTGCGTGTGTTTCCAGATCCAAGAATGTATTTACAATGATTCCTTTGGCCTCTCCAAGTTTCTTTGCAAGCC[G/T]CTGAACAGAGTCCAGTCCTTCTCTAG
TCTTGACAAGACTCCAAAACACTTTAGTTGGCACCGGTTTGACAAAACTTGGAACCGATATCTCGGTGTCTGAGTTGCTCAGCTCAACAACGTCTCTATTCAGATCATCACAGA
GTAGTTTTATAAATAATGTAAATCCAAGAAAGGCAGCACTAGAAGTGAAGAAAACGAAGG

C13_181792863	CATTAGCCACATCAATCATGCTGGTGCAAAACATGTCGACGACAAACCCAACAACCTGACATGAACCCGATTGACTTCTCAAATCAACTACTGCATTTCTAACATATTTGGAGT<br>GACTTCTGATGAACTCCGTTGGGCCGCCATGAGTTGGTGAAGATTCAACTTGAGGGAGTTGAATGAAGGATATGCGATCCATGGTG[G/T]TTTCAGTTAATGATTCTATGTAGGT<br>GGTGATAGCCGAGCCAGAAGTCATGCCAGGAGGCTTGATGACAAGGACGGTTATCGCGAGACGTTGATCACGATTCACAAGTAACTTTGCGAGCTCTATGGTTGACATGATATG<br>ACCAAGCCCAGGGGTAGGGATGAATATTAGCTCTGCAACTGCATTCGCCATATTTGTTTTG
C13_181793271	AATGGTTCCTTTAGAGTCTTATCTACCATTACTTGTAGATGATACCAATTAGTTTAATCCTTTTTTATTAGTAATAAAATAAAATTTTACATTATAATAGAGTCCAAGGGTAGGTG<br>GACGCTTGGAGCATCTTCAATCTGCAGTAAAGAAATTAAAAAAAAAAAAAGAAGGAATATTATTCAAAATAGGTGTCAAATTAAA[T/A]TAAGTCTCCAATCTGCAGGTCATGTT<br>TGGTTTCATTAAGGTTGGATTGATACCCCTATACTTTATTTATATTTTATATAATAAGTGCTATATCTCTTCTTATATTATAACAAAATAACCATGACATGATCAACAATTATTAA<br>AACCAGCTGGGTGTAAAAGTTGACTGAACTTTTAATTTATGATGTAAAAGTTCATTAA
C13_181794691	GGGTGTAAAAGTTGACTGAACTTTTAATTTATGATGTAAAAGTTCATTAATTTCAGTTCTATACACGTTACCAAGCCTATTTCCTATGTTTGTTTGTTTTTTTTCCGGAAAAAGAA<br>ATACAAAATAAACTAAACATTTCTTTATCATGGTAATTGGTATGGCATATTTTTTTCATAATGAAATATATTAACAAAACATGT[A/G]AATTTGTATATACCCCTCTTCCATAATC<br>AACTGATTCTAAAAACACATATAAGTACGTAATACACTATGTTGTCAACTTTACGAAATAATTTGCTATTAGTAACATTAATTTGTCCTTTTATAACTTTTATATGTATTTATTAC<br>ACATATAATAGGAATAATAGATAACTCCACTAGATCTATGTACAAAAATAAAACAA
S13_225228475	AACTGCTTGAAACGAATGGTATACGCGAGATTATCGTCTCCAATTTGCTTGAGGGACCAAAACTCTTCCTCAAGTTTCTGTTGCTCGTGAGGAGGGCAAAATTCATCCATCATAA<br>GTTGCTTCAACTCTTCCCACGTCAATGCATATGCCAACTCGTTTGAACGAATATTCCTTTCATTCGTCCACCATTCAAGGGCTCT[T/C]GCTTGAAAGACACCAGTAGCACTACGT<br>GTCTTGAGTTCATCAGGGCATTCACTGTTGATGAAGGTAACCTCTATGCTGTCGAACCACTCGAACATAGCTGTCACACCTTCTTCACCAGTGAAGGGCTTAGGGCGGCAAGAG<br>ACGAAATGTTTGTAGTTGAAGGCAATGGGCTTAGGTTGGGCAGCTTTCGAGTCAATAGA
S13_225229830	TTGTAAGAGTATCTTCCAAACGTGTATTGTGTTAGCCTTAGGAAAGGCATGTATTTTAGAAGTCCAAAAGAATAGAAAGAAGCAAGTGAGATAGAATTGAAGTTGTTTTAAGCA<br>ACACATAGTGAAAAAGCTCCTAAACTATAGACTAGGCAAGGCATTCCTAATTCCCTATAGTTATGGCTCTGATACCAATCTGGTAT[T/C]AGAGTACTCCTACTATAGACTAGGG<br>AAAAGTATAGGAATTCTACCTAATTCCCTATAGTTATGGCTCTGATACCAATCTGTCACACCCCAACCGATGGCGGAAACATCGGGATGAGACGTACAAATTGCTCAAAACAAC<br>ATAACACTAAATGTGACAATATAAANNNNNNNNNNNNNNNNNNNNNNNNNNNNNNNNNN
S13_225246898	CAATCAAACAAAAAAGTCAAAAGTTAACTAGAGATATATTTAATTGTAGCAGATTTGATTATTAAAATTTTAACAAACTTCTAATAACTAGTTAAATCTGTCAAATTAGTCTACC<br>GGATCAAAAGTAAATATAAAATTTGTTAATACAAACAAAATAGGTAGATGATCCACATGAATCATAAACTAACATTTTCCGTAGT[G/A]GGGCGGGACATCATGCTGGAATCTG<br>TCATGGCGCCCTATAGCGTCCGGCCACACCACTATTGCCGGTGCTATGGGTTAAAATTTTTGCCCTCCCGCGATCTATATAACGGCGTTACCATTTCTTCCCCACTCACACACAT<br>ATACATACATAAATATATATATATAAAGCCCCATACATATATAACCTTACTACACACTCAAG
S13_225248384	TCATAAAGTCTACCAATGCATATTAAGACAACTTTTTTAAACTATACTTAATAATTCTTGATTAAGTAAGACCGCCTCTAGTGGAGATTTATGATATATGAAAGATGCTTGGGTG<br>GAAGAAAAGGGAGGACATGCGGGTGAAGACCACAAACGTGACAAGGAGAGGGAGAAGATGTANGGGAGAGGGAATGAGCCTCCGT[T/A]GGAATTCTGGTAGCATTTTCGTGT<br>ATTCAAAAATTGTTGTATGTCTTCTCAGTTTCACTAAAGTCATTTTGCATTAGAGCATATTAAAGGCATCTTATACTCTTCTTTCAAACTCCCCCACAAATATGTCATGTTAGAGT<br>TCTACTCATTCTTCAAACACATCATTTTTTTTCCAATGCTATCTCAATTTTTAAACTCGC
S13_225253042	AATTTTGGATTTGATGATTTTTGTATGAATTAGGTTATGATCAATTCATGAAATTTTATGAAATCGAGCACGAATATATGTTAGAATTATCATATGGACTGTGAATTTATCAAGAT<br>TTTGGTGTTAATTGAGGTTTGTAGATGAAACCCACTTGGGNTAANNNNNNNNNNNNNNNNNTCCTTGGGAGGGTAATTATTAAAC[G/A]AAAGCAATGATGTGGTCTTGGGGAAG<br>TTTTACAAATATGCACGAAACTGAAACCCACTTCTTGGACATAAGGCGATGATTACACGAAGGCACGAAACTAGTATGTGGATTGCGCACCTGGCCAGTACACAAGAAGCATA<br>TGACGTTCGTGAGACCGTGGTAATTATCGCAGGTATGTCTGGTAGAACTGGGTAGCAGGTG
S13_225265427	AAGAACCCCCGGCTGTTCTTTTCAGCATGTCGCCTTCCTCGAAGGTCTCTAATCCCTCATCCGGCAAACATGGCAACTCATCTAACACTTCGAGGTATTTCCGCTTCAACCTCCT<br>ATGATCCTCGTTATCGGGCTCCAGGTTCAAGCGATCGCTTAAAGTCTTCTCTATTTTGGACCTTAAAGCGGCTAACTCTTGTAAC[T/C]ATTCATCCACACTGCTCGCCTCTCCAC<br>CCGTACCCTCATCGTTTTTTTCAGCGGCAGACGAGTTCCCTATCTAGTCAACACCAAAAAGACAATTTAAGCTATATCGGTACACGTAAACAACAAAGTAACAATTTTTTACGTT<br>TTACCAGAAATGTTTAGCGTTTTAACAATTTACGTAAACTTTTCATTTTCACGTAAAA

S13_225270307	AGGAAATAAAACTGTAATCTCAAACCGCGGAATAGAATAGATGTCAACTCCTTCACATTTATCATTTGTACACACCAAAGATTTTGCATGATCAACATCAAATGAACCATCCAC<br>ATTTTCTTTCGTTACAAATCTTTCCTCCACACTCGACTTACAGTGGTTACAACCATTGTAATACCAAAGTTTATTAGTGCATATAG[G/C]TGTGATTGTGTCAAGAACCAAAACCT<br>TCTTTGTCTATCAATATCAAACAAAAATAATAATACTTAGAAAAGACTATAAAAAAACTCAGCATGTAAAGTACTTATAAGAAAATACACACCGTTGATATGGTTACAAGGAAA<br>CCAATAGGCATGAAATCAGGGTTATTAAGGAACTCATCTTCAACACTTGAGAAAACGTTT
S13_225273634	GATCAACGTGCGGCTTCAACAGCCAAATCATTATCATCTTTATCTCCTTCAGCAATATCCACTATGTTTTATTCGGTTCCTGGATCAAACATCTCGAACGTTTTCCCTTTTCATCA<br>AGACAATTAATCTGAAAAAAACACGTGGAACTAACAGCAAAAAGTACACCTGAAACAGAATCGTCGATTAAGAATAAAAAAACA[C/T]GTGGAATTAATAATGCATAGATTAG<br>TGAAGGAAACTTACGGTGCTGATTAAGAAAACTCCGTTTAATTGCTCAAATTGAGATGACGGGTGATCTGCAAGTGATTACAAATGAATCAATGCATCAATAAACGGTAAATCA<br>AAAGGTTACGAATACCAAAAAATAAATCAGACCAATTGAGGTTACCTTGGTGTTGTGCAAT
S13_225280292	CTGGCAATTTTACACGAATACACGAATACACGACACGAACCTACACGAAGTTAACAGGTATCGTGTATGGCCTTAACAGGTATCGTGTACCAAACAGGTAGACACGAGATTAC<br>CTGTTAATTTTCGTGTATTAACAGGTTAAATACCTGTTAATACCTGTTAGTACACGATAAGAAATTAAATTAAATAAAACTCATCAG[C/T]CATGTGCGTGTGGGTTCCTTAATTC<br>CTTTCTATTTCATTCCTCATTCAATTAAAAAAATAAATAAACCCCTAAACTCCCTTTGTAACTCTCAATTAGCATGTCGTGTTCGTGTTTTTGTTCGTGTTAACAGGTATTAACAG<br>GTAATTTTCGTGTATAATAGTCGAATAGTCGTGTTAACAGGTATTAACAGGTAATTTTC
S13_225287193	CGAGCACATAGTTTAGCATACCGAGCAAACCAAAGTGAGTTCACACTCTTACCATGGCATGGGATTCCCGGTGGGTTGGGAATGGGATTGAATGATTACTCGTACTTTCATCAC<br>TACTGGTTTACGTACCACTTGATGGGATTTTAACATGTTTATCAAAGCAATTTCATCCATATGAAATAAAAACGCAGCGGAAATAA[T/A]GTTTAAAACATGTTACTTTTATAAGA<br>TATAAAACCATTTGTATGCAAAATCCCAAAACCCGGATCCATATATGAAGATGCATACTATCAAACACAACCATAGGATGTTTAGAAAACTACCTTCTTGAAGTGCAAGGATTA<br>TGAAACGGATGATGCTTCTTGAGATGGTTGTCTTCAAGAGTAGAAGACCTCAAGGTTGTA
S13_225290789	GATAGAGGTCAAGCTGGGGGAGGCGACCTCACACGCTAAAACCGGCTAACCCTCACCGTCGAGCCAACCGGCGGTCAGAATAGGAAAGGGCGCTCAGAGAGAGAAAGAAAA<br>CATGATATTCAACTATATGTTAATAGCTTGGACAAAATGTTACTAATAGCAAATTATTTCGTAAAGTTGACAACATAGTGTATTACTTA[C/T]ATGTGTTTTTAGAGTCAGTTGAT<br>TATGGAAGAGGGGTATATACAAATTTACATGTTTTGTTAATATATTTCATTATGAAAAAAATATGCCATACCATGATAAAGAAATGTTTAGTTTATTTTGTATTTCTTTTTCCGGA<br>AAAAACATACAAACATCGGAAATAGGCTTGGTAACGTGTATACAACTGAAATTAATGAACT
S13_225294598	GAGCGTCAAGCGCCAAGCAATTTGAACAGCCCTAGTTGGTTCACTCAGAAAGAAGGACTTGATTAAGGTAGGATAAGATCTAGGAGACTGGAACCCCACATATATCATTAAAA<br>CCAAAACGGACCAAGAGTGTGACACTTTGAAATTTAATATTACATTTAATATCAAAGAGAGAAATCTTGGATAACTAAGAAGATATA[C/A]ACTAATAAAACATGGATGACAAA<br>GGATAAACAGTTAGCTTAAAAGTAAAAGAAGTAAAAGAGATCAGTGAGAAAAGTAGATCGGCATCCATATGTTTACGTTGTTGGTTTTCTTAACGGGGCGGGTGTGGGGTGAAG<br>GGTGGTGAATCCCTTCACCGCGTGGGGAACACCGCCGCCGTCCCCTATCACCGCACGACAAGC
S13_225297018	AAGGCCATACACGATACCTGTTAACTTCGTGTAGGTTCGTGTCGTGTATTCGTGTATTCGTGTAAAATTGCCAGCCCTAGTTTGAGCCACCTGATTTGAAAACTGCTCATTTCGA<br>GGAAATTTGCTTATATAATTCAGTGAAACACTCTAATATGTCTTTATTGATAGGTACACAAATAAAATGGGCCATGTGGGCCTAA[C/G]TTTCCACCTGATTTTCGCCAGCAATG<br>CAATACCATCAACAGGTAATGGTATATAATAAACTGAATATATGTATGTATAAGACTCCAAACAAACCATTCATGAACCAAAACAAATATGGCTAGTAAAGTTGCAAAGCTAAT<br>ATTCATCCCTTCCCCAGGGGTTGGTCACATTATGTCAACCGTTGAGATCGCAAAATTGCT
S13_225299005	CATTTGATTTGGGTGTTCGCTTAAACCGACGACAACCTTTTGATCCTTCTATTCCCGCGACATTTGACAAAACGATACCCCAACCACGAAGTTTCATCTCCAAGTTTTTGTCACA<br>CTCGATCATTCCACCTACACCTAGTCCATCATTCTCCACCTAATTTTAGCAGCCCAAAATCATATTGATGGTAAATGAAGAACCC[T/C]ACTTATTAGCCCAATTAGAAACCTGA<br>TTACCAATTACCCCGGCCCAAACTTACCTGACCAAACCTACTTAGGGTTGGTAGTTGGTTAGGACATCAAAATAAGACAATCCGAGGAACCCGATACCTGATTACAAAACCCG<br>AACGACACCCGTAATTGTATGTCATTAGGAATATGTAGGTAGGGAGGCCACAAATAATTGT
S13_225299812	TGCCCCCTTAAAAGTTTGGTTAATTTCTCAGATTGTCCTCTAGAAAATAAATCGTAGGTCCGGCCGAGAACCAAACCAATAATGCTTAATATGCCTGGTTCGGGTTGGGTATCCG<br>GGTTTTACTTGGTCCAACCCAAATCCTCTTCAGCACTATTGAATACATGAAATTAAAGTGGTAAATAGGGACCACTTCGGCCACT[C/G]TTTCGATCCACCACTTTGCCAAAATC<br>AACAAAGATATGATTAATTCCATTCCATTTAGCTCACTTATTAAAGTTGAGTAATTTTATAGGAATAAAAAAACTTTGAGCAACTTTAATAAAACAATGATAACTTTGTTAGTAG<br>GCTTGTAAACGAACCAAATGTTCAGTGAACCGTTTATATATCATTTGGCAGGAAACTCG

Map and sequence-based chromosome walking towards cloning of the male fertility restoration gene *Rf5* linked to R_{11} in sunflower

Guojia Ma[1], Yunming Long[1], Qijian Song[2], Zahirul I. Talukder[1], Md Shamimuzzaman[3], Lili Qi[3]*

Supplementary Table S4 Primer sequences of SNPs mapped in the present study

Primer name	Primer sequecns	Product length (bp)
NSA_001167F1	*GCAACAGGAACCAGCTATGAC*GACACTAAAAAAATGGAAGATAATATGCACA	137
NSA_001167F2	*GCAACAGGAACCAGCTATGACATGAC*GACACTAAAAAAATGGAAGATAATATGTCCC	
NSA_001167R	TCCAAAGGCCCAAAATAGGCT	
NSA_001379F1	*GCAACAGGAACCAGCTATGAC*GGTCACATGGCTGGCTGC	75
NSA_001379F2	*GCAACAGGAACCAGCTATGACATGAC*GGTCACATGGCTGGACGT	
NSA_001379R	GGTTCGACAAACAGGGCAAC	
NSA_004192F1	*GCAACAGGAACCAGCTATGAC*GGCTAGATATATTCTGACTGTGAATC	78
NSA_004192F2	*GCAACAGGAACCAGCTATGACATGAC*GGCTAGATATATTCTGACTGTGGCTT	
NSA_004192R	GTTTGGCTGCCGCTGAAAA	
SFW01515F1	*GCAACAGGAACCAGCTATGAC*CGTCCACTAGTCGTCG	49
SFW01515F2b	*GCAACAGGAACCAGCTATGACATGAC*CGTCCACTAGTCACCA	
SFW01515R	GCTCATTGTGTTTCAAAGTTAGTC	
SFW01741F1	*GCAACAGGAACCAGCTATGAC*AATGATGGGAATGCCATTT	53
SFW01741F2	*GCAACAGGAACCAGCTATGACATGAC*AATGATGGGAATGCCGCTG	
SFW01741R	CTGCCGTGTACAAGGTGTC	
SFW02101F1	*GCAACAGGAACCAGCTATGAC*AGCTAACTATTTCTTGAATAACA	57
SFW02101F2b	*GCAACAGGAACCAGCTATGACATGAC*AGCTAACTATTTCTTGAATGCCG	
SFW02101R	TGTCGATTATCGGTGAAGATCGT	
SFW03371F1	*GCAACAGGAACCAGCTATGAC*TCTTTTGGCTATTAGACAGG	47
SFW03371F2b	*GCAACAGGAACCAGCTATGACATGAC*TCTTTTGGCTATTAGATGGA	
SFW03371R	GTGGATCATGTGTTTACAATGCG	
SFW04100F1	*GCAACAGGAACCAGCTATGAC*GTTGAAAAGCTTGCAGTG	50
SFW04100F2b	*GCAACAGGAACCAGCTATGACATGAC*GTTGAAAAGCTTGCGATT	
SFW04100R	GGAAGTGATTGACCATAAGTGTCG	
SFW04482F1	*GCAACAGGAACCAGCTATGAC*TTCACAAGCCTCGGGCAT	40
SFW04482F2	*GCAACAGGAACCAGCTATGACATGAC*TTCACAAGCCTCGGATAC	
SFW04482R	AGAGGGGATTTAAGAATAAACCAT	
SFW04577F1	*GCAACAGGAACCAGCTATGAC*CACATTGCCCTAGACATCCC	66

SFW04577F2	*GCAACAGGAACCAGCTATGACATGAC*CACATTGCCCTAGACACTCA	
SFW04577R*	GCAACATACCGAAATTTGATCC	
SFW05176F1	*GCAACAGGAACCAGCTATGAC*GATGCTCTAGTATCGCCGTT	44
SFW05176F2	*GCAACAGGAACCAGCTATGACATGAC*GATGCTCTAGTATCGCTATC	
SFW05176R	CGCTGGGGTAATAGGGATGAA	
SFW07542F1	*GCAACAGGAACCAGCTATGAC*CCTTCGAACCGCCAGCT	49
SFW07542F2	*GCAACAGGAACCAGCTATGACATGAC*CCTTCGAACCGCCGACC	
SFW07542R	CGGGTTGATGGAAATGGTGG	
C13_175253519F1	*GCAACAGGAACCAGCTATGAC*CAAGTGAAGAACATGTGCACTGT	78
C13_175253519F2	*GCAACAGGAACCAGCTATGACATGAC*CAAGTGAAGAACATGTGCATCGG	
C13_175253519R	AAAGGGCCAAGGGTTACAGG	
C13_175253964F1	*GCAACAGGAACCAGCTATGAC*ACTGAACTAGTTATTGTTGGTTTAAGC	172
C13_175253964F2	*GCAACAGGAACCAGCTATGACATGAC*ACTGAACTAGTTATTGTTGGTTTGCGG	
C13_175253964R	TGTCGATGAAATGCGACTGCTA	
C13_175255719F1	*GCAACAGGAACCAGCTATGAC*GGGGATCGTCTTCTTCATTCC	136
C13_175255719F2	*GCAACAGGAACCAGCTATGACATGAC*GGGGATCGTCTTCTTCACCCT	
C13_175255719R	CAGATGGCAGTGGTCGTCTT	
C13_175260181F1	*GCAACAGGAACCAGCTATGAC*GATCTCTCCTAGTTTGCGCACA	76
C13_175260181F2	*GCAACAGGAACCAGCTATGACATGAC*GATCTCTCCTAGTTTGCGACCC	
C13_175260181R	CTCCGCTTCATCCACCCTAC	
C13_175261496F1	*GCAACAGGAACCAGCTATGAC*GCGAAGTCTGGAACAATATAACATCCTT	70
C13_175261496F2	*GCAACAGGAACCAGCTATGACATGAC*GCGAAGTCTGGAACAATATAACATTATC	
C13_175261496R	ACATGGAGCGTCCAAGCATT	
C13_175273046F1	*GCAACAGGAACCAGCTATGAC*CCACCACAACCCCACTAACCAA	80
C13_175273046F2	*GCAACAGGAACCAGCTATGACATGAC*CCACCACAACCCCACTAATAAG	
C13_175273046R	ATGGAGGCCCAAAAAGAGGG	
C13_175276537F1	*GCAACAGGAACCAGCTATGAC*CAATGGAAATCCTAGTTATATGAAAACCT	132
C13_175276537F2	*GCAACAGGAACCAGCTATGACATGAC*CAATGGAAATCCTAGTTATATGAAACTCC	
C13_175276537R	ACAGAACCAAAACCAGTTTCCC	
C13_ 181786257F1	*GCAACAGGAACCAGCTATGAC*GAATTTAAGTACTTAGTTAAAGGCCTAATTG	167
C13_ 181786257F2	*GCAACAGGAACCAGCTATGACATGAC*GAATTTAAGTACTTAGTTAAAGGCCTACCTC	
C13_ 181786257R	GATCCGCCACTATTCTCGGT	
C13_ 181787450F1	*GCAACAGGAACCAGCTATGAC*GAGTTTATGGTTCGGTGTACCCTTA	91

C13_ 181787450F2	*GCAACAGGAACCAGCTATGACATGAC*GAGTTTATGGTTCGGTGTACCACTG	
C13_ 181787450R	CAATCCCAACTCCACCACCA	
C13_181790141F1	*GCAACAGGAACCAGCTATGAC*GGCACACTTGAACCACTCAAT	107
C13_181790141F2	*GCAACAGGAACCAGCTATGACATGAC*GGCACACTTGAACCACTTGAG	
C13_181790141R	CCGACAAAACAAGGGCGATG	
C13_181792157F1	*GCAACAGGAACCAGCTATGAC*GGAGATAGCACGTGGTTTAGAACAA	94
C13_181792157F2	*GCAACAGGAACCAGCTATGACATGAC*GGAGATAGCACGTGGTTTAGAGTAG	
C13_181792157R	GCTTGGGATCCTCGTAGTCG	
C13_181792447F1	*GCAACAGGAACCAGCTATGAC*CTCTCCAAGTTTCTTTGCAAACCG	134
C13_181792447F2	*GCAACAGGAACCAGCTATGACATGAC*CTCTCCAAGTTTCTTTGCAAGTCT	
C13_181792447R	AGCTGAGCAACTCAGACACC	
C13_181792863F1	*GCAACAGGAACCAGCTATGAC*CCACCTACATAGAATCATTAACTGCAAA	91
C13_181792863F2	*GCAACAGGAACCAGCTATGACATGAC*CCACCTACATAGAATCATTAACTGACAC	
C13_181792863R	CCGCCATGAGTTGGTGAAGA	
S13_216344422F1	*GCAACAGGAACCAGCTATGAC*GCCTTCTTTGATATCCCAAAATTGG	180
S13_216344422F2	*GCAACAGGAACCAGCTATGACATGAC*GCCTTCTTTGATATCCCAAAACCGA	
S13_216344422R	AGGGTTTGGTGGAGCTCTTG	
S13_216392091F1	*GCAACAGGAACCAGCTATGAC*GCGTAACAGAGATTACCTTTTACACAT	184
S13_216392091F2	*GCAACAGGAACCAGCTATGACATGAC*GCGTAACAGAGATTACCTTTTACCAAC	
S13_216392091R	AACACGGGTCGTTTCTTAGG	
S13_225290789F1	*GCAACAGGAACCAGCTATGAC*CGTAAAGTTGACAACATAGTGTATTACCTAC	68
S13_225290789F2	*GCAACAGGAACCAGCTATGACATGAC*CGTAAAGTTGACAACATAGTGTATTACTCAT	
S13_225290789R	ACCCCTCTTCCATAATCAACTGAC	
S13_225294598F1	*GCAACAGGAACCAGCTATGAC*GAGAAATCTTGGATAACTAAGAAGATACAC	178
S13_225294598F2	*GCAACAGGAACCAGCTATGACATGAC*GAGAAATCTTGGATAACTAAGAAGATCTAA	
S13_225294598R	GATTCACCACCCTTCACCCC	
S13_225297018F1	*GCAACAGGAACCAGCTATGAC*GGCCATGTGGGCCCAAC	52
S13_225297018F2	*GCAACAGGAACCAGCTATGACATGAC*GGCCATGTGGGCCTCAG	
S13_225297018R	ATGGTATTGCATTGCTGGCG	
S13_225299005F1	*GCAACAGGAACCAGCTATGAC*AATCAGGTTTCTAATTGGGCTAATAAATG	157
S13_225299005F2	*GCAACAGGAACCAGCTATGACATGAC*AATCAGGTTTCTAATTGGGCTAATACGTA	
S13_225299005R	CGATACCCCAACCACGAAGT	

Nucleotides in red color indicate the SNP loci, and nucleotides in green color indicate the modified nucleotides.

The tail sequence is italized, and the additional five oligonucleotide insertion in AS-primer F2 is italized and underlined

Map and sequence-based chromosome walking towards cloning of the male fertility restoration gene *Rf5* linked to R_{11} in sunflower

Guojia Ma[1], Yunming Long[1], Qijian Song[2], Zahirul I. Talukder[1], Md Shamimuzzaman[3], Lili Qi[3*]

Supplementary Table S5 Sequences of the selected HA-R9 contigs and scaffolds

a, the selected contigs and scaffolds from the HA-R9 whole-genome sequence for *Rf5*

ID	Length (bp)
scaffold206293	3,988
scaffold550505	6,443
scaffold545194	3,878
C16577551	1,435
C16613275	1,480

b, the selected contigs and scaffolds from the HA-R9 whole-genome sequence for R_{11}

ID	Length (bp)
scaffold607601	1,724
scaffold585166	3,253
scaffold396498	4,018
scaffold359006	3,887
scaffold499821	1,257
scaffold588662	1,710
scaffold433233	3,394
C16653159	1533

c, Sequences of above mentioned contigs and scaffolds

>scaffold206293
TATTTGTTGATGTTACTGTTGTTTGTAGCCTATTAGGTGTTTAATTCCTTTGTA
CTTTTTCTTTCATAGGGTCTGTAATAGTCAATGGTTTTGCTGATGGAGTGTATG
TAATGTTGTCGAGGTGTCAGTGATGTTGTCTAAGTTATTGTTCTTTGTTGATCA
GAGGTTTTTGTTGATTTTTGTTTCAAAGAAAATAAAGTTTTTGATTTTTATTGA
TTTCTGTAGATCCGACCATATTTATCGCAGTTACTAATTTTTGTTGATTTTTTT
TTCATGTGAATCCCTAACTCCGTCATAACGAGCGGAATCCCACCATTCGTCAA
GCAACAGAGNNNNNNNNNNNNNNNNNNNNNNNNNNNNNNNNNNNNNNNNN
NNNNNNNNNNNNNNNNNNNNNNNNNNNNNNNNNNNNNNNNNNNNNNNNN
NNNNNNNNNNNNNNNNNNNNNNNNNNNNNNNNNNNNNNNNNNNNNNNNN
NNNNNNNTTATAATTTTATTTATGCTTTCTGTTCAGTTTTTTTAATTAATAATA
TATTCTGAATGTTTTTTGTGTTATTGTTTTTTTTAATTAATAATTTATTTAATTG
ATTTTAAATGATATTGTTTTTTTTTGTTGATATATGTAGTATGCCTTATGCTTT
ATCTTATTCTTATTAAATTAATAGTGATTTGGAAAATAGTATAATTTGATTTTT
CAGTAATCATTTTTTTAATTTTGTTTTAAAGATAACTTTAATTTCTTTTTAAAA

ACGTCTTTTTTCTATAGACAGATTAATATTTTTAAGCTCCGTTTCTACCGGTTT
GAAAACGTCTTTTTTTCTAGAAACTGGAAGAAACAGATCAATGCTTTAAAAA
CTTCTTTTTTTCTATAAACAAAATTAACTTTTTTATTTTTATTTATTTATGTACT
TCCGACCATATTTATTGCAGTTTACTTAATTTATGAAATTTAATTTCTTTTTAA
AAACTTTTTTTCTATAAACAGAGTAATGTTTTTATGCTTAATTTATCAATTTGA
TATTATAAGTATGTTTCTTTAATTAGACATTTTTTCTATAATTAGGTTGCCGTT
ATATTTTTAAAAATATAGTTACTNNNNNNNNNNNNNNNNNNNNNNNNNNNNNN
NNNNNNNNNNNNNNNNNNNNNNNNNNNNNNNNNNNNNNNNNNNNNNNNNNN
NNNNNNNNNNNNNNNNNNNNNNNNNNNNNNNNNNNNNNNNNNNNNNNNNNN
NNNNNNNNNNNNNNNNNNNNNNNNNNNNNNNNNNNACTTTTCTTGAATCGATT
TCTGAATTTCACATATCCCTCTACAAATCTGTAAGTTGTTTTTCTTAAATTCCT
TCTTTAATAATTATTTAAGTGTGTCTCCTTTTTTTTAATGTGTTTAAACTATTTT
TTTGTTTATTTAACCCTAAATCCCATTTGTATTTAAAAATATGTTCATTTTTGC
GTTTTAAGATTATGGTTTTGTTAACGAATACGTTGTTTCTGCAAAACCATCAA
TAAGAATATTGTACACTTGAATGTCAGAGTTTAGCTTGCTATCACCCACCAGA
TGAAACAAAGAGAGCACATCTTCAACTTGATGGTTGTTGCAAAGACCGGCTA
ATATTATTCTATAGGTGCATTCATCTGGAATTAGGCCTTGTGATCGCATCTCA
TCAAAGAGTTTGCGTGCCTCTCCACAACGCCCAACCCTAAACAGTCCCTGTA
ACATGGCACCGTAACTGACTATATCAGGTTTCAAACCTTTTCTTGNNNNNNNN
NNNNNNNNNNNNNNNNNNNNNNNNNNNNNNNNNNNNNNNNNNNNNNNNNNN
NNNNNNNNNNNNNNNNNNNNNNNNNNTCTGCAGGGCCTCCTCTATTCTAAAA
TTCTTGCAATACCCATTCAATAAACTGCTATAAGTTACAACATTAGGGACGAG
ACCTTTAAAAGTAAGTGAATCGAAAAGCGTCCTCGCTTTGATCATTTCACCTC
GTAGGCAGTAACCGTCTATAAGTGAACTGTATGTCACTATGTCAGGAACCTC
ACCTCTCTCAACCATGATGTCTATAACAGCCTCCGCTTCATTCACCTTACCAT
CCTTGCAAAATGCATCAACTAACATGTTAAAGGTTTGCAAATCAGGAGAGAT
CCTCACATCCTCCATTTCTTTCAGCATCTTATAGACCTCACCCCAACGACCTA
AGTTACAAAGGCCATGAATCAAAGAGGTGTATGTGATGACATTTGGTGGAAT
GCCTTTGTGAAATACCATTTCTTTGAAAAGGTTGAAAGCATCCTCTATCATTT
TGTCCTTGCAAAGACTATCAATGATGGTACTATATACAACAACATTAAGCTTG
CAGCCTCTTTCATCCATGAGCCTAAGCAAACCAATGGCAATATCATTGTTGCC
AAACTTGCAAAGCCCTTTGATCATTGTGGAATACATAACTACATCAGGTTCAC
ATAGTTTCTGTTTGATGAGCTTCTTGAAGAGTCTCTCAGCCTCAATAATTCTAT
CTTCTAGGATGAGTCCATCTAAGAGTGTACTAAATACGAACACATTTGGTAC
AACAGCACACCTGAAGCAAGAAGCTAGGAGTGCAAAACCGTCATTGGTGCG
AGACATCTGACAACAACACTTGATAGCAATACTGATAGAGTACGCGTTAACA
GGCACTCCAATGAGACACATTTGTTTGAAAAGGTGGAGACAAGAAGAAAAA
TGTTTCATTTTGGCAACATAATTTAACAACTGAGTAAACTTAACGACAGATGG
CAGTGGTCGTCTTTGAATCATTTCATCGAACAGGTTAAAGGCATGATCCAAGC
TGGTAACTTTTTGATACCTAGACAGGGGACGATCCCCAATATTTGAAAAGGA
AGTGGAGTGAAGAAGACGATCCCCATTATTTGAAAAGGAAGTGGAGTGAAG
AAGAGAAGCTAAAAGAGTGGAATGTAAACCTAATTGGGGAGATGAAAGAGG
GGTTGTTGTTAAAGAATTGGTGATGAAAATACCTTTGAGTTCGATGAAAGCA
CGACGAGTCATCATGATCCAACAGAGAAGAGAAGCAGAATCGATGAAGGGT
AAAATTGCAGCATCAATTTTAACCAACCACTCACTTTTGTTTGGTAAGAAATA
CAAATGATGCTGCTCCTAAGACCGCCGGTAATTGGGGCTTTTTTGTAAAAAA

AAATAATAAAATAAAAATAAAAATAAAAAAATTCACCAAACACACCGGAAA
ACGCCGGCTTCCGCCCCCTGGGCGTTATATTCGACAAAAGAAGGCCACCTGC
GTTTTTTAAAACACGCCGTTGCATGCTTTCTCAATTCTTGTTTGGCCAATAAG
AATATTCCAAATAATATTATTTAGTTTATTTTTATTTTTTAAAATAATTATGTA
ATCAATTGATTGGACATTATTGGGCATTATTCCACTACGCCCATTTGTGAAAA
ACGCCCATAATGCCCCTTTGCTTACTGGGATGACACGTGTCGCACAATGCCCA
TGGGTGGATCGAAATGTTTGATGAGCCCAATAATGTTGAATGTGGGATTTCAT
TAGTGATGTTACAAACACAATTGCCAATTCGTATTTGTTTTTTTATCTTTGGAA
TTCGATAAGAGTGTGAACTGTTAGGCCCAAACCCATTAATGAGATCCCAAAT
ATTATCTCTTTCCCTAGCCGTTNNNNNNNNNNNNNNNNNNNNNNNNNNNNNN
NNNNNNNNNNNNNNNNNNNNNNNNNNNNNACTTTTCCACATGAGATTCCATCC
ATCCCGCTAAATTTGACTTTTCCTTTATTTCATTGACTTTTCTTTAAGCTTGAC
TTTTATATCAGTTTGTTTATTTGAATTGTTTCATAAGGAGGGAGTCAAATAGA
AAGTATAGTTTGAGTAGTAAGAATATGTTAAGTATCGATTGGCTTGTTAATAA
TCAAGAAAATAGAAAGGTTCCAGACTAGAAATTTAGAAAGAAGTTGGATAAT
AATTTTCTGCATAAAAATGAAAATAACAAAGGGGTCTCCATATATGG

>scaffold550505
CCACTTCATTGACTATTCGAATTACAAACAAGCAAATCATAGGAAACATGCA
TGTAATTTCCAATAGTTGAGGACTATATGTGTTTTTTTATTAAACCACAGGGA
GCATTCGAGCATTTTACTCGATTTTTTATATTATTAACATCATTTCAACATCCT
TGTTTAGGGCAAGTCATCAATAATAACTTTTCTAACATTCATGCTCTTTATGTT
AGTTTATTCGAAAGAGGTAACCTTATCTAAAAAAACATATAGAAAAGCTTTA
CAACATTGGTAAGTTATCACTTATTGTGATACGTCCGTTTCAACTTTATGATCT
TATTGTAACTAAATCTCTTTAAATTACAAAAACATTGCAGGCAATAGACTAGC
ATTACATCCATTTGATAGTACTAAATATATCCCCGAGGTTCAAGACCTCATTT
ACTAATTTAATAGTACTACATATATTTTCTTTAGGTCTAATACCTCGTTTAATT
CTTCTCTCAAAATAGTGTGTATAATTTTCTCATGACTTAGGAATTATGATCTC
GTATTAATATCATTTATCCTCTAGGCTTATGGTCTCATTGAACACCATCTTCTT
GACCTCCAAAAATCAAATTTCATAACTATGATTATTTTTCATATTTGGGGACA
TTAGCCAAATAGTTGATTTCATAATTTTATTATTCAAACATATTCTTGAAATA
AAATCATTAACATGTATAAATTTCATCCCATATAAACACATAATAATAAGAG
ACTCTAATAATAAATAATATAGATTTCCAAATTCTCTGTTTTGAACGGTTTAT
AAGGATTTCAATATAAATTTGCTTATTTTCTTTAACGGCAAGGAACGACGTGC
CAATCACCATGACACCGGACGTTGTGGCCAAGGTCGACTACTCGACCGCCAC
CAGCGGCTTGGCTCTCTCTAGATGTGCGGAAACCTGACATCCACCTGCCCGA
AGGCATGATAGTGGAATAATCGGTAAAACCTCGCCTCCTATCCAAGTCGAAC
CGGCACCACCTGTAGTCGCTCTTCACCACTACCTCATTGGCATACAACAAGAT
TGTAATTCTCAATAAATAATTTTAATTACATTAAATAATATAGTTTGAAATCA
TAACCAAAAACAACATACCCGGTGATTTCTCACTCATAGCGAAAGTTGGGTC
TGGGAAGGATGGAATGTAGGCAAGTCTTACCTCTATCCTAGGGATAGAGAGA
CTATTTCCAATGAGACCCTCAACTCCAAAGTCAAAACCACACATAGAGGTCA
AAAACGATTGTACCTAGCTACTTAAAAATTGTACCTTTGCAAAATGTATAACA
TAAAGAAGTACCNNNNNNNNNNNNNNNNNNNNNNNNNNNNNNNNNNNNNNNN
NNNNNNNNNNNNNNNNNNNNNNNNNNNNNNNNNNNNNNNNNNNNNNNNNN
NNNNNNNNNNNNNNNNNNNNNNNNNNNNNNNNNNNNNNNNNNNNNNNNNN

NNNNNNNNNNNNNNNNNNNNNNNNNNNNNNNNNNNNNNNNATGCAAAAAAGT
ATTGGTGGCGTAGTGGTAAAAACCATCTCAAAATAATGCAAAGGTCTCCAGT
TCGAGTCTTTCCTCCATCACTAAGTTTTTATTTTTGTAATCATTTACCCTTAAC
CATAATTTTGGGCCCAATTCTTTTCGTCGCCTGAAGCCTACTTTTTTTCAAGAA
TTTTCGGGGACGGCTCTGGAAGTATCACATCAACAAGACACACAAACAAGCT
ATAGCATGCAAAGGCTACCCCCCACAACAAAATAAAATAAAATCCCTAGCAT
TGTAAGATAAGACTAAAAGAACAAACCGTACCTTATTCCTAAAAGCCTTTAA
CCTTAATTTTGCGTCTCTACGGACTCCTATCATTGACTATGTCCTCAGAAAGG
TGCAAATGCAACAAATCCTGCTTAATTTGCTCCTCCCAAGTCAGTTTCGTTCT
GCGTCTGCTCCTCCCCTCCACCGTAAGAGTCTCCACGGTTCTAACTGTGGTCG
TCACTTCTCGCCTCTAATTAGTAACAACCATAACTAAATGTTCTTTGTATGAA
CATACAACGTAAGATTGTGATTCATCATTTATTACACTAGATATCAAAATGGT
TATTTGAAGCACAAGTTCGACTAAGAAAAGTCAAATGTGTGAAATTGTTAGA
CAACTATGCCTTTGGAACGTTTTTAGATGTGTATTATATTATTTCGTAACAAA
AAGTGGATTAATAAAAGGTAAGTATTTGACGAGTCAATGAAAGAATAAAGTA
TGATTTTATATAAAAACCATTAAAGACAATATACACAGTTCGTGGGTGTTTGG
CTAAGCTTTTCAAAATCTCCTTATAACTTATTGACTTTTACAAAAAAGCTAAT
AAGGAGAAAAAAATGTTTGACAAACTCATAAGCACTTTTCAAAATAACTTTT
TGCATAGAGAGGAAAAGTTAGTTTTGAAAAGTTAGCAACTAGTGACTTTTTG
AAGCTCCTTATGACTTTTTGTCATTTTATATGAATAAGCTAAGACAAACACTT
TTTAAAAAACACCTTATTGGTTTTTCTCACCCCATAAGCTAATCCAAACACTT
TTTTAAAAACTCTTTTTCAAAAAGTTATAAGTCAATAAGTTCTTTTCACAAAA
AGTGATTTTTAAGTTAAATAAGCTTAGCCAAACAAGCCCTAGAGTCGAATGA
ATTGATAAAACAGGATAATATTGTTTTTCATCCATTTAAGTTGAACGGGATGG
TTAGATTGGGTATCGGGTGAGGCATCTTTGGGTAGAAAATTAATCCAGTAATT
TTCAGTTCGGATATTGGTTAGGGAAATTCAACATCGGTAACGCGACTTACCCG
AGTAAGAACTAAGCGTGATCCGGCTTTCGTCAGGCGGTGATTTGTTGTGGAA
GCGTCTTTTCATCTAACCCTCTATTTCCCCTTTTTATTTTATCATCATCATTCCC
CGCCCGATCAACACCCACAATCGAGTATTAACAAATATGTCCAAGCCCGTAC
TACCTACATAAACCGACCCAATNNNNNNNNNNNNNNNNNNNNNNNNNNNNNN
NNNNNNNNNNNNNNNNNNNNNNNNNNNNNNNNNNNNNNNNNCCAAATAACC
AAACAACCTGAACTCGACCACCCGATTGTGATACGAATTGAATGTCGGATGC
TATATCATATACAAAAACCCGAATAACCCAACCCAAATCCATGGTTTTTATTC
TATCATTAGGCGTTAGGTTTATAGAAACACTAACATCTCGCAATGCACTGGTG
ACAGTAAACAATCATACAAGCATATATAGAGCCTTAGGGGCATTGAGATACA
TCAGCTCAAATATTATTTTATTATTGAATTTTAAACCGATCTCTAATCCAGATC
TTAAATTCCCAACAAGATCCGTCTTTGCCACTGACTGATCGCTTCATGATCCT
TGATCTCTTGTACCTGCTAATAAGAATCATATATTCTTTGCTTTTTATGCCCTC
TCGGCAACCTAGTTTCTTGCTTCTCGAGCCCATCGGTTCGCATCACCATTATG
ATAATCACAATCATGATTTACTTCCTTCCTTTTTTCCCNNNNNNNNNNNNNNN
NNNNNNNNNNNNNNNNNNNNNNNNNNNNNNNNNNNNNNNNNNNNNNNNNNN
NNNNNNNNNNNNNNNNNNNNNNNNNNNNNNCCGAACTCAGCAACTAACAAGC
ATTTGCAGATTCATAATGCGTATGGGTACATATGTAGAAATAGAAAGTAATG
TGATTACCCACTTTGTAGATATCAAACCGGAATTCATATATAAAGTCGATGAA
ATGTGACTGCTAGTATTAAAAATACAAATATGTTGAACGATAAACCCATTAG
ATAGAATATACCACAAGTAAAGACCGGAAGTAATTCGATACAAATTTCCGAT

TTTAATTTCTCATCCTACAAAAGTACAAATAGCTCAGACCTACAATAACTAAT
GCAGTCGCTCTCCCATATTGCTATCTAGGCAGGAGTGTCCATCATTTCTTTTG
GGACAAGCTTACCTATCAATTTAAGCATACTTCTATCTACTGAACCAGCTGCC
ATTTCATCAATCAACATCGATAAAGTTGAAGCATCAACTGAGTACCTTCTTCC
ATCCATTTCCTGTAAAAGCATCTCAACATCATCGTAATTCTGGTTCTTAAAAT
ATCCCTGGAGAAGAACATTGTAAGTAACACCATCTGGTGGGCAGCCACTCTC
ACCCATTTTACGAAACAAGTGATTTGCTTCCTTTAGTAAACCTTCCCCACACA
AGCCACTAACCATCACAGTATATGTCCGAACATCAGGTTGCAAACCTTTTAG
AGTTAGGTCTTGGAAAAGATCCCTTGCAATATCAAGCTTCCCACATTTGCTCA
TACCATCAATAAGAATATTGTACACCACGATATTATAATTTAGCTTGCTATCA
CCAACCAGATGAAACAAAGAGAGCGCCTCTTCAACTTGATGGTTGTTGCAAA
AGCCATCTAATATTATTCCGTAAGTGCATTCATCTGGAATAAGGCTTTGTGCT
CGCATCTCATCAAAGAATTTGCGTGCAGCTCCACAACGTCCGACCCGAAACA
AACCCTGTAACATGGTGCTGTAAGTGACGATACAAGGTTTCAAACCTTTTCTT
GTCATCTCATGAAACAAATGCATGGCCTCTTCTATTTCAAACTCTTGCAATA
CCCATTCAGTAAACTGCTATACGTAACAACATTAGGGACGAAACCTTTAGAA
GTAAGTGAATCAAAAAGTGTCCTTGCTCTGGTCATTTCACCTCGCAGACAGTA
ACCGTCTATAAGTGTATTGTATGTCACTATGTCAGGAACCACACGTCTCTCAA
CCATGATGTCTATTACAGCCTCCGCTTCATCCACCCTACCGTCCTTGCAAAGT
GCATCAACTAATATGCTAAAGGTATGCAAATCAGGAGAGATCCTTAGATCCT
CCTCCATTTCTTTTAGCAGCTTAGAGACCTCGTCCCAACGACATAAGTTACAA
AGACCATGAATCAAAGAGTTGTATGTGATGACATTTGGTTGAATGCCTTTGTG
AAATACCATTTCATTGAAAAGCTTGAAAGCATCCTCTATCAATTTGTCCTTGC
AGAGACTATCAATGATTGTGCTATATACAACAACATTAGGCTTACAGCCTCTT
TCATCCATGAGCCTAAGCAAACCAATGGCTATATCATTATTACCAAACTTGCA
AAGCCCTTTGATCATAGTGGAATACATAACTACATCAGGTTCACATAGTTTCT
GTTTGATGAGCTTCTTAAAAAAATTCTCTGCCTCAAGAATCCTATCTTCTATG
ACGAGTCCATCTAAGAGTGTACTAAATATGTAGACATTTGGGACAACAGCTC
GCCTGAAGCAAGAAGCTAGGAGGGCGAAACCGTCATTGGTGCGAGACATCT
GACAACAACACTTGATTGCAATACTGATAGAGTACTCGTTAACAGGCACTCC
AATGAGACACATTTGTTTAAAAAGGTCAAGAGAGCAAGAAAAATGGTTCATT
TTGGCAGCAGCATTCAACAGTTGATTAAACTTGACAACAGATGGCAGTGGTC
GTCTCTGTGTCATTTCATCGAACAGGTTAAAGGCATCATCCAAGTTGGCAACT
TTTTGATACCTAGACAGAGGACGATCCCCATTATTTGAAAAGGAATTGGAGT
GGAGAAGAGAAGCTAAAAGGGCGGCGTGAGGACTGAATCGGGAAGAAGAA
AGAGGGTTTTGTAAAGGAAGAAGAAAGGAGTCACCTTTGAATCTGATGAAAG
CACGAAGATGAGAAATTATCATCGTCTGAGTCTCCAACAACAGAGAAAGCAG
AATCGATGTTTATTCCTTCAACCTAACAGTAACACACATTTTGGTTGGACACT
TGGATGAAGATCGCTTTATTGATGCCTTAAAAGCCCTCAAGAAGATTGCTGTA
TTGATGCCCTTAGGGGAGGGGGTGGTTCACTAGTGATAGAATTTATCACTCAT
AAGCACCAATCAAGTTTCGCCATGTCATCGACCATTTTTCCATCACTCACAAC
CTTTTTTAGTGGGGGTGGTCATCACTCATCACCACACCCAACAATTTCCCCCA
ACCAACAAATACCCTCACAAAACAAAACATTAACGCGTGATGGAGATAACG
AAAACCGGATTGCGTTAATGTATCACGCGTTTTAGTTTGTACCGGCGGTCGTC
GAAAACGCGTTATAAGCATTCCCGTTATACTATCACGGGACCACCCCGTGAG
CTCTTAGAATAAAGAATTAAATACTAATTCTTTCTCTTCTACCTACACAACCC

AACCTAATCCAGTTTTTCACCCACATTCATAACCCAACCTAATCCAGTTTTTC
ACCCACATTCATAACCCAACCTAAGACCGTGGGGTATGGTGGGGCGGGGGTT
TGGGGCATGGGTTGACACGTGGCTTGGGGGGGGGGGGGG

>scaffold545194
ATAATCAAATTCAAAATAAGCAGCTTCTTGTCAAATGGCATTTAGGGCATGCT
CAGTTGCTCACCAAACCAAAATCAGAGCAGCTTCTTGGTTTAAACATCAATTG
ACGTCTCTCCGTAACGGAATCAGCGACGATCGATGATGACTCACTTGAATTC
ACATCTTCATGCTTTCATCAAACTCACAGGTAATTTCATCACAAACCCCTTCC
TTCCTCCTTTGAAATACCCTCTCTCATCTTCCCGATTAGGTCTCGAATTCAGTC
CTCACTCCACTTCTTTAGCTTCTTTTCTTCACTCCACATTCTTTTCAAACAATG
GGGATCGTCCACTGTTATCTAGGTATCAAAAAGTTACCAGCTTGGATCATGCC
TTTAACCTGTTCGATGAAATGATCCAGAGACGACCCCTCCCATCTGTTATCAA
GTTTAATCAGCTGTTGAGTGCTCTTACCAAATTCAACCATTTTTCTTCTTGCCT
CCATCTGTTTAAACAAATGTGCGTCGTTGGAGTTCCTGTTGATGCCTACTCTA
TGAATATTGCCATCAAGTGTTGTTGTCAGATGTCTCGCACCAAAGACGGCTTT
GCACTCCTGGGCTGTTGCTTCAGGCGAGCTGTTGTACCCAATGTCTTCACATT
TAGTACACTCTTAGATGGACTCATTCGTGAAGATAGGATTCTTGAGGCTGAG
AGACTCTTCAAGAAGCTCATCAAGCACAGACTTTGTGAACCTGACGTTGTTAT
GTATAACACGATGATTAAAGGACTTTGCAAGTTTGGCAACAATGATATTGCC
ATTGGTTTGCTTAGGCTCATGGATGAAAGAGGCTGTAAGCCTGATATTTATGC
ATATAGTACTATCATTGATAGTCTCTGCAAAGACAAAATGATAGAGGATGCT
TTCAAGCTTTTCAATGAAATGGTATTTCACAAAGGCATTCAACCAGATGTCAT
CACATACACCTCTCTCATTCATGGCCTTTGTAACTTATGTCGTTGGAACGAGG
TCTCTAAGCTGCTAAAAGAAATGGAGGATGTAAGGATTTCTCCTGATATTCA
AACCTTTAACATATTAGTTGATGCATTTTGCAAGGAAGGTAGGGTGGATGAA
GCGGAGGCTGTTATAGACATCATGGTTGAGAGAGGTGTGGTTCCTAACATAG
TCACATACAATTCACTTATAGACGGTTACTGTCTACGAGGTGAAATGACCAA
AGCAAGGTCCATTTTTGATTCACTTACTTCTAAAGGTGTCGTCCCTGATGTGT
TTTCTTATAGCAGTTTGCTGAATGGGTATTGCAAGAGTTTGAAAATAGAAGA
GGCCATGCATTTGTTTCATGAGATAACAAGAAAAGGTTTGAAACCTAATATA
GTCAGTTACAGCACCATGTTACAGGGTTTGTTTAGGGTCAGACGTTGTGGAG
ATGCACGAAAGCTCTTTGATGCGATGCGAGTAGCAGGCCAGATTCCAAATGA
ATACACATATGGAGTAATTTTAGATGGCCTTTGCAACAACCATCAAGTAGAA
GAGGCGCTCTCTTTGTTTCATTTGGTGGGTGAGAGCAAGCTAAATTATAATAT
CGTGGTGTACAATATTCTTATTGAAGGTGCAAGCAAATGTGGGAAGCTTGAT
ACTGCGAAGACTCTTTTCCATGACCTAACAAAAAAAGGTTTGCAGCCTGACG
TGCGAACATTTAACATAATGATTAGTGGCCTCTGTAGAGAAGGTCAGCTAAA
GGAAGCAAAGCTCTTGTTTCGTAAAATGGATGAGAGTGGCTGCCCACCAGAT
AGTGTTACCTACAATGTTCTTCTCCAAGGATATCTTAGGAACAAGCACTTTGA
TGATGTAGAGATGCTTTTACACGAAATGGATGGAAGGAGTTACTCACTTGAT
GCTTCGAGTTTATCTTTGTTGATAGATCAAATCGCAGCTGGTTCACTAGATCG
ACGTATGATTGAGTTGATAGGTAGGCTTGTGCCAAAAGAAATGTTGAACATT
CCTGCCTAGATCTGTTGGTAAGTTGTTCACCTATCACAATTTCCGACTTGTCAT
GCTATTTTATCTGCTTCATCTATCAGATCTGTTGACTATCTCTTCTTAGGAGAT
TCTGTTGATCCNNNNNNNNNNNNNNNNNNNNNNNNNNNNNNNNNNNNNNNNNN

NNNNNNNNNNNNNNNNNNNNNNNNNNNNNNNNNNNNNNNNNNNNNNNNNN
NNNNNNNNNNNNNNNNNNNNNNNNNNNNNNNNNNNNNNNNNNNNNNNAGA
AAGTTGCTTTTAGTTTGGTAAGAATCATGATTCACCATGTATCTTGCGAGTGT
TTGACTTTTCAAGGTTGACTATCCACAAAACAACTGTTTGACTTTCAAGTAAA
TCTAAATCTGAGGTTGTTCTTTTTGGGTACAGGGTAAAATGGGAATTTGACAT
ATTATTTATCTTGCTTATGCAAATCTAAATCTGAGGTTGTTCATTTTGGGTACA
GGGTAAAATGGGAATTTGACATGGGTCAAAACAGGGTCGAGTTGGTTCAAAA
ACTTTTTGTTCAATATTATCATTTTTTTGCTTTTTAAAATATTTGTACGTCTGTT
ATTCTTTTAAAGAGTTCAAAACCTTGACATATATGTATTTCATTGATACTAGA
AGTAACATTTCATTGACTTTATATGTTGCTTCCAGTTTATTAGGGGATATCGT
AGCTTTGATCGCAAGCGTCTTTTAGTTTAATCNNNNNNNNNNNNNNNNNNNN
NNNNNNNNNNNNNNNNNNNNNNNNNNNNNNNNNNNNNNNNNNNNNNNNNN
NNNAAACATTTTTGGGCTACTAGTGTAGGCTGCAGCATTGCTTTTGTTGATCA
GCCCTGAAGTCGCGCCCGGTGAATCTGTCCTCAGAAACAGAATCACCAGATT
TGCAGTGTTTTAGTAATTTTTAGTAGAAATAGTAAGAGCGGGATCAGATGGC
AAATGACCGTCTGGAGTTTTGTCCACAACCTGCCTTAGTTTGCCATTATCTAA
CTTGAGATTTTGTTAGGTTTAGAAGCAAGTCTCAGGTTGGCTACGTAAGATAA
TTTTATAACTCACAGATTTAGATAACATTGAATGGTTTTCTTGTTTGTATAAAT
TAATAACTAGTTTTTGTCCCGCCCGCGTTGCGGTGCACTAAGCCGAATACTTC
TCTCTCATAACATCTACGTTTGTGTTTCGGTTACTTGTACATACTACTCATAAC
ACGATCTAAAAAAATACATTTAGTCAACCAATTAAAATAAAACACCACCATT
CTTTTGCTAAAAAGACAAAAACGATGGAAAAACTATAATTTTAAACTGGGGG
CAAAATTGTAACTTTTCAAGACAAATGAGCGTGTGTCAGGCAGCCTGACACG
AATAAAAATTACACCGAGTCAACTAATTAAAACAAAACAGTACCATATTTTT
GTAAAAAAAATAATGGCATGACTGTAATTTTACACTGGGGTAAAATCGTAAT
TTGACTGGGAGAACAAACAAAAACGATTGTAAAACTGTAAATTTGAAATGAG
TGCAAAATTGTAATTTGGCTGGAAGGGAAAAAACAAAACTAATGACAAAACT
GTAAATTTAAGCGAGGTAAAATCGAAATTTGGCTGGACCAATAGTGAAGTGT
CGCTGCCTGACACTATACCCTTTCATGTAAAATGTATAGTACTAGTTAAGTTA
TTGCCCCGCCCACATTGTGGGGTACTAAGCCGAATATTTCTATCTCATAACAT
GTATGTCAGTGTTTCGGTTACTTGTACCTACTACTCATAACACGTTCTCAAAA
AAAAAATACATCGAGTCAATCAATTAAAAACACTGCGATACTACAAAAGAA
AAAAAAAACTAAAACGATGGAAAGACTAAAATTTTAAATTGTGGCAA

>C16577551
ATGGGGATCGTCCACCATCTAGAAATCAAAAACTTACCAAATTGGATGACGC
CTTTAACCTGTTTGATGAAATGCTACACACACGACCACTGCCATCTGTCGTTA
AGTTTACTCAGTTGTTAAATTCTGTTGCCAAAATGAAACATTTTTCTTCTTGTC
TCCACCTTTTCAAACAAATGTGTGCCGTTGGAGTGCCTGTTAACGAGTACTCT
ATGAGCATTGCAATCAAGTGTTGTTGTCAGATGTCTCGCACCAATGACGGTTT
TGCACTCCTAGCTTCTTGCTTCAGGCGTGCTGTTGTACCAAATGTGTTCGTATT
TAGTACACTCTTAGATGGACTCGTCGTAGAAAATAGGATTGCTGAGGCAGAG
AGACTGTTCAAGAAGCTCATCAAACAGAAACTATGTGAACCTGATGTAGTTA
TGTATTCCACTATGATCAAAGGGCTTTGCAAGTTTGGCAATAATGATATTGCC
ATTGGTTTGCTTAGGCTCATGGATGAAAGAGGCTGTAAGCCTAATGTTGTTAT
ATATAGCACCATCATTGATCGTCTCTGCAAGGACAAACTGATAGATGATGCTT

TCAAGCTTTTCAAAGAAATGGTATTTCACAAAGGCATTCCACCAAATGTCATC
ACATACACCTCTTTGATTCATGGCCTTTGTAACTTAGGTCGTTGGGGTGAGGT
CTATAAGCTGCTGAAAGAAATGGAGGATGTGAGGATCTCTCCTGATATGCAA
ACTTTTAACATATTAGTTGATGCATTTTGCAAGGAAGGTAGGGTGGATGAAG
CGGAGGCTGTTATAGACATCATGGTTGAGAGAGGTGAGGTTCCTGACATAGT
CACATACAGTTCACTTATAGATGGTTACTGCCTACGAGGTGAAATGATCAAA
GCGAGGACGCTTTTCGATTCACTTACTTCTAAAGGTCTAGTCCCTAATGTTGT
TACTTATAGCAGTTTATTGAACGGGTATTGCAAGAGTTTGAAAATAGAAGAA
GCCATGCATTTGTTTCATGAGATAACAAGAAAAGGTTTGAAACCTGATATAG
TCAGTTACGGTGCCATGTTACAGGGATTGTTTCGGGTCGGACATTGTGGAGCT
GCACGCAAACTCTTTGATGAGATGCGATCACAAGGCCTAATTCCAGATGAAT
ACACCTCTAGAATAATATTAGACGGTCTTTGCAACAACCATCAAGTTGAAGA
TGTGCTCTCTTTGTTTCATCTGGTGGGTGATAGCAAGCTAAACTCTGACATTC
AAGTGTACAATATTCTTATTGATGGTATGAGCAAATGTGGGAAGTTTGATATT
GCAAGGGATCTTTTCCAAGACCTAACTCTAAAAGGTTTGCAGCCTAATGTTCG
GACATATACCGTGATGGTTAGTGGCTTGTGTGGGGAAGGTTTACTAAAGGAA
GCAAAGCACTTGTT

>C16613275
CATCGGTTCGCATCACCATTATGATAATCACAATCATGATTTACTTCCTTCCTT
TTTTCCCTAGCTCAGGTCTGATCGAACTCAGCAACTAACAACCATTTGCAGAT
TCATAATGCTTATGCGTATGAGTACATATGTAGAAATAGAAATGAATCTGATT
ACCCACAACCGTTGGGCAGGAAGAATGTGCAAAAACTGAACAAGTATGGCG
ATTCTGGGGCACATCCACTGCAGAATTCAGTAATGTTCTAATACAGGACTGAT
TTTTATAAAACATTGCTTGTGATCAACGCTGTGATATCCACTTTGTAGATATC
AAACCGGAATTCATATATAAAGTCGATGAAATGTGACTGCTACTATAAAAAA
AATACAAATATGTTGAACAATAAACTCATTAGATATAATATACCACAAGTAA
ACACCAGAAGTAATTCGATACAAATTTCCGATTTTAATTTCTCATCCTACAAA
AGTACAAATAGCTCAAACCAACAATAACTAGTTCAGTCTCTCTCCCTGATTGT
TATCTAGGCACTGCAGTAGTATCCATCATTTCTTTTGGGACAAGCTTACCTAT
CAACTTAAGCATACTTCTATCTACTGAACCAGCTGCGATTTCATCAATCAACA
TCGATAAAGTTGAAACATCAAGTGAGTACCTTCTTCCATCCATTTCCTGTAAA
AGCATCTCAACATCATTGTAATTCTGGTTCTTAAAATATCCCTGGAGAAGAAC
ATTGTAAGTAACACCATCTGGTGGGCAGCCACTCTCACCCATTTTACGAAACA
AGTGCTTTGCTTCCTCTGGCATACCTTCTCTACATAGGCCACTAATCATCACA
TTATATGTCCGAACATTAGGTTTCAAACCTTTCAGAGTTAGGTCTTGGAAAAG
ATCCCTTGCAATATCAAGCTTCCCACATTTGCTCATACCATCAATAAGAATAG
TGTACACTTGAATGTCAGAGTTCAGCTTGCTATCACCCACCAAATGAAACAA
AGAGAGCACATCTTCAACTTGATGGTTTTTGCAAAGACCGTCTAATATTATTC
TATAGGTGCATTCATTTGGAATTAGGCCTTGTGATCGCATCTCATCAAAGAGT
TTGCGTGCCTCTCCACAACGCCCAACCCTAAACAATCCCTGTAACATGGTGCT
GTAACTGACTATATCAGGTTTCAAACCTTTTCTTGTTATTTCATGGAACATAT
GCATGGCCTCTTCTATTTTCAAACTCTTGCAATACCCGTTCAATAAACTGTTAT
AAGAAAACACATCAGGGATGAGACCTTTAGAAGTAAGTGAATCAAAAAGCA
TCCTTGCTTTGATCATTTCACCTCGTAGGCAGTAACCGTCTATAAGTGAACTG
TATGTCACTATGTCAGGAGCCTCACCTCTCTCATCCATGATGTCTATAACAGC

CTCCGCTTCATCCACCCTACCTTCCTTGCAAAATGCATCAACTAATATGCTAA
A

>scaffold607601
GGTTATGGGGAGAGAGACGAAAATTGATGTTATGAGGGTTTGTGATTGTGTA
ATCTGTGTAACCCTTAAACTCTCTAATAATCCCCTTATTTATACACCCAAGAG
AAACCTAATTAGTTAATAAGGGTAATATGGTCCATTAACAATTACCAACTAA
TTATTAATAGGTTATTAATATATTTTGATCTAATATAATATAAATGATTATAA
AGGCTACAAGATTAAATATTACATTAATAATATATTTAATCTCACAATTTTGT
TATAATATAAGAAGAGATATAGCACTTATTATATAAAATATAAAGTATAGGG
GTATCAATCCAACCTTAATGAAACCAAACATGACCTGCAGATTGGAGACTTA
TTTTAATTTGACACCTATTTTGAATAATATTCCTTTTTTTTAAATTTCTTTGCTG
CAGATTGAAGATGCTCCAAGTGTCCACCTACCCTTGGACTCTATTATAATGTA
AAATTTTATTAATAATAAAAAAAAGATTAAACTAATTGGTATCATCTACAAA
TAACGGTAGATAAGACTCTAAAGGAACCATTCATGAACCAAAACTAATATGG
CTAATACATTTGCAGAGCTAATATTCATCCCAACCCCTGGGCTTGGTCATATC
ATGTCAACCATAGAGCTCGCAAAGTTACTTGTGAATCGCGATCAACGTCTCG
CGATAACCGTCCTTGTCATCAAGCCTCCTGGCATGACTTCTGGCTCGGCTATC
ACCACCTACATAGAATCATTAACTGAAAACACCATGGATCGCATATCCTTCAT
TCGACTCCCTCAAGTTGAATCTCCACCNNNNNNNNNNNNNNNNNNNNNNNN
NNNNNNNNNNNNNNNNNNNNNNNNNNNNNNNNCCAACGGAATTCATCAGAA
GTCACTCCAAATATGTTAGAAATGCAGTAGTTGATTTGAGAAGTCAATCGGG
TTCACGTCAGGTTGTTGGGTTTGTCGTCGACATGTTTTGCACCAGCATGATTG
ATGTGGCTAATGAGTTTAATGTTCCAACCTTCGTTTTCTTCACTTCTAGTGCTG
CGTTTCTTGGATTTACGTTATATATAAAATCACTCTGTGATGATTTGAATAGA
GACGTTGTTGAGCTGAGCAACTCAGACACCGAGATATCGGTTCCAAGTTTTGT
CAAACCGGTGCCAACTAAAGTGTTTTGGAGTCTTGTCAAGACTAGAGAAGGA
CTGGACTCTGTTCAGAGGCTTGCAAAGAAACTTGGAGAGGCCAAAGGAATCA
TTGTAAATACATTCTTGGATCTAGAAACACACGCAATAGACTCGTTGTCTGCT
GACAGTAGTATCCCGCCGGTATATCCGGTGGGACCCATACTCAACTTAGAAG
GCGGTTCTGGTGGTGGGAAACCTTTTGACGATGACGTCATTAGGTGGTTGGAT
AGTCAACCACCTTCCTCGGTTGTGTTTCTGTGTTTTGGGAGTATGGGAAGCTT
TGATGAGGCCCAAGTGAAGGAGATAGCACGTGGTTTAGAGCAAAGTGGTCAC
CGTTTCTTGTGGTCCCTACGTCGACCTCCATCAGAACAAACGACTACGAGGAT
CCCAAGCGACTATGAGGATCCGAGTGTCGTACTGCCAGAAGGGTTTTTGGAT
CGAACACGTGGAATAGGGAAAGTAATCGGGTGGGCCCCACAGGTGGCGGTG
CTGGCTCATGATGCAGTTGGAGGGTTTGTGTCCCACTGCGGGTGG

>scaffold585166
AAAACCAAAACCCAAAAGCCAAAACCCCAAAACCAAAAACCAGAAACCCAA
AATCGCAAATCAAAACCAATGCCAAAACCCAAATGGCCAAGTTAAATTTTGA
AAATCCCATAATTCAACTTGAGGGGGAGTGTTAAAGAAAAGGCAAAGAAAT
AATTCAAATATTATGTTTCCTATTCTGTCGTGATTATGTTTCCTTTTATGTGTC
TTTGGTTTTGTATTTAATCTTTGCTAGAATTATCAATAAAATTAAGTTCTTTGA
TTATCATTCACAACAGTGTGAGAGTTTATTATTGGGTGTTGTGAATGATGACC
ATTGCCATTAAAAAAGGTTGTGAGTGATGGAAAAATGGTTGATGACATGGCG

GAACTTGATTGGAGGGTGTTGTGAGTGATGAGTGAGTGATGGGTGATGACCA
CCGCCACCCCCCTTACATTGTTTATTTAGCTATCTCGTAACTTTAATACGTCTA
TATAAACAAATTTCAAATTCGTAATAAAAGAAAATGTTCTCAGATACAGTGG
CGGGGCTTGAACAAATATATAGGGGGCCAAACTCAAGAAAATCAAGGGGCA
AGGAATTTTAGGCAAAATCAATTTGGGGCCGGACTCAATACGTATATACAAT
TTTCTGACGAAATACGCGTAAATTTACACTGCTTTGCAAGGGGGGGGGGTCG
AGCACCCCCGGGCCTCTTANNNNNNNNNNNNNNNNNNNNNNNNNNNNNNNN
NNNNNNNNNNNNNNNNNNNNNNNNNNNNNNNNNNNNNNNNNNNNNNNNN
NNNNNNNNNNNNNNNNNNNNNNNNNNNNNNNNNNNNNNNNNNNNNATATG
AATATCATATGACCTTGCTTTTTTAAAGATTTGAACCCACACTATTTTTGCGG
GACAGATACCATACTAGCCACCATAGCATTAAGGTCATGGGTAGCATGGAGA
CCCACACGTTTAAAAATAATGGAATTTTTATCACCCCTAGAAATTTGGTTCTT
GATTGATCTCTCGTTGATTAATTTTTATAAATAAGTATTCTTTCAAAACATAA
ATTTGAAATAAATAACCTTTAGCAAAGTTATTGTTTTATTGAAATTTGAAATA
AATAAGTTTTAACAAAGTTATCATTGTTTTATTAAAGTTGCTCAAAGTTTTTTT
ATTCCTATAAAATTACTCAACTTTAATAAGTGAGCTAAATGGAATGGAATTA
ATCATATCTTTGTTGATTTTGGCAAAGTGGTGGATCGAAACAGTGGCCGAAGT
GGTCCCTATTTACCACTTTAATTTCATGTATTCAATAGTGCTGAAGAGGATTT
GGGTTGGACCAAGTAAAACCCGGATACCCAACCCGAACCAGGCATATTAAGC
ATTATTGGTTTGGTTCTCGGCCGGACCTACGATTTATTTTCTAGAGGGCAATC
GGAGAAATTAACCAAACTTTTAAGGGGGCAATCAGTGTTTTTGCCTAAAAAT
TACACTAATTTTTTTTACATGGGAGGGGGCGCTCCTACACCAAATAGTAGATC
TGCCACTATTCTCGGTAATATGAAACATCTTGATAGAAAAAAAAAAAAAACCA
TATTTGCTTAAGTTTCTTAAATTCAGCATATCAACATTATCGTTCAAGTATTTT
ATGCAACTAATTACAAAGTATTTAGACATCAGTTAGGCCTTTAACTAAGTACT
TAAATTCCTCTCATGCCTTAGGTAGACACACATCATTGGTTCAAATGACCAAT
AATCAATTTACTCAAATTATTTGTGGCCTCCCTACATATTCCTAATGACATAC
AATTATTTGTGGCCTCCCTACCTACATATTCCTAATGACATACAATTATTTGTG
GTCTCCCTACATATTCCTAATGACATACAATTATTTGTGGCCTCCCTACCTAC
ATATTCCTAATGACATACAATTAGGGGTGTCGTTCGGGTTTTGTAATCAGGTA
TCGGGTTCCTCGGATTGTCTTATTTTGATGTCCTAACCAACTACCAACCCTAA
GTAGGTTTGGTCAGGTAAGTTTGGGCCGGGGTAATTGGTAATCAGGTTTCTAA
TTGGGCTAATAAGTGGGGTTCTTCATTTACCATCAATATGATTTTGGGCTGCT
AAAATTAGGTGGAGAATGATGGACTAGGTGTAGGTGGAATGATCGAGTGTGA
CAAAAACTTGGAGATGAAACTTCGTGGTTGGGGTATCGTTTTGTCAAATGTCA
CGAGAATAGAAGGATCAAAAGGTTGTCGTCGGTTTAAGCGAACACCCATATC
AAATGGTTTGTTTAAATGTGCGATCGGGGTATTCAGGTTGACTCGAATTATAG
TTAATAGAATCGATTAATGAACTGAATTGGGCATGTCGGGTTTGGTTATGATT
GTTAACCGGGTCGGTGAAAACGACACCCCTACATACAATCAGTTGGTTGAAA
ACCAACTTTTGTAAATTACCGAATTCAAACTCATTTAATCTGTTAGTCATTAT
AAAACACCAAAATAGGTTATTTTACATTCCTACTTTAACAACAAAAAAATAC
AACACACAAAAGTCAAAACCAGATTAAGATTTGTCTCTTATAAANNNNNNNN
NNNNNNNNNNNNNNNNNNNNNNNNNNNNNNNNNNNNNNNNNNNNNNNNN
NNNNNNNNNNNNNNNNNNNNNNNNNNNNNNNNNNNTGATGCATATGACGA
ACCACCCTCCGCCACTACGGATCTACTCTTCAGACTCATTGCTTTTACCTTTGT
TTTGACCTCATTGTCCTCCATAACACGTCTTATCCCACGTTCTATCTCCTCAGC

TGTCACAATAATTGTGTCAGCCCTAGGATTAGATACATTCTTCCTATAATCCA
ATTTTATCTCCACCGCCAATCCCAACTCCACTACCATTTCAAACGCATTCATA
TGTTGCTCGGCGTACATCGGCCATGTTGCTAATGGTACACCGAACCATAAACT
CTCCAACAATGAGTTCCACCCACAGTGGGACACAAACCCTCCAACTGCATCA
TGACCCAACACCGCCGCCTGTGGGGCCCACCCAATCACCTTCCCTATTCCACA
TGTTCGCTCCAAAAACCCTTCTGGCAGTACCAAACTCGGATCCTCATAATCAC
TTGGGATCCTTGATGTTTGATCTGACGGAGGTCGACGTAGGGACCACACGAA
ACGATGGCCACTCTGCTCTAAAGCACGTGCTATCTCCTTCACTTGGACCTCAC
TGAAACTTCCCATACTCCCAAAACACAAAAACACAACCGAGGAAGGTGGTTG

>scaffold396498
AGGGAGATAGAAAGTGGGATAAGACGAGTGATGGAGGATAACGAGGTTAGA
GCAAAAGTGAAAGAGATGAGTGCCAAGAGTAGAGCGGCGGTGGTGGAGGGC
GGTTCGTCATATGCATTTTTCAGTCGTCTTATTCAAGATTTTATAAGAGACTA
ACTCTTGATCTGGACTTCTATTTGTTGTGTTTTTTTTTTGTATGTTACTAAAGTT
GGAAGTATTGTTTTCATGTGTTGTTGTAGGAAGGTGAACCACAAAAGTTGTCC
ATATATGAGGTTACTATGGTATAATTATTGTTGCCTTTTTACCCCTTTTTACAA
TCTTGCCTGCTTTACATTTTTTACAGTAATCAACATCGTTTTACTAGGATCTTC
TGTTGGCAAATAAAAAGGTTGCCAGTATCTAGGTTCAAAGCCAATGGGATTT
AATGGGGAATTGTGGTAGACGATGGCTACGCTGGTGCGTTTTGTATGGCAGA
CATGGCAGTAGATGTATTTCCAGTAGGTACCTAGGAGAAGTTTAGATTAGGA
GAAGTAGAGGTGCACAAATCAGGTTTTTGGCTCAAACTGGTTCGGTTGAAAC
CTGGTCGGGTTGGCCCAAAGAAAGTCAAACCCGGTTTTTGGTGAAATAGGTT
CGGTATCTGACCTGGTTTTTTAGGGGAAAGGCTCGGGTTTGACCGGGTACTGG
TTCAGTACGGGTTTGACCAGCTTGAAATTAGGTACTGGCTTGGTTCAGGTTTA
AGCAGGTTCGAACTGATTTTTAAGCATAGGGTTACAAACCGATTTCAACTCTA
AAGTTCGGTTAGGGTTCAACCTAATTGGTTTCAAAATGGGGTTCGATTTGGGT
TTAAAACCTGGATGGTTCACTTAGGAAGAAGGACTTGATTAAGGTAGGATAA
GATCTAGCTAGGAGAGTGGAAACCCACATATATCATTAAAACCAACCAAGAG
TGTGACACTTTGAAATTTAATATTACATTTAATATCAAAGAGAGAAATCTTGG
ATAACTTTAATAGTTAAAATTCATCATTTTATATATATCATTAAAACCAAAAT
GAACCAAGAGTGTGAAACTTTGAAATTTAATATTACATTTAATATCAAAGAG
AGAAATCTTGGATAACTAAGAAGATATAAACTAATAAAAGATGGATGACAA
AGGATAAACAATTAGCTTAAAAGTAAAAGAAGTAAAAGAGATGAGTAAGAA
GAGTAGATCGGTATTCATATNNNNNNNNNNNNNNNNNNNNNNNNNNNNNNNN
NNNNNNNNNNNNNNNNNNNNNNNNNNNNNNNNNNNNNNNNNNNNNNNNNN
NNNNNNNNNNNNNNNNNNNNNNNNNNNNNNNNNNNNNNNNNNNNNNNNNN
NNNNNNNNNNNNNNNNNNNNNNNNNNNNNNNNNNNNNNNNNNNNNNNNNAT
CGGGGACTCTTCACTGCTCCACAATATAGCCGTTAGACATGGAAAAGGACCA
TTGGACATGATGTTAACGGCTATAATACAGTTTAAATTAAAAAAAAACAATT
AAATACATTATAAATATTACCATTTTACATTCCATTTTTCACCCAATTCACATA
CTCTCAAAGCATACTATAGACCGACAAAACAAGGGCGATGACAAGAAAAAA
GTTAACGGTTCCGGTTCCAAGGCGAGGGGGACGGCTCGCAAAGTAGACGAG
GTGGCTCGAGTGGTTCAAGTGTGCCATTTCAATCACAACACGGTTACNNNNN
NNNNNNNNNNNNNNNNNNNNNNNNNNNNNNNNNNNNNNNNNNNNNNNNNN
NNNNNNNNNNNNNAACCTCCATACTTTTACGCCCAAAATCCACAATACGGTT

TTTACAACACCCAACCCCAATCCAACTATCCACCACAATTTTTTTCATTATG
ACAAGGTCCCACGATGGTTGCACCGGGTGTGTACGAGTATCCTACGGAAGCC
CAAAACGCTTTTGACCCGTTTGCTTATCGGAGTCCACCGACTCAATCTCCAAG
AGATAATGTTAAACACATGCTTCCAATATACGATGATGACGAAGATGAAGAA
TTTGTAGCCAAGACTCAAGAGTTAAATGAAGACGAAAATAAAGAAGCCGAA
GAAGAGGAAGAACTTGAAGTCGAATAAGAAGCCGAAAATGAACGGAAAGGA
AAGAGGGGTAAGGCGGATCCGCAAAGATGGACAAAGAAACAAGAAGAGGC
GTTAGCGATGACGTATGTGCATTGTAGTGTTAACAAGAAAAAAGGCAATCAA
CAAAAATTGGAAGATTTTTGGAAAAAAGTTATCGCACAATACAATGCAATCG
TTGGAGGAAATTCACGTTCATCAAGTCCATCCGAAATGGAAAGCTATGCAAG
GGAAGTTTGGGGCAAAAAAGAAAGTTATCGGATTCGGGTAACATCCAAGCG
GGTACACCGAATACTGAATTCACTAGTGATATACCCGACATAAACGTAGACC
CCTCTCCAAGAAGGTAAAAAAAGAAAGAGAAAAAAGATAAAAGACCCGCAA
CGTTGACCGAAAGGTCAATTGGTATGTCCGACAAGCTCCAAGAATACAAATT
TATGAAACAAGAGTTGGTGGATATTAAAAAGAAACGAGAGGAAAAATTTAT
AGAGTTGGCGGATGAACATCGAGAGAGGTTGAAATCGAGTAGGTTTGATAAG
GATATGGATACGTTATGAATGAAACCTTAATTAGTCAAAACAAAATGGAATA
ACCAAATAAAATGCTTTGAATGATAATCAAAGTATTAAAGTGGTTAAATAGA
ATTTATATAGACAACTAGAATCATATTTTATGTAATGATAATCAAAGTATTAA
AGTGGTTAAATACAATTTATATAGACAACTAGAATCATATTTTATGTGGCTCA
TATTTTATGTAATGATAATCAAAGTATTAAAGTGGTTAAATACAATTTATATA
GACAACTAGAATCATATTTTATGTGGCATGTGATATAACTTATTTTTTGTGCTT
TAAAAATTAAAATCGCTCTCAATTTATTTTGAGTAATTTAAAGTATTGGTCAG
TTCAACCTAGGATATGTGTGCCCCATGGGATGAAAGAAATTTAAGTACTTAG
TTTTCATGGACCCTTGTTTGAGCCACCTGATTTGAAAACTGCTCATTTCGAGG
ATATTTGCTTATATAATTCAGTGAAACACTATAATAAAATGGGCCATGTGGGC
CTAAGTTTCCACCTGATTTTCGTCAGCAATGCAATACCATCAACAGGTAATGG
TATATAAACTGAATATATGTATGTATAAGACTCCAAACAAACCATTCATGAA
CCAAAACAAATATGGCTAGTGAAGTTGCAAAGCTAATATTCATCCCTTCCCC
AGGGGTTGGTCACATTATGTCAACCGTTGAGATCGCAAAATCGCTTGTGAAT
CGAGATCAACGTCTCGCAATAACCGTACTTGTCATCAAGCCTCGTAGCCTGG
ATTCTGGCATGGCTATCACCTCCTACATAGAATCATTAGCTAAAAGCAATATG
GATCACATATCCTTCATTCAACTCCCTCAAGATGAAACCTTACTAACTCATGA
CCCCAAAGATCACATGTCTACGTTCAGTGAATTCATCCCTAGCCACTGCAAAT
ATGTTAGAAATGTTGTGGCTGACATGATAAGTCAAGCTGGTTCGGGTCGGCT
CGTTGGGTTTGTCATTGACATGTTTTGCACTTGTATGGTCGATGTGGCTAATG
AGTTCAATGTTCCAACCTACATATACTTCACTTCTAGTGCCGCTTTTCTTGGAT
TTGAATTTTATATCCAGAAACTCTGTGCTGATCTCAATGAAGACGTTATTGAG
TTGAGCAACTCAGATGCCAAGATATCGATTCCAAGTTTTGTTAAACCGGTTCC
AACCAAAGTCTTTTGGAGTCGTGTCAAGACTAGAGAGGGACTAGATTTTGTC
CTGGGGGCTGTCCGGAAATTTAGAGAGGTCAAAGCAATCATGGTGAATACAT
TCTTGGAATTGGAAACACACGCGATAAAGTCGTTGTCTGCTGACGTCAGCAT
CCC

>scaffold359006

GTGGTTGTGTTTTGGCCATGCGTGATTACGGTAAAAACCAACCTGTGCGTGAT
TTTGTGTTTTGGTCATGCATGATTTCTTCTTCATGCGTAATTAGGATTTGAAAT
TGTTAGGTGCATAATTTACAAATGAACATGTGTAATTAAGTCGCGTACCGTCG
CACGTTAAGGAGCTATTTTACGTTAACATTCCCATATATATATTTCGTATATTC
TCTTGTTAATTGCACTACTTTCATGGGTGTCGACTGTCGAAAATACCTTTCATT
TCTTGCAAAATAGTGGGAACACTACAAATAAAAATCCATTCAGTGGCATACA
GTAAGTGCCACAAAAAACCAAAAAAGTGCCACTAAAGGCCCCTGTTGGAATG
ATTGGATGACCATCCGAACGGATTGACATCCGATCGACTGGCCATNNNNNNN
NNNNNNNNNNNNNNNNNNNNNNNNNNNNNNNNNNNNNNNNNNNNNNNNNN
NNNNNNNNNNNNNNNNNNNNNNNNNNNNNNNNNNNNNNNNNNNNNNNNNN
NNNNNNNNNNNNNNNNNCACCGTTTATGCTATCGTTAACTGTTCAGGCTAAT
CTCTCTCAGCGCTCCCTTCAATCCAAGAGAGTGTTTACTTGTTAAACCCAATC
TGTAAGTATACTCGATCCCTTTTTGCTTTACGCACTTTTGGGTGTTACATACGT
TATCTATATTCAAATTACATCAATCACACAACGCAAACACAGTTTATACGCTA
ACCGTTATTGCATGCTACGTGTTATTCGATGAATGCTTGTATGTTATGTTAAC
ACAGTGATTGTTGCCTGACACCTTAGCAACGATAGTACCATAGTTTGGACTCA
GCACCTGTCGTGGACAGGGGTTGTTAAGGGCTTTACTTCACGTATCCCAGTGG
GGATATGTGTTACGCATTCTACAACTCGCAGTCACNNNNNNNNNNNNNNNNN
NNNNNNNNNNNNNNNNNNNNNNNNNNNNNNNNNNNNNNNNNNNNNNNNNN
NNNNNNNNNNNNNNNNNNNNNNNNNNNNNNNNNNNNNNNNNCTCTGTCGATAA
CCTACTTGCCTTCACTTTATACATGTTACATGCCGGTTACGCGTAAACGATTT
CGAACTCTATTATGCTATATCAAACTTGTATGCTCGCCTTTACACTATGTGTAT
TGACCTTTATTTTAACGTATATGACAGGTGTTTAGGATGCTATCTGCTAGGAT
GCCTGCTTTAGTGGAATCAAGTAGGGAGGAGTCAAGAAACAAACCAAAAAC
AATTTATTTATTTTGAATCTGAGTTGTCAGAACATAATTATTTGTCTTTGAATT
GTCTGTAATAACTATGCTACTTGTTATGTCATGGTATGGGACGTGATGCTTAA
ATAATTGGTAATCGTAGTTGTTATGGAAACTCCTGGACAATCTGTTTCACTCA
GTGCCATGCTCTGATGTTTCCGTCATCGGTTGGGATGTGACATGTAAGGGTTA
TAAAATATGTTTTATCACTTTAATAACTTGGTAAATATTATCAGAATATGTAT
AAACATGTTTTAACCCGGCAATTTTGAGTTTAAGCTCGGTTCGTAACCGAAAG
TCGCAAAACTTGACTTTTGCTTTGACTTTCAGTTTTGACTCGAATTAGCATAAT
TTCTTTATGTTATAAGGTTCCTTTGTGACCATATTATGTGGTAGTATAACCCTC
TGAGGTTATATTACATGTTCACATAGTAATCCGAGTTCGTTTCCTTGTTTCCGT
TATTATGCTTAATGTTGACTATTATGCCCTTTTGTTCAAAAAAATTAGATTTTA
AACATAATCAAGTATGCAAATGAATTACATTCTGATTAGTAAACATATTTAAC
ATATTTTGACATTTATAATCTGGTCATAAACTAAGTTTACATAATTAATCGTA
AAATATGCTTTTATAAGGATTTGTAGATACCAAGGTGTATGGAAAGGTGTCG
TCTTCTATAAGGTTAGGGTTCAAGTCTCATAGTGGACAAAAGTCTGAATTTAG
GAGTAAGATTTGAAAATGTGTGAGTTAACCGGTCAAAAAACAAAAACCTTTT
TATTTGCCAACATAATAGTATTGCCATTTGAACTATGATCATTTATACCAACC
TAGTGATATGGAGGTACATGGACCACCTATCAACTCTTAACACTATGTTTGTA
GTTACACCAACACTTTAAAATGAGCAACAAAAAAACACTACAAACAGAAGTC
CAGATCAAGAGATGTCTCTTGTGAGATCTTGAATCAGACAACCAACAGATAC
ATATGACGTACCGCCCTCCACCACAGCAGATCTACTCTTCGCACTCATCTCTT
TCACTTTTCTTCTAATCTCGTTATCCTCCATCAACCGTCTTATCCCACTTTCTAT
CTCCACAGCTGTGACGATGATTGCATTAGCCTTAGGATTAAACAAATCCTTCA

TATAATCCAACTTNNNNNNNNNNNNNNNNNNNNNNNNNNNNNNNNNNNNN
NNNNNNNNNNNNNNNNNNNNNNNNNNNNNNNNNNCCCAGCTCCACCACCA
TTTCAAACGCATTCATCTGTTGCTCAGCGTACATTGGCCATGCTGCCGTTGAT
ACACCGAACCATAAGCTCTCCAACAATGAGTTCCACCCGCAGTGGGACACGA
ACCCTCCAACTGCATCGTGACCCAGCACCTCCGCTTGTGGAGCCCACCCAATC
ACTTTCCCTATTCCATGTGTTCGCTCCAAAAACCCTTCCGGCAGTACCACACT
TGGATCCTCATAATCGCTTGGGGACCTTGATGTTTGATCTGACGGAGGTCGAC
GTAGGGACCACACGAAACGGTAGCCACTTAGCTCTAGTCCATGTGCTATCTC
CTTCACTTGGACCTCGTCAAAACTTCCCATACTCCCGAAACACAAAAACACA
ACCGAGGAAGGTGGTTGACTATCCAACCACCTAACGACGTCATCGTCATAAG
GTTTCCCAGCACCAGAACCACCTTCTAAGTTGAGTATGGGTCCCACCGGATA
CACCGGTGGGATGCTGGTGTCAGCAGACAACGACTCTATCGCGTGTGTTTCC
AATTCNNNNNNNNNNNNNNNNNNNNNNNNNNNNNNNNNNNNNNNNNNNNN
NNNNNNNNNNAAAGTATTGACCATGATTGCTTTGGCCTCTCCAAGTTTCTGC
GCAAACCACCGAACAAAATCCAGTCCTGCTCTAGTCTTGACACGACTCCAAA
ATACTTTGGTTGGCACCGGTTTGACAAAACTCGGAACCAGTATCTCGGTATCT
GAGTTGCTCAACTCAACAACATCAACATCCTTGTTCAAATCATCACAGAGTG
ATTTTATATATAACGTAAATCCAAGAAACGCAGCGTTAGAAGTGAAGAAAAC
GAACGTTGGAACATTAAACTCATTAGCCACATCAATCATGCTGGTGCAAAAC
ATGTCAACAACAAACCCAACAACCCGGCCCGAACCCGGTTGACTTTTCAAGT
CAGCAACAGCATTTCTAACGTATTTGCAGTGACTATTAATGAACTCTATTTTG
AAATTCCTTTGATCTTTCGGGTCAACAATTGGTGGAGTTTCATCTTGAGGGAG
TTGAATGAAGGATATGCGATCGATGGCGTTTTCAGCTAATGATTCTATGTAGG
TAGTGATAGCCGAGCCAGAAGTAGCGCCGGGTGGCTTGATGACGAGGACGGT
TATCGCGAGACGTTGGTCGCGATTCACGAGTAACTTTGCGAGCTCGATGGTTG
ACTTGATATGACCAACTCCAGGGGAAGGGATGAATATCACCTCTGCAACTTC
ATTCGCCATATTTCTTATGTTTATCCACTGTTCCGTGTAGATGATTCCAATTAG
CTTAATTATGTCATTGCGTTGCTGGCGAAAGTCACAAAAATATTAAAAAAAA
AA

>scaffold499821
GATCTCAGAAAACCCAGTTCAACATGATCGAACAAAACACGTGGAGGTAGAT
CGACATTTCATCAAGGAAAAACTGGAGGAAAGAATCATAGAGCTCCCATATG
TTCCGTCAAAAGATCAACTCGCATATATTCTTACAAAGTCGTCAACGGAAA
CACGTTTAGTAGCTGCTTGAGCAAATTAAGTATTGGTGACCCCACTACTCAAC
TTGAGGGGGAGTGTCGGAAAGAAGATAATAAAGATAATAAATTATAGGGTA
GATTTGGATCAGTCAAACACTCAAACCTAATCTCCCAATTACAGCTCACGATC
CGGATTTCCAGCCGTTGATTACTCCCTTTAATTTTTCTTGTATCCATTTTTGTCT
CTGTATTTGGTCTATAAATTTAAAGGCTTGAATGTAATAGAAGATGTATCAAT
CACAACTTACAATTCAATACAAGCCTCTTTTTGATCTCTATACCAGTTGATAT
AGAGCAGAGCGGATACCGTTTCGTGTGGTCCCTACGTCGACCTCCNNNNNNN
NNNNNNNNNNNNNNNNNNNNNNNNNNNNNNNNNNNNNNNNNNNNNNNNN
NNNNNNNNNNNNNNNNNNNNNGGTCCCAAGCGACTATGAGGATCCGAGTGT
GGTACTGCCAGAAGGGTTTTTGGAACGAACACGTGGAATAGGGAAGGTGATC
GGGTGGTCCCCACAGGCGGTGGTGCTGGGTCATGAAGCAATTGGAGGGTTCG
TGTCCCACTGCGGGTGGAACTCATTGTTGGAGAGCTTATGGTTCGGTGTACCA

ACAGCAACATGGCCGATGTACGCTGAGCAACATATGAACGCGTTTGAAATGG
TGGTGGAGTTGGGTCTGGCGGTGGAGATAGAGTTGGATTATATGAAGGATTT
ATATAATATTAAGGCTAATACAATCATTGTGACAGCTGAACAGATAGAAAGT
GGGATAAGACGGTTGATGGAGGATAACGAGATTAGAAGAAAAGTGAAAGAG
ATGAGTGCGAAGAGTAGATCGGCTGTGGTGGAGGGCGGCTCATCATATGCAT
CTATTGGTTGTCTGATTCAAGATTTTGTAAGCGACAACTTTTGATCTCAACTTC
TGATTGCTGTGTTTTCTTGTTACTAAAGTTGGAAGTGTTTTTTTTTTTTTTTCA
AGTGTTGATGTAACCACAAACATAGTGTTGATAACAATCTTACTTTATGGTTC
GAGTTCAAAACCAGTTTCAGTTCCTAACCCCGGTTTTGTGCACCTCTAACTC

>scaffold588662
ACTTCCATTAGTCCCAAAACACAAAAACACAACCGAGGAAGATGGTTGACTA
TCCAACCACCTAACGACGTCATCGTCAAAAGGTTTCCCTCCACCAGAACCGC
CTTCTAAGTTGAGTATGGGTCCCACCGGATACAAAGGCGGGATGCTGGTGTC
AGCAGACAACGACTCTATCGCGTGTGTTTCTAGATCCAAGAATGTATTAACG
ATGATTCCTTTTGCCTCTCCAAGTTTCCGGGCAAACCACTGAACAAAATCCAG
TCCAGATCTAGTCTTGACGCCACTCCAAAATACTTTGGTTGGCACCGGATTGA
CAAAGCTTGGAACCAATATCTCGGTTCCTGAGTTGCTCAGGTCAACAACATTT
CTGTTCAGATCATCACAGAGTGATTTTATATATAACGTAAATCCAAGAAACG
CAGCATTAGAAGTGAAGAAAACGAAGGTTGGAACATCAAACTCATTAGCCAC
ATCAATCATGCTGGTGCAAAACATGTCAACGACAAACCCAGCAACCCAACGC
GAACCTGGTTGACTTTTCAAGTCAGTAACCGCATTTCTAACATATTTGCTGTG
ACTTTTGATGAACTCTATTGGGGAATTGCTCGGATCTTTTGGGTCATGAAACG
GTTGGGTTTCATCTTGAGGTAGTAGAATGAAGGATATGTGATCCATGTTGTTT
TCAGCTAATGATTCTATGTAGGCAGTGACGGNNNNNNNNNNNNNNNNNNNN
NNNNNNNNNNNNNNNNNNNNNNNNNNNNNNNNGAATTCACGCTAAGAGGCT
TGATGACAAGGACGGTTATGAAGAGACGTTGATCTCGATTCACAAGTAATTT
TGCGATCTCCATGGTTAACTTGAGATGACCGACCCCAGGGGCAGGGATGAAT
ATTAGCTCTGCAAGTGTATTAGCCATATTTTTTTGGTTCATGAATGTTTTCTT
ATGGTTATATTTATCCACCATTCCTTGTAGATGCTACTACCAATTAGCTGATG
TCATTGCATTGATGGAGAAAGTCACAAGAAAATATTATATACAACATTTCTA
CATTTTTGTCTCTTTTCTTAATAACAAAATCAAGTATATTTGTTGGAATATTTA
CATCATTAGAATTAGTTCAGAATGAATCTAAGCTTGGAACTTTTCTGATATCT
AAGTAACTTTTTTTTTTCACAACAAATTATATTAACAAAACATGTAAAACTAT
TACACAAACAAAATTTTATATACCCTTTCCATCATCAGCTAATCCTAAAATAA
TAGACATAGCTTTTTCCTACACTTTTGTTTGCCTTAGATCTGTCTTGGTGTAGG
TACTTTAGGGGAGCCGACAGGGGGGTTCGTATTACTGTTTCTTGCTTATTTGC
TCTTGAGTAAATTACGTTTTGAGTCCCTGTGTTATAGTAGTTTTAACCATTTAA
GTCCAAAATCAAAAAGTTTAACGCCCTAAGTCCCTAACCATTGATTTTATAAC
GTTGAGTCTCTATGTTTTAGTGGGTTTAACCACTTAAGTCTAAAATCAAAAAG
TAGAAGTGTTAAAAATTGGACTCAAAATGTTATAAGATAAGTGGTTAGGGAC
TCATGACGTTAAAATTTTTGATTTTGGACTTAATAAAACAAATGGACTCAAAA
AGTAATTTTTTTAGGGTGTGCCTATTGGCACACAAAAGTGCCTATTGGCACAC
CAAACCCTAGCAAAATTAGGGTTTAT

>scaffold433233

TTCAAGAAGCTCTTAAAGATCACGTAAAGGGTAAAAAGTTTCTGTTGGTACTT
GATGACATATGGAGCGAAAGTTATGAGGATTGGGAAACCCTCGTTAGACCGT
TGTATACATGTGATCCTGGAAGCAAGATCATCATAACAACTCGGAAGGATAA
GTTGCTTAAAAAACTGGGTTACAATCCTCTAAACAAGCAGCTGGAGAGCCTC
TCAGATGATGACGCCTTGTCTTTAGTCGCTCGAAATGCATTAGGTGTAGATAA
CTTTGATTCACATTTGTCGCTCAAACCATATGCTGAAGGTGTTGTAAAAAAAT
GTGGAGGATTACCTTTAGCTTTAATAGCACTTGGTAGATTGTTGAGAACAAA
AAAAGACGAAGTAGAGCACTGGAAGGAAGTGTTAAACAGTGAGATATGGAG
ATTAAATAATGAAGATGGAATTCTCCTAGCCCTTAGATTGAGCTACCATGATT
TGTCTGCACCTTTGAAGAGGTTGTTTGCATACTGCTCATTGTTTCCCAAGGAC
TTCCTGTTTGATAAGGAGGATCTGGTTTTACTTTGGATGGCAGAAGGGTTCTT
GCAGCAGGCAACTCCAAATGATTCAACAGAAGAACAATGCTTGGGTCGCAAA
TTCTTTGACGAGTTGTTGTCAAGGTCGTTTTTCAACATGCACCTAATAATGA
ATCATTATTTGTGATGCATGACCTCATGAATGACTTGGCAACATCTGTTGGTG
GTGAGTTTTTTGTAAGGTTAGACGGTGAGGCAAAGAAGGATATTGGTGTAGA
GAGGTTGAAGTACCGCTATATGTCATTTGTTCGTGAGACATATGTAACTTACG
AGAAATTTAAAGGATTCACCGAAGCCATAAAATTGAGGACATTCTTGGCAAC
GTCTGTTGGGAAGGTTGAAAGTTGGAGAAGTTGCTACTTATCCAGTAAGATT
CTTACTGACTTGCTTCCTGAGTTACCATTACTAAGGGTCCTAAGTTTGAGCAA
TTTTGAAATAAGTGAGGTGCCGGAGTCCATTGATACATTGAGGCACTTGAGA
TATCTTAATCTATCTCGAACTCGTATCACACATTTACCAGAGAAAGTTTGCAA
TCTCTTTAATTTACGAACATTGATCCTGTTTGGTTGTGATAGATTAACTAAGTT
GCCCAACAATTTCCTAAAGCTTAAAAACTTGCGACATCTTGACGTTAGGGAC
ACCAAGCTTTTGTTTAAGATGTTGTTAGAGATTGGTAACTTGAAAAGCCTACA
AATTTCTCTCTCGAAAATTGATATCGAAAGTGAAAGCGGAATTGAAATAGCC
AAACTTAAAGACTTTGAGAATCTATGTGGGAAAATTTCTGTTGTAGGTTTGGA
AAAAGTGCAAAATGCAATTTATGCACATGAGGCGAACTTTTCAGAAAAGAGG
CTTAGTAAGTTAAAGCTTGTATGGGGTGATGAGCTAAATGATTCTCGAAATG
ACATGCTTGAAAAGGAGGTTCTAAATGAGCTGAAGCCTTGTCATGATAAGTT
AATAAAACTCAAACTTATGTCATATGGGGGATCAGAGTTTCCAAAGTGGGTT
GGGGATCCTTTGTTTCTTCATTTGAAACATGTGTCAATAAGTGGGTGTAAGAG
ATGTACATCAATACCGCCATTGGGACAACTACCGTCACTGAAGGAGTTGTTA
ATTAAAGGCTTACATGCAGTGGAAGTTGTGGGTTTTGAGTTATTTGGGACTGG
TCACGCATTTCCTTCTCTTGAAATTCTGAGTTTTGATGATATGGGTGGGTGGA
AGAAATGGTCAGGGGCTGTGTTTCCACGCTTGCAAAAGCTTGAAATAAGTGA
TTGTCCTAATTTGGTTGAAGTCACACTTGAAGCAATGCCTTCATTGAATGATC
TAAGAATAAGTAAATGTGACAGTGGTGTGTTGAGAAGTCTGGTTGAAGTAGC
ATCAGCAGTCACCAAGTTGGATATAAATGGTATTTCAGGGCTTAATGATGTG
NNNNNNNNNNNNNNNNNNNNNNNNNNNNNNNNNNNNNNNNNNNNNNNNNN
NNNNNNNNNNNNNNNNNNNNNNNNNNNGTTACAGAGTATCTTGGGGCAGTTG
AAAAATTATACATATATGGATGTAAGGAAATAAGGTACCTGGTGAAATCAGA
TGCAGATGCAAGTAAGATTCTTGTGAAGCTGAGGAAATTGGAAGTGAAGGAA
TGTGATAATTTGGTGAGTATAGGAAAGAAAGAAGAGGAGGAGGAGGATAAT
TGTAGGAGCAACCTCCTAACATCTCTTAGGATGTTGTTTGTGTCTCATTGTAA
GAATATGGAGCGTTGCAGCTGTCCAGATGGCATTGAGGAGTTGACTGTCTCTT
ATTGTAGTTCAATGACAGTTGTCTCGTTTCGAAAAGGAGGACAGGANNNNNN

NNNNNNNNNNNNNNNNNNNNNNNNNNNNNNNNNNNNNNNNNGAGGTCACT
TAGAATATGGAATTGCTGGAACCTATTGGAAAAGGAGTGGGGAGGAGAAAA
GACGAACAACAGAAGCAGCATGCCCATGCTTGAACATGTATCTATAATTGGT
TGGCCAAATCTGAAATCAATCATTGAATTGAACTGCTTGGTTCACCTCACTGA
ATTGATAATACAAAATTGTGAAAACCTTGAGTCATTTCCAGACACTTTAAAAT
CGTTAAAGAAACTGGAAATAAGAAATTGTCCCAAATTGGATGTTTCCTGTCTT
GGTGACAATTTGCCATCATTGAAGGAACTGGAAATAAGAGAGTGTCCAAGAA
TGGATGCTTCTTTACCTGGTTGGGTTTGGCCTCCCAATTTGCAAATCTTAGTA
ATTGACAAGTTGAAGAAGCCCTTCTCCGAGTGGGGCCCACAAAGTTTTCCAA
CCTCACTTGTGGCACTATCCTTACACGGTGGTGATGAAGATGGAGTAATTATT
AGTTGTAGTGANNNNNNNNNNNNNNNNNNNNNNNNNNNNNNNNNNNNNNNNN
NNNNNNNNNNNNNNNNNNNNNNNNNNNNNNNNNNNNTTCATCTCTTACTTCTC
TTGAAATAACTGAATTTAAGAACTTGGAATCAGTTTCAATGGGACTCCAACA
CCTCACCTCCCTCCAACATCTCTCTTTTAGCTTTTGCCCTAATCTGAAGAAAGT
GTCTTCCCATCCCCAGCAACTCACCTCCCTCCACCATCTCACTTTTCTCAATTG
CCCAAAGATGATGGATCTACCAGAACTATTGTTGCCTTCACTCTTGAGTTTAA
ACATCTTGGGTGATTGCCCAGGAGGTTTGAAAGAAAGATGCAGTAAAAAGGG
GAGTTACTGGCCCCTCATCTCCCATATCCCCTGTATCCGCATACGAGATTAAC
ATATGAAGGTCTCTCTCCATCTGCCTTTAATAATTTTAAATTAAAT

>C16653159
CTAATGCATTTCGAGCAACTAAAGACAAGGCGTCATCATCTGAGAGGCTCTC
CAGCTGCTTGTTTAGAGGATTGTAACCCAGTTTTTTAAGCAACTTATCCTTCC
GAGTTGTTATGATGATCTTACTTCCAGGATCACATGTACACAACGGTCTAACA
AGGGTTTCCCAATCCTCATAACTTTCGCTCCATATGTCATCAAGTACCAACAG
AAACTTTTTACCCTTTACATGATCCTTAAGAGATTCTTGAAGCCGATTAAAAT
CTGAAACTTCCTTGTTTGCCATAGATTGAAAGATCTCTTTGCTAATACGAACG
GTGTCAAACTCATCAGAGACACAAACCCATGCTTTTAGATCAAAGTGATCCTT
GACCTGTTGTTCATCATAGACAAGTCTAGCGAGAGTTGTTTTTCCAACCCCAC
CCATACCGACTATGGGAACAATGCTATACTTTAGATCACATGGTTCATCAGCC
GTTAACAATAACTGTTGAACCAATGCATCTTTCTGAGCTTGGCGTCCAACAAT
ACTAGATGGGTTGACAACAGAGGTTTGCAATCTTCTGTTATTATTAGTTCTTG
GCCTGGTTTCCTCTTCCACTTTCAAACCAAGATTATCTAAACCAAGGTCAGCT
TTCTCCTTAACTAGATCTTGTAGTTTGGTGGAAATATCATCTAACTCAGCAAT
CATAGTAGTGGTCCGTGAGAAAGTTGTGCAACAAGTTGGGGTGATTAGCTTT
CTAACCTTGCCGGTGATGCCTTCTGGCTCTTGCTTGAACTCAGGGTCCATAGC
GTCGATAGCCCAACCATCGAGGAGGTCCTCAATGTCATAAGCCAGATGCTGG
AGATCATTCAGGCGCTGTTTCACAGATGGATCAGTTATCTCCATCTGAGAAGC
ATGAGTAAGATAAAGTTGGATGGAGACTAATGATTTCTGCAATTTCTTGATCT
CAGCATCAATTCCCTTGTGGCGAGCAATGGTTTTCAAAGCTGCATCAGTCAGC
TTGTCAAAGAGGACAGGGAGGAGGGAAGAAAGCACGACTTCAGCCATTGGT
TGCTTACGGTGGTGAGATTGTAAATGAAAGGTATGATGTTGTGAATGCTTGA
GAGTTAAGAGTTGTGTAAAGTGGTTCAGATATTCATTCAGATATAAGGTCAA
CGTTAATGTTTTTCACACTCAGATCAGATCACCAATCAGCATGCAGGGCGGTA
CCTACCATGGCACCCTTAATATTTTAAGTGAATCATTTTGGAGTAATCTGCCT
ACACCGTTAAAAAAGAAAAGAAATTATAAGATATTTTCTTTTAAATATATAC

CCATCTATTGTTAACAAATCAGCACCAATAATTGATCTCTTTTCTTTCGTATTT
ACACCCTAAAGACTTTTACAATTACATAAAAGAATGATTAAACATAAGTTCT
AAATACAGGTGTGAATGATAACACGACACGAACTTAACATGAAATTCACGAC
TTAGAGTTCTGTTTGATCGGCGGAAAGTTCGTTGGTTTAGTTAATGCACGACC
AGAAA
